# Using common genetic variants to find drugs for common epilepsies

**DOI:** 10.1093/braincomms/fcab287

**Published:** 2021-12-04

**Authors:** Nasir Mirza, Remi Stevelink, Basel Taweel, Bobby P C Koeleman, Anthony G Marson, International League Against Epilepsy Consortium on Complex Epilepsies, Bassel Abou-Khalil, Pauls Auce, Andreja Avbersek, Melanie Bahlo, David J Balding, Thomas Bast, Larry Baum, Albert J Becker, Felicitas Becker Bianca Berghuis, Samuel F Berkovic, Katja E Boysen, Jonathan P Bradfield, Lawrence C Brody, Russell J Buono, Ellen Campbell, Gregory D Cascino, Claudia B Catarino, Gianpiero L Cavalleri, Stacey S Cherny, Krishna Chinthapalli, Alison J Coffey, Alastair Compston, Antonietta Coppola, Patrick Cossette, John J Craig, Gerrit-Jan de Haan, Peter De Jonghe, Carolien G. F de Kovel, Norman Delanty, Chantal Depondt, Orrin Devinsky, Dennis J Dlugos, Colin P Doherty, Christian E Elger, Johan G Eriksson, Thomas N Ferraro, Martha Feucht, Ben Francis, Andre Franke, Jacqueline A French, Saskia Freytag, Verena Gaus, Eric B Geller, Christian Gieger, Tracy Glauser, Simon Glynn, David B Goldstein, Hongsheng Gui, Youling Guo, Kevin F Haas, Hakon Hakonarson, Kerstin Hallmann, Sheryl Haut, Erin L Heinzen, Ingo Helbig, Christian Hengsbach, Helle Hjalgrim, Michele Iacomino, Andrés Ingason, Jennifer Jamnadas-Khoda, Michael R Johnson, Reetta Kälviäinen, Anne-Mari Kantanen, Dalia Kasperavičiūte, Dorothee Kasteleijn-Nolst Trenite, Heidi E Kirsch, Robert C Knowlton, Bobby P. C Koeleman, Roland Krause, Martin Krenn, Wolfram S Kunz, Ruben Kuzniecky, Patrick Kwan, Dennis Lal, Yu-Lung Lau, Holger Lerche, Costin Leu, Wolfgang Lieb, Dick Lindhout, Warren D Lo, Iscia Lopes-Cendes, Daniel H Lowenstein, Alberto Malovini, Anthony G Marson, Thomas Mayer, Mark McCormack, James L Mills, Nasir Mirza, Martina Moerzinger, Rikke S Møller, Anne M Molloy, Hiltrud Muhle, Mark Newton, Ping-Wing Ng, Markus M Nöthen, Peter Nürnberg, Terence J O’Brien, Karen L Oliver, Aarno Palotie, Faith Pangilinan, Sarah Peter, Slavé Petrovski, Annapurna Poduri, Michael Privitera, Rodney Radtke, Sarah Rau, Philipp S Reif, Eva M Reinthaler, Felix Rosenow, Josemir W Sander, Thomas Sander, Theresa Scattergood, Steven C Schachter, Christoph J Schankin, Ingrid E Scheffer, Bettina Schmitz, Susanne Schoch, Pak C Sham, Jerry J Shih, Graeme J Sills, Sanjay M Sisodiya, Lisa Slattery, Alexander Smith, David F Smith, Michael C Smith, Philip E Smith, Anja C. M Sonsma, Doug Speed, Michael R Sperling, Bernhard J Steinhoff, Ulrich Stephani, Remi Stevelink, Konstantin Strauch, Pasquale Striano, Hans Stroink, Rainer Surges, K. Meng Tan, Liu Lin Thio, G. Neil Thomas, Marian Todaro, Rossana Tozzi, Maria S Vari, Eileen P. G Vining, Frank Visscher, Sarah von Spiczak, Nicole M Walley, Yvonne G Weber, Zhi Wei, Judith Weisenberg, Christopher D Whelan, Peter Widdess-Walsh, Markus Wolff, Stefan Wolking, Wanling Yang, Federico Zara, Fritz Zimprich

**Affiliations:** 1 Department of Pharmacology & Therapeutics, Institute of Systems, Molecular and Integrative Biology, University of Liverpool, Liverpool L69 3GE, UK; 2 Department of Genetics, Center for Molecular Medicine, University Medical Center Utrecht, Utrecht 3584 CX, the Netherlands; member of the ERN EpiCARE; 3 Department of Child Neurology, University Medical Center Utrecht Brain Center, Utrecht 3584 CX, the Netherlands; 4 School of Medicine, University of Liverpool, Liverpool L69 3GE, UK

**Keywords:** epilepsy, drug repurposing, GWAS, genomics

## Abstract

Better drugs are needed for common epilepsies. Drug repurposing offers the potential of significant savings in the time and cost of developing new treatments. In order to select the best candidate drug(s) to repurpose for a disease, it is desirable to predict the relative clinical efficacy that drugs will have against the disease. Common epilepsy can be divided into different types and syndromes. Different antiseizure medications are most effective for different types and syndromes of common epilepsy. For predictions of antiepileptic efficacy to be clinically translatable, it is essential that the predictions are specific to each form of common epilepsy, and reflect the patterns of drug efficacy observed in clinical studies and practice. These requirements are not fulfilled by previously published drug predictions for epilepsy. We developed a novel method for predicting the relative efficacy of drugs against any common epilepsy, by using its Genome-Wide Association Study summary statistics and drugs’ activity data. The methodological advancement in our technique is that the drug predictions for a disease are based upon drugs’ effects on the function *and* abundance of proteins, and the magnitude *and* direction of those effects, relative to the importance, degree *and* direction of the proteins’ dysregulation in the disease. We used this method to predict the relative efficacy of all drugs, licensed for any condition, against each of the major types and syndromes of common epilepsy. Our predictions are concordant with findings from real-world experience and randomized clinical trials. Our method predicts the efficacy of existing antiseizure medications against common epilepsies; in this prediction, our method outperforms the best alternative existing method: area under receiver operating characteristic curve (mean ± standard deviation) 0.83 ± 0.03 and 0.63 ± 0.04, respectively. Importantly, our method predicts which antiseizure medications are amongst the more efficacious in clinical practice, and which antiseizure medications are amongst the less efficacious in clinical practice, for each of the main syndromes of common epilepsy, and it predicts the distinct order of efficacy of individual antiseizure medications in clinical trials of different common epilepsies. We identify promising candidate drugs for each of the major syndromes of common epilepsy. We screen five promising predicted drugs in an animal model: each exerts a significant dose-dependent effect upon seizures. Our predictions are a novel resource for selecting suitable candidate drugs that could potentially be repurposed for each of the major syndromes of common epilepsy. Our method is potentially generalizable to other complex diseases.

## Teaser

Mirza et al. develop and use a novel genomic method to predict the relative efficacy of drugs for each of the major types and syndromes of common epilepsy. This dataset is a novel and valuable resource for selecting the best candidate drug(s) to repurpose for these epilepsies.

## Introduction

A total of 50 million people are affected by epilepsy.^1^ Current drug treatments for epilepsy fail to control seizures in ∼30% of patients^2^^,^^3^ and cause adverse effects in ∼88% of patients^4^^,^^5^; ∼20% of people with newly diagnosed epilepsy discontinue their first antiseizure medication (ASM) because of intolerable adverse effects.^6^ Hence, there is a need for new ASMs with higher efficacy and/or lower toxicity. Drug repurposing—treating a disease using drugs already licensed for other conditions—offers the potential of significant savings in the time and cost of developing new therapies. Numerous drugs licensed for other conditions have the potential of antiepileptic efficacy.^7^ In order to select the best candidate drug(s) to repurpose for epilepsy, it is desirable to predict the relative clinical efficacy that drugs will have in people with epilepsy. One established strategy for discovering potentially effective drugs is to, first, identify the proteins that underlie a disease and, then, identify the drugs that affect the disease-proteins. In such analyses, genes associated with a disease are routinely used as proxies for disease-proteins.^8^

Genetic factors can contribute to the development of epilepsies, either as single-gene mutations in rare monogenic epilepsies, or as multiple genetic variants in common epilepsies.^9^ Common epilepsies are complex traits with a polygenic origin, which means that the combined effect of many common risk variants contributes to their genetic risk.^9^ Common epilepsies are divided into different types and syndromes^10^; for brevity, we use ‘forms’ as a general term for both types and syndromes. Different forms of common epilepsy have important differences in their genetic determinants,^11^ clinical manifestations and response to medications.^12^ Hence, to be most useful for common epilepsies, methods of drug prediction must use the specific genes/proteins underlying a particular form of common epilepsy, to make drug predictions that are specific for that particular form of common epilepsy. This has not been achieved by any of the published drug prediction studies for epilepsy.^11^^,^^13–17^ Some studies have pooled genes/proteins associated with different forms of epilepsy (including rare epilepsies), to produce a single list of drug predictions for all forms of epilepsy^15–17^; these methods are not readily adaptable to individual common epilepsies, as they require a large number of genes/proteins definitively associated with a disease. Other studies have used genome-wide transcriptomic analysis of human brain tissue from epilepsy surgery^14^^,^^15^; such tissue is only available for a very limited number of epilepsy syndromes, and its analysis is hindered by the lack of suitable control brain tissue that is comparable, normal and has been exposed to ASMs. Of course, any transcriptomic changes detected in epileptic brain tissue could be a consequence, rather than a cause, of disease.

The Genome-Wide Association Study (GWAS) is becoming an increasingly powerful tool for revealing the distinct genetic determinants of different common epilepsies.^11^^,^^18–20^ GWAS results are routinely used to predict new candidate drugs for complex diseases. In the standard approach, significant variants from the GWAS are mapped to genes; drugs that are known to affect the (protein products) of the genes, are predicted to affect the disease.^21^ This simplistic approach has a number of methodological deficiencies. It reflects neither the polygenicity of common diseases, nor the polypharmacology of common drugs. It ignores drugs’ effects on disease-protein abundance, even though, in order to exert their therapeutic effect, drugs rectify the activity of disease-proteins by modulating their function or abundance or both.^22–24^ It disregards the magnitude and direction of change in disease-proteins’ activity, and drugs’ effects upon it. Potential causal variants below the genome-wide disease significance threshold are ignored. Practically, it produces an unordered and unranked pool of drug names, with no indication of the relative predicted efficacy of the compounds, to enable selection of the most promising candidates. Ultimately, it is liable to producing poor results. Some limitations of the standard approach are addressed by recently developed enhanced techniques for using GWAS results to identify effective drugs,^25–28^ but these newer methods and their drug predictions for common epilepsy still leave room for improvement. None of the existing methods make drug predictions for a disease based upon drugs’ effects on the function and abundance of proteins, and the magnitude and direction of those effects, relative to the importance, degree and direction of the proteins’ dysregulation in the disease. Our aim was to develop such a method, and to use this method to predict the relative efficacy of drugs for each of the major types and syndromes of common epilepsy, and to make our predictions available as a novel resource for selecting suitable candidate drugs that could potentially be repurposed for each of the major types and syndromes of common epilepsy.

## Materials and methods

Methods are summarized below; further details can be found in the Supplementary methods.

### Overview

#### Epilepsy types and syndromes

The common epilepsies are divided into different types, which are further subdivided into different syndromes.^10^ In the current work, we included the main types and syndromes analysed in the most recent epilepsy GWAS^11^:

- All epilepsy, which is comprised of generalized, focal and unclassified epilepsies
- The two main types of all epilepsy: generalized epilepsy (GE) and focal epilepsy (FE)
- Two GE syndromes: juvenile myoclonic epilepsy (JME) and childhood absence epilepsy (CAE)
- A FE syndrome: FE with hippocampal sclerosis (HS)

#### Method summary

**Figure 1:**
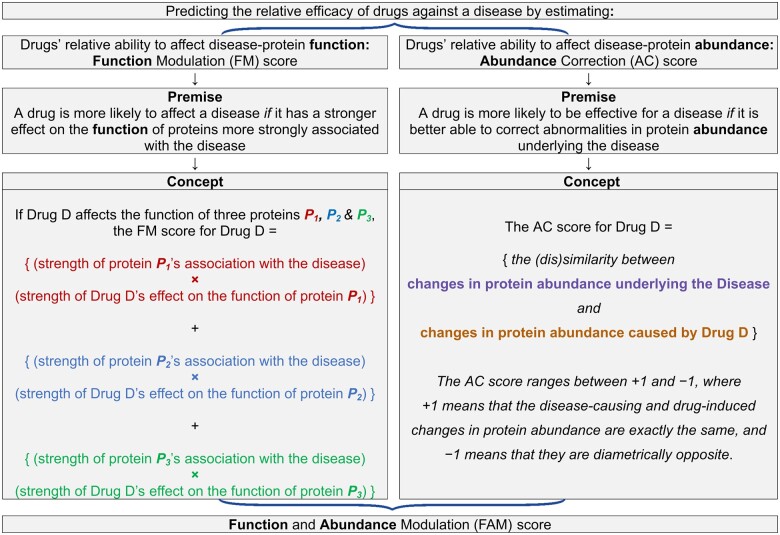
**Premise and conceptual explanation of the disease-protein function modulation (FM) and abundance correction (AC) scores, which are integrated to form the disease-protein function and abundance modulation (FAM) score.** Before integration, the FM score is adjusted to control for the different number of proteins affected by each drug (see Supplementary material for details). Cosine distance is the (dis)similarity metric used for calculating the AC score.

Genetic variants cause disease by modifying the function or abundance (or both) of proteins derived from the variant genes.^29^ Drugs exert a therapeutic effect by rectifying the abnormal function or abundance (or both) of the proteins underlying a disease.^22–24^ To predict the relative efficacy of drugs against a disease, we developed (Fig. 1) a novel score for drugs’ relative ability to affect the protein function and abundance changes caused by common genetic variations associated with the disease: the disease-protein function and abundance modulation (FAM) score.

For method development and benchmarking, we used the all epilepsy GWAS. Then, we applied the developed method to the GWAS for specific epilepsy types and syndromes.

It should be noted that, to aid brevity and readability, we use the expressions ‘disease-associated proteins’ and ‘disease-proteins’ as proxies for ‘proteins encoded by genes bearing variations associated with the disease’, and we use the expression ‘protein abundance changes’ as proxy for ‘changes in gene expression’.

### The disease-protein FAM score: creation and benchmarking

The steps taken in developing the method for calculating the FAM score are detailed in Supplementary material. Below, we summarize the method (Fig. 1) we developed for calculating the FAM score.

The FAM score is calculated by aggregating its two constituent scores:

- The disease-protein function modulation (FM) score
- The disease-protein abundance correction (AC) score

#### FM score

The FM score is based on the following premise: A drug’s ability to affect a disease can be predicted from:

- the degree of disease-association of *each* protein whose function is affected by the drug, *and*
- the strength of the drug’s effect on the function of *each* of those proteins

The degree of disease-association of proteins is derived from GWAS gene-based *P*-values. The strength with which drugs affect proteins’ function is derived from drug-target affinity data. Figure 1 presents a conceptual explanation of how the FM score is calculated from these two types of data. A more detailed explanation can be found in the Supplementary material.

#### AC score

The AC score is based upon the following premise: A drug is more likely to be effective for a disease if it is better able to rectify the protein abundance changes underlying the disease.^30^ Disease- and drug-induced transcriptomes were compared in order to predict each drug’s relative ability to rectify disease-associated protein abundance changes, as previously described^11^ and detailed in the Supplementary material. Briefly, the AC score for a drug is calculated as follows: For each disease-associated protein, the algorithm compares the magnitude and direction of change in the protein’s abundance found in the disease, with the magnitude and direction of change in the protein’s abundance caused by the drug. Then, drugs are ranked in accordance with their *overall* predicted corrective effect on the abundance of *all* disease-associated proteins. To measure the overall effect, a metric called ‘cosine distance’ is used.^31^

#### Aggregating the FM and AC sores to generate the FAM score

The FM and AC scores were converted into their respective z-scores. The FAM score for each is calculated by averaging its FM and AC z-scores (see Supplementary material for details).

### Comparing our method with existing alternative advanced methods

We compared our results with the results from two existing and contrasting advanced methods for GWAS-based drug predictions.

#### Network-based method

An approach employed in a number of studies is to identify the drugs that target genome-wide significant disease-proteins and, in addition, the drugs that target the proteins interacting with genome-wide significant disease-proteins.^32–34^ We used the GUILDify v2.0 Web Server^35^ to identify such drugs.

#### Gene-set analysis method

In this method,^36^ GWAS gene-based *P*-values are first converted to z-statistics and, then, a single-sided two-sample *t*-test is used to determine if the mean z-statistic of the genes that are altered in function by a drug is lower than the mean z-statistic of the genes that are not.

### Validation of the FAM score

For *in silico* validation of the FAM score, we examined the following hypotheses:

- The FAM score for all epilepsy specifically prioritizes the drugs that are effective in people with epilepsy: when drugs are ranked by their FAM score for all epilepsy, drugs used to treat epilepsy are ranked higher than drugs used to treat any other human disease
- The FAM score predicts which ASMs are more clinically effective, and which ASMs are less clinically effective, for each common epilepsy syndrome studied
- The FAM score predicts the observed patterns of relative efficacy of individual clinically-effective ASMs for each common epilepsy syndrome studied

The above hypotheses are further detailed in Results and in Supplementary methods.

To test the above hypotheses, we used the following metrics:

1. Identification of effective drugs: we used area under receiver operated characteristics curve (AUROC) analysis to determine the accuracy with drugs’ scores discriminate ASMs from all other drugs, or discriminate more from less clinically-effective subsets of ASMs. AUROC was calculated using the package PRROC (version 1.3.1)^37^ in R (version 3.4.3). In assessing the discrimination of ASMs from all other drugs, there is a marked class imbalance, because a very small fraction of all drugs are ASMs. To correct for this imbalance, we employed the standard technique of random under-sampling, which is commonly used in published studies (see Supplementary material for further details and references). Specifically, AUROC was calculated using the set of ASMs and a randomly selected set of other drugs equal in number to the ASMs. This process was repeated 1000 times, and mean (± standard deviation) AUROC was calculated. When discriminating more from less effective ASMs, class imbalance is not an issue and, hence, random under-sampling was not employed.
2. Prioritization of effective drugs: amongst all the drug predictions for a phenotype, we determined the average rank of ASMs, or compared the average rank of more clinically-effective and less clinically-effective ASMs. To ease conceptualization and interpretation of results, we converted ranks to percentile ranks. For example, a drug with a percentile rank of 90 is ranked higher/better than 90% of all drugs. Like numerous published studies, we used the median in order to compute the average of ranks, as it is less liable to skewing by outliers (see Supplementary material for further details and references).

#### Statistical analysis

We determined the statistical significance of drug identification and prioritization results by comparing the results to those from a null distribution generated by performing 10^6^ random permutations of the scores assigned to drugs.

### Determining whether the drug predictions are driven by individual highly disease-associated proteins

For each epilepsy, FAM scores were re-calculated after excluding, one at a time, the top 10 most strongly disease-associated proteins (Supplementary Table 3). Drug ranks obtained after excluding a protein were compared with the original drug ranks, using Kendall’s τ. Kendall’s τ is a commonly used measure of rank correlation.^38^ Kendall’s τ ranges from +1 to –1, where +1 means that two ranked lists are identical to each other, –1 means that they are the exact inverse of each other, and 0 means that there is no relationship between them.

Further details about this analysis can be found in the Supplementary material.

### Top candidate drugs

To aid the selection of suitable candidate drugs for experimental validation and clinical evaluation, we demarcated the most promising candidate drugs for each phenotype: the topmost drug predictions with the greatest enrichment of (more) effective ASMs for that phenotype. A manually curated selection of top candidate drugs for different forms of common epilepsy was also produced.

### Testing top candidate compounds in an animal model

As we used complex genetic data to make our drug predictions, we used a complex genetic model to test our drug predictions. We used a rodent model with a complex genetic seizure disorder^39–42^ that manifests as audiogenic generalized seizures: the DBA/2 mouse. We tested the five most highly ranked predictions for GE, after filtering out known ASMs, compounds with existing published evidence in the DBA/2 mouse model, drugs lacking evidence of blood–brain barrier permeability, drugs lacking evidence of safe long-term oral use in humans, compounds insoluble in water or saline and ‘controlled substances’ that require exceptional legal authorization for procurement under the laws of France, where the animal experiments were performed by a contract research organization.

The animal experiment protocol followed the method described by Dürmüller et al.^43^ The study was conducted in compliance with Animal Health regulations, in particular:

- Council Directive No. 2010/63/UE of 22 September 2010 on the protection of animals used for scientific purposes and French decree No. 2013-118 of 1 February 2013 on the protection of animals;
- Porsolt facility accreditation for experimentation (E 53 1031, renewed on 19 April 2016);
- The recommendations of the Association for Assessment and Accreditation of Laboratory Animal Care of which the accreditation was granted in June 2012 and renewed in 2018.

Porsolt has an in-house ethics programme, which covers animal care and use within the facility.

Additional experimental details about the animal model testing can be found in the Supplementary material.

### Code availability

The R code for computing FM and FAM scores is available at https://figshare.com/projects/Using_common_variants_to_find_drugs_for_common_epilepsies/78330. The code is for non-commercial use only.

### Data availability

The following datasets are available for download from the project’s data repository page (https://figshare.com/projects/Using_common_variants_to_find_drugs_for_common_epilepsies/78330):

- GWAS gene-based and tissue-wide association study (TWAS) datasets used in our analyses.
- Ranked list of the top predicted drugs for each phenotype.
- Our complete set of predictions, listing each drug and its FAM score, for each phenotype.

## Results

### The standard method is inadequate for predicting drugs effective against common epilepsies

In the standard method, drugs are predicted to be efficacious if they modulate the function of proteins that are associated with the disease, according to the GWAS, at a genome-wide level of significance.^21^ For all epilepsy, GE and FE, *SCN1A* is the only gene that both (i) reaches genome-wide level of disease-significance, and (ii) produces a protein that is known to be altered in function by any existing compound. For CAE, JME and HS, there are no genes that both (i) reach genome-wide level of disease-significance and (ii) produce a protein that is known to be altered in function by any existing compound. Predicting candidate compounds for epilepsy based upon their ability to affect the function of sodium channel protein Type 1 subunit alpha (the protein product of *SCN1A*) yields a recall (from amongst all ASMs, the fraction predicted to be effective) of 35% and precision (from amongst all drugs predicted to be effective, the fraction that are ASMs) of 32%, which equates to an F-score (harmonic mean of the precision and recall) of 33%. The standard method of drug prediction produces an unordered and unranked set of candidate drugs, with no metrics for the relative predicted efficacy of the candidate compounds. This precludes method evaluation based upon predicted drug rankings and AUROC, and hampers the selection of the most promising candidate drugs for experimental validation. The same set of ASMs is predicted to be effective for the two divergent phenotypes of GE and FE, even though some seizure types in the former are aggravated by the ASMs that are most effective for the latter. Hence, for different common epilepsies, this method either fails to identify the majority of known effective drugs, or identifies no candidate drugs at all, or identifies potentially aggravating drugs. By extension, applying the standard approach to common epilepsies will yield no or few candidates for repurposing, will not prioritize amongst the candidates, will fail to identify any or most of the efficacious compounds and will potentially identify aggravating drugs.

### Creating and benchmarking a new method for predicting the relative efficacy of drugs against common epilepsies

To predict the relative efficacy of drugs against common epilepsies, we devised the disease-protein FAM score, which is calculated using the method illustrated in Fig. 1.

For benchmarking, we used the FAM score and alternative existing advanced methods to predict drugs for all epilepsy, and compared the methods’ performance. For the identification and prioritization of ASMs, the FAM score achieved AUROC (mean ± standard deviation) of 0.83 ± 0.03 and average percentile of 94, respectively. In comparison, the best performing alternative method achieved AUROC (mean ± standard deviation) of 0.63 ± 0.04 and average percentile of 77. Results of all comparator alternative approaches are shown in Supplementary Table 1.

### Validating the FAM score

Next, we present results of the analyses performed to test the validity of the predictions made using the FAM score.

#### The FAM score for all epilepsy specifically prioritizes the drugs that are effective in people with epilepsy

When drugs are ranked by their FAM score for all epilepsy, drugs used to treat epilepsy are ranked higher than drugs used to treat any other human disease. The median rank of drugs used to treat epilepsy is at least seven percentiles higher than that of drug-sets used to treat other human diseases. Permutation-based *P*-value = 1 × 10^−4^ that ASMs are ranked highest, and so much higher than all other drug-sets used to treat all other human diseases.

#### The FAM score predicts which ASMs are *more* clinically effective, and which ASMs are *less* clinically effective, for each common epilepsy syndrome

Different ASMs are most effective for different syndromes of common epilepsy. Clinical studies and experience show that, for each common epilepsy syndrome, some ASMs can be classified into a more clinically-effective subset and some into a less clinically-effective subset. For each common epilepsy syndrome, the FAM score predicts which ASMs are amongst the more efficacious in clinical practice, and which ASMs are amongst the less efficacious in clinical practice (Table 1). Specifically, for each common epilepsy syndrome, the FAM score (i) distinguishes the more from the less clinically-effective ASMs and (ii) prioritizes the more clinically-effective ASMs higher than the less clinically-effective ASMs (Table 1).

**Table 1:** Performance of the FAM score, measured by the identification and prioritization of AEDs

Epi	Identification of AEDs (AUROC)			Prioritisation of AEDs (average percentile)		*P*
	More effective AEDs from all drugs (mean ± SD)	Less effective AEDs from all drugs (mean ± SD)	More from less effective AEDs	More effective AEDs	Less effective AEDs	
**HS**	0.65 ± 0.13	0.36 ± 0.18	0.87	73	27	8 × 10^–3^
**GE**	0.85 ± 0.04	0.69 ± 0.09	0.71	93	70	<1 × 10^–6^
**JME**	0.88 ± 0.04	0.76 ± 0.08	0.72	96	86	<1 × 10^–6^
**CAE**	0.75 ± 0.05	0.45 ± 0.15	0.79	85	48	2.9 × 10^–5^

Constituents of the ‘More effective AEDs’ and ‘Less effective AEDs’ drug-sets are specific to each phenotype. ‘Less effective AEDs’ comprise the set of less effective, ineffective or aggravating AEDs for that phenotype. AUROC is calculated using drugs’ FAM scores. AUROC for identifying AEDs from all drugs is computed using the technique of random under-sampling, and presented as mean ± standard deviation (see Supplementary methods). Prioritization is calculated using drugs’ ranks, when all drugs have been ranked from highest to lowest predicted effect on the phenotype. Prioritization result shown is the average (median) rank of AEDs, expressed as a percentile; it is equivalent to the percentage of all drugs ranked below the middle-ranked AED (see Supplementary methods). AUROC, area under the receiver operating characteristics; CAE, childhood absence epilepsy; Epi, epilepsy type or syndrome; GE, generalized epilepsy; HS, focal epilepsy with hippocampal sclerosis; JME, juvenile myoclonic epilepsy; *P*, permutation-based *P*-value after Benjamini–Hochberg correction; SD, standard deviation.

In order to predict which ASMs are more clinically-effective and which ASMs are less clinically-effective for a syndrome, the best results are obtained by using the FAM score for that syndrome. To illustrate this, we show that the ASMs that are more effective for CAE are favoured over the ASMs that are less effective for CAE, only when drugs are predicted using the FAM scores for CAE (AUROC: 0.79), and not when drugs are predicted using the FAM scores for all epilepsy, GE, JME, FE or HS (max AUROC: 0.49); permutation-based *P*-value = 1 × 10^−5^ that the AUROC values for CAE and other phenotypes are so contrasting.

For FE, current ASMs are not readily classified into more clinically-effective and less clinically-effective subsets. The FE FAM score identifies and prioritizes ASMs: AUROC (mean ± standard deviation) of 0.85 ± 0.03 and average percentile of 94; the FAM score’ performance is statistically significant (permutation-based *P*-value = 1 × 10^−6^), and superior to that of its constituent scores.

When considering the ability to distinguish more effective ASMs from all drugs and from less effective ASMs, the FAM score outperforms its constituent scores (Supplementary Table 2).

#### The FAM score predicts the observed patterns of relative efficacy of individual clinically-effective ASMs

We tested our predictions against the following observed patterns of relative efficacy of individual clinically-effective ASMs.

#### Valproate is the most effective ASM for GE, whereas carbamazepine is the most effective ASM for FE

It is recognized that the efficacy of valproate for generalized onset seizures is ‘unsurpassed’,^44^ whist for focal onset seizures, ‘no other drug has been shown to be more effective’ than carbamazepine.^45^ In our predictions for GE, valproate is ranked highest of all current ASMs. In our predictions for FE, carbamazepine is ranked highest of all current ASMs. Valproate and carbamazepine are amongst the top two of all drugs in our predictions for GE and FE, respectively; permutation-based *P*-value = 5.6 × 10^−6^ for both valproate and carbamazepine being ranked so highly in our predictions for GE and FE, respectively.

#### The predicted order of efficacy of ASMs for FE matches that seen in the SANAD trials

The SANAD studies are the largest published head-to-head comparison of multiple ASMs for FE, and the largest published randomized controlled trial of ASMs for FE.^46^^,^^47^

Five ASMs were compared in the FE arm of SANAD I: carbamazepine, gabapentin, lamotrigine, oxcarbazepine and topiramate. These drugs’ predicted order of efficacy for FE matches the observed order of efficacy in the SANAD I trial. The finding that these drugs are ranked as highly and in the correct order is unlikely to occur by chance (*P* < 1 × 10^−6^ by permutation).

Carbamazepine and gabapentin are effective ASMs but, in the FE arm of the SANAD I trial, carbamazepine was significantly more efficacious than gabapentin. Carbamazepine and gabapentin are ranked high in our predictions for FE (percentile ranks 100 and 79, respectively), but carbamazepine is ranked significantly higher than gabapentin (permutation-based *P*-value = 1 × 10^−4^ for the ranks of both drugs being as high but as disparate as observed).

The ASMs compared in the FE arm of SANAD II were lamotrigine, levetiracetam and zonisamide. These drugs’ predicted order of efficacy for FE matches the observed order of efficacy in the SANAD II trial. The finding that these drugs are ranked as highly and in the correct order is unlikely to occur by chance (*P* < 1 × 10^−6^ by permutation).

#### The prioritized order of efficacy of ASMs for GE matches that seen in the SANAD I trial

The SANAD studies are the largest published head-to-head comparison of multiple ASMs for GE, and the largest published randomized controlled trial of ASMs for GE.^48^^,^^49^

The ASMs compared in the GE arm of SANAD I were lamotrigine, topiramate and valproate. These drugs’ predicted order of efficacy matches the clinically observed order of efficacy in the SANAD I trial. The finding that these drugs are ranked as highly and in the correct order is unlikely to occur by chance (permutation-based *P*-value = 1 × 10^−5^).

Valproate and lamotrigine are effective ASMs but, in the GE arm of the SANAD I trial, valproate was significantly more efficacious than lamotrigine. Valproate and lamotrigine are ranked high in our predictions for GE (percentile ranks 100 and 81, respectively), but valproate is ranked significantly higher than lamotrigine (permutation-based *P*-value = 3 × 10^−4^ for the ranks of both drugs being as high but as disparate as observed).

The ASMs compared in the GE arm of SANAD II were levetiracetam and valproate. Valproate and levetiracetam are effective ASMs but, in the GE arm of the SANAD II trial, valproate was significantly more efficacious than levetiracetam. Valproate and levetiracetam are ranked high in our predictions for GE (ranks 1 and 15, respectively), but valproate is ranked significantly higher than levetiracetam (permutation-based *P*-value < 1 × 10^−5^ for the ranks of both drugs being as high but as disparate as observed).

#### Topiramate is more effective than lamotrigine for GE, but lamotrigine is more effective than topiramate for FE, in concordance with the SANAD I trial

Lamotrigine and topiramate are the only two ASMs included in both the FE and GE arms of the SANAD I study. In the GE arm of SANAD I, topiramate was more efficacious than lamotrigine, whereas in the FE arm, lamotrigine was more efficacious then topiramate. In our predictions for FE, lamotrigine is ranked higher than topiramate, while for GE, topiramate is ranked higher than lamotrigine. The contrasting ranks of lamotrigine and topiramate for FE and GE are unlikely to occur by chance (permutation-based *P*-value = 1 × 10^−4^).

#### For JME, valproate is most effective

Valproate is thought to be the most efficacious broad-spectrum ASM for JME^50–52^ but this is based on anecdotal data and retrospective analyses. Amongst our predictions for JME, valproate was amongst the highest ranked drugs (percentile rank 98), but not the highest. The highest ranked prediction was primidone. In the longest retrospective cohort study of JME to date, primidone was most effective, with a 5-year terminal remission rate of 73.3, compared to 50% with valproate.^53^

#### For CAE, valproate and ethosuximide are most effective

Valproate and ethosuximide are most effective for CAE; both are similarly effective for CAE.^54^ In our predictions for CAE, valproate is ranked highest of all drugs. Ethosuximide is not ranked highly, but higher than average, amongst our predictions (median percentile rank 58). The *P*-value for the two drugs being ranked so favourably is 5 = 1 × 10^−4^. Ethosuximide is ascribed a particularly low FM score for CAE, which places it in the 20th percentile of predictions for the phenotype. One possible explanation of ethosuximide’s low FM score is that its mechanism of action is poorly understood, as it is not an extensively studied compound. Indeed, ethosuximide is one of the least studied of the current ASMs: there are 343 MEDLINE articles with the word ethosuximide in their title, compared to a mean of ∼1765 for the other current ASMs that are also found in our datasets (as of 2 September 2021; single-sample one-tailed *t*-test *t* = 3.7 and *P*-value = 6.9 × 10^−4^).

### The drug predictions are not driven by individual highly disease-associated proteins

The relative predicted efficacy of drugs does not change significantly after excluding, one at a time, the top 10 most strongly disease-associated proteins that contribute to the FAM score for that epilepsy. The predicted ranks of drugs for each epilepsy remained significantly stable after excluding, one at a time, the top 10 most strongly disease-associated proteins that contribute to the FAM score for that epilepsy. For each epilepsy, FAM scores were re-calculated after excluding, one at a time, the top 10 most strongly disease-associated proteins (Supplementary Table 3) that contribute to the FAM score for that epilepsy. When drug ranks obtained after excluding a protein were compared with the original drug ranks, Kendall’s τ ranged from 0.80 to 0.93, with all corrected *P*-values <1 × 10^−200^. In contrast, comparing the predicted drug rankings for two unrelated epilepsies—CAE and HS—yields a Kendall’s τ of 0.04 (*P* = 0.10).

### Top candidate drugs

Ranked lists of the top drugs predicted to be effective for each phenotype, which are most enriched with the drugs that are known to be (more) effective for the phenotype, are available for download (see Data availability). For each phenotype, the top candidate drugs are significantly (Benjamini–Hochberg *P*-value <0.05) enriched with the ASMs that are (more) effective for the phenotype, except for HS. For HS, there was no significant enrichment of (more) effective ASMs, which may be a reflection of the often drug-resistant nature of HS, or of the lower power of the HS GWAS, or the relatively smaller size of the more effective subset of ASMs for HS, or a combination of these factors.

A manually curated selection of top candidate drugs that could potentially be repurposed for different forms of common epilepsy is shown in the Table 2.

**Table 2:** Manually curated selection of candidate drugs for the phenotypes shown in the table

Epi	Drugs	Evidence of antiseizure efficacy in	Indication	Mode of action
CAE	Clomipramine	Animal models^1^ and humans^2^	Depression	Serotonin–noradrenaline reuptake inhibitor
CAE	Doxepin	Animal models^3,4^	Depression	Tricyclic antidepressant
CAE	Pentoxifylline	Animal models^5^	Peripheral vascular disease	Haemorheological agent, increases leukocyte deformability
CAE	Phenelzine	Animal models^6^	Depression	Monoamine oxidase inhibitor
CAE	Sulindac	Animal models^7^	Pain	Non-steroidal anti-inflammatory
CAE	Tolbutamide	Animal models^8^	Diabetes mellitus	Sulphonylurea
CAE	Tranylcypromine	Animal models^9^	Depression	Monoamine oxidase inhibitor
FE	Chlorzoxazone	Rat hippocampal neurons^10^	Muscle spasms	Calcium and potassium channel inhibitor
FE	Hydrochlorothiazide	Animal models^11, 12^ and human^12^	Hypertension	ACEII antagonist
FE	Thalidomide	Animal models^16-18^	Multiple myeloma	Immunomodulation, unspecified
FE	Zaleplon	Animal models^19^	Insomnia	GABA-BZ agonist
FE	Zolpidem	Animal models^20-22^	Insomnia	GABA-BZ/GABA-A agonist
HS	Amiodarone	Animal models^23^	Arrhythmia	Potassium channel blocker
HS	Clonidine	Animal models^24-44^	Hypertension	Alpha-2 adrenoceptor agonist
HS	Methoxamine	Animal models^45^	Hypotension	Alpha-1 adrenergic receptor agonist
HS	Pergolide	Animal models^46^	Parkinson’s disease	D2 agonist
HS	Thioridazine	Animal models^47^	Psychosis	D1/D2 antagonist
HS	Tizanidine	Animal models^40^	Muscle spasticity	Alpha-2 adrenergic receptor antagonist
JME	Aliskiren	Animal^48, 49^	Hypertension	Renin inhibitor
JME	Baclofen	Animal models^39, 50-76^	Muscle spasticity	GABA-B receptor agonist
JME	Diazoxide	Animal models^77, 78^	Hypoglycaemia	Potassium channel agonist, inhibits insulin release
JME	Icosapent	Animals^79, 80^ and humans^81-85^	Hypertriglyceridaemia	20-carbon omega-3 fatty acid
JME	Iloprost	Animal models^86, 87^	Pulmonary arterial hypertension	Synthetic analogue of prostacyclin PGI2
JME	Nicotinamide	Animal models^94-103^	Pellagra	Water-soluble form of Vitamin B3
JME	Pranlukast	Animal models^104^ and humans^105^	Asthma	Cysteinyl leukotriene receptor-1 antagonist
JME	Riluzole	Animal models^106-109^	Amyotrophic lateral sclerosis	Glutamate antagonist

Candidate drugs for GE, which we tested in an animal model, are listed in Table  3. References, for the evidence cited here, can be found in the Supplementary material. CAE, childhood absence epilepsy; Epi, epilepsy type or syndrome; HS, focal epilepsy with hippocampal sclerosis; JME, juvenile myoclonic epilepsy.

### Predicted drugs have a significant dose-dependent effect on seizures in an animal model

After excluding drugs that are toxic or otherwise unsuitable, the top five predicted drugs for GE were tested in a mouse model with a complex genetic seizure disorder that manifests as audiogenic generalized seizures. Each of the drugs had a significant dose-dependent effect on tonic and clonic convulsions (Table 3). Whilst four of the drugs had a significant dose-dependent *anti*-convulsant effect, one of the compounds (betahistine) had a significant dose-dependent *pro*-convulsant effect.

**Table 3:** Results from testing compounds in a genetic model of generalised seizures: the DBA/2 mouse model of audiogenic seizures

Drug	Latency (s) to convulsions (mean±s.e.m)	*P*
Vehicle (i.p.)	10.9 ± 2.6	–
Orphenadrine (12.5 mg/kg i.p.)	40.0 ± 5.6	6.10 × 10^–5^
Orphenadrine (25 mg/kg i.p.)	53.4 ± 3.7	5.40 × 10^–7^
Orphenadrine (50 mg/kg i.p.)	60.0 ± 0.0	4.14 × 10^–7^
Dyclonine (5 mg/kg i.p.)	31.5 ± 6.2	1.77 × 10^–2^
Dyclonine (10 mg/kg i.p.)	44.7 ± 5.4	2.16 × 10^–4^
Dyclonine (20 mg/kg i.p.)	57.7 ± 2.4	4.14 × 10^–7^
Trimeprazine (2.5 mg/kg i.p.)	11.0 ± 20.6	6.52 × 10^–1^
Trimeprazine (5 mg/kg i.p.)	18.1 ± 4.1	1.77 × 10^–2^
Trimeprazine (10 mg/kg i.p.)	44.5 ± 5.3	4.06 × 10^–6^
Acamprosate (125 mg/kg i.p.)	8.7 ± 0.4	6.40 × 10^–1^
Acamprosate (250 mg/kg i.p.)	9.2 ± 0.2	4.56 × 10^–1^
Acamprosate (500 mg/kg i.p.)	14.3 ± 2.5	1.20 × 10^–2^
Betahistine (75 mg/kg i.p.)	9.1 ± 0.5	4.53 × 10^–1^
Betahistine (150 mg/kg i.p.)	6.9 ± 0.4	2.83 × 10^–2^
Betahistine (300 mg/kg i.p.)	5.3 ± 0.3	4.48 × 10^–5^
Valproate (180 mg/kg i.p.)	57.7 ± 1.4	4.89 × 10^–7^

After activation of a bell, latency to the occurrence of tonic convulsions and clonic convulsions was measured. *P*, Benjamini–Hochberg-corrected *P*-value from two-sided Mann–Whitney *U* test; s.e.m, standard error of the mean.

## Discussion

We present the relative predicted efficacy of drugs against each of the main types and syndromes of common epilepsy. This dataset is a novel and valuable resource for selecting the best candidate drug(s) to repurpose for any of the main types and syndromes of common epilepsy. Of course, our predicted candidate drugs require further animal model and/or human clinical trial evidence before being considered for deployment in clinical practice.

To generate our predictions, we created a novel method. Our method possesses several strengths that are lacking in previously published approaches. Common epilepsies, like other complex diseases, develop when many different proteins display abnormal activity due to pathological changes in their abundance or function.^55^ Our method prioritizes drugs according to their relative ability to modulate changes in both the abundance and the function of disease-proteins. Furthermore, drugs are prioritized on the basis of their ability to correct disease-protein abnormalities that are found in people with the disease, rather than in animal models, and that are not consequential to or compensatory for the disease, as they are driven by germline variations. We use genetic variation data specific to each form of common epilepsy, to make drug predictions specific to that form of common epilepsy. The ASMs that are more clinically-effective for a syndrome and the ASMs that are less clinically-effective for a syndrome are predicted more effective and less effective, respectively, for that syndrome only, but not for any other epilepsy type or syndrome—this suggests that our predictions are not systemically biased in favour of a particular set or type of drugs. The methodology is based upon a polygenic model of disease and a multi-targeted approach to treatment, which are desirable for complex diseases. We utilize conventional canonical low-throughput single-target functional drug activity data, and high-throughput genome-wide transcriptomic drug activity data, so that prioritization of drugs is informed by their on-target and off-target effects, and by their affinities for individual proteins and effects upon genome-wide gene expression. The directionality of drugs’ effects on protein activity also helps inform drug prioritization. Rather than dichotomous categorization of compounds into drugs that are predicted to be effective or ineffective, our method ranks drugs individually according to relative predicted efficacy, which aids candidate selection for *in vivo* validation and for development.

Our method produces accurate drug predictions for epilepsy syndromes even if their GWAS results include few genome-wide significant loci. Even excluding the most strongly disease-associated proteins does not significantly change the relative efficacy of drugs predicted by our method (as we show in the Results, under subheading ‘The drug predictions are not driven by individual highly disease-associated proteins’). This is because our method is not reliant on individual highly disease-associated proteins. Instead, our method leverages the gene-set analysis approach, where each gene-set is the set of genes affected by each drug. The disease association of all the genes in a gene-set, even those below the genome-wide significant threshold, is combined; the gene-sets that are more disease-associated overall are more biologically relevant. The gene-set approach is a long-established and widely-used method in all areas of genomic analysis,^56^ including post-GWAS analysis generally^57^ and GWAS-based drug repurposing analysis specifically.^27^^,^^28^ Utilizing the full distribution of all genetic associations for gene-set analysis is a validated, established and accepted approach, which has been implemented in numerous widely-used post-GWAS analysis tools, for example FUMA,^58^ MAGMA,^58^ MAGENTA,^59^ INRICH^60^ and DEPICT,^61^ each of which has been employed in a multitude of published GWAS-based studies.

Alongside these strengths, our method has some limitations, discussed below.

Our drug prediction method, like all previously published genetics- or genomics-based drug prediction methods, predicts the efficacy of drugs for a disease. However, the most efficacious drug for a disease in not always the most appropriate drug for every individual with the disease. Important factors to consider when choosing a drug for an individual include the potential of undesirable interactions with other medications and the possible side-effects. Our method, like all previously published genetics- or genomics-based drug prediction methods, does not predict drugs’ interactions with other medications and side-effects. Indeed, the success of an ASM is determined as much by its tolerability as by its efficacy.^59–62^ As the drugs we have predicted for epilepsies are already being used for other diseases, their side-effect profiles are known. This allows researchers to select for further development those candidate compounds whose side-effects are less deleterious or even desirable.

Our method predicts drugs effective for a disease from the proteins underlying the disease, after identifying the proteins underlying the disease from the common genetic variations associated with the disease. However, some proteins become dysfunctional or dysregulated not because of common genetic variations, but because of rare genetic variations, or copy number variations, or abnormalities of epigenetic, post-transcriptional or post-translational mechanisms, or because of environmental insults. Such protein changes do not inform our predictions, which could affect their accuracy, commensurate with the contribution of those proteins to the causal mechanism underlying an epilepsy and/or to the mechanism of action of a drug. We are not aware of any existing drug prediction methods which take into account the multiple potential pathogenic factors that influence proteins; the development of such methods might lead to improved accuracy of drug predictions.

Our analysis uses data from a GWAS that employed imputation to improve genomic coverage. The GWAS gene-level data used in this analysis offers coverage of genes across the genome, and it is corrected for the lengths and single nucleotide polymorphism-densities of genes. However, if a gene is not (adequately) covered by the genotyping array and the imputation, but the gene is of importance in epilepsy and affected by drug(s), the accuracy of our drug predictions could be adversely affected. Hence, improved coverage of future epilepsy GWAS analyses could help to improve the accuracy of drug predictions.

Our drug predictions are based upon two scores: the FM and AC scores. The FM score relies upon knowledge of the proteins changed in function by drugs. At present, knowledge of the proteins that are changed in function by each drug is incomplete, and it is more incomplete for some drugs than for others. The more incomplete the knowledge of the proteins changed in function by a drug, the more likely it is that the drug’s FM score will be underestimated. By extension, the FM score is more likely to be underestimated for drugs that are less studied, as their modes of action are less analysed and, hence, knowledge of the proteins changed in function by them is less complete. This may explain the relatively low FM sore and, hence, FAM score and ranking for ethosuximide. The AC score is free of this limitation, as the AC score is based upon profiles of drug-induced transcriptomic changes assayed by using the same standardized pipeline for each drug. With over 44 000 compounds already analysed on this platform (http://lincsportal.ccs.miami.edu/SmallMolecules/catalog; accessed on 1 February 2021), transcriptomic profiles are available for the vast majority of drugs of interest. However, a small number of interesting drugs (for example, brivaracetam and cenobamate) have not been assayed, which means that an AC score and, hence, a FAM score cannot be calculated for them. The platform and pipeline used for generating drugs’ transcriptomic profiles are in the public domain, and have been used by researchers to generate profiles for any compounds of interest not already found in the database, albeit for industrial-scale projects.^62^ In addition, there is active ongoing development of computational methods for using knowledge of drugs’ structures to predict the proteins that they change in function and/or abundance,^63^^,^^64^ which is another potential future strategy for predicting the relative efficacy of compounds whose molecular effects are still unknown.

It is noted that the FM score does not predict the ‘directionality’ of drugs’ effects (that is, beneficial or harmful) on disease-protein function. Therefore, drugs predicted by the FM score to affect a phenotype may be alleviating or aggravating for the phenotype. This is a recognized limitation of methods that use data for the ability of drugs to alter the function of genetically-associated disease-proteins in order to predict drugs that can affect the disease,^16^^,^^17^^,^^65^ as the direction of change in protein activity occurring in the disease is unknown. On the other hand, the AC score does predict the ‘directionality’ of drugs’ effects (that is, beneficial or harmful) on individual disease-proteins and, thereby, the overall ‘directionality’ of drugs’ effects (that is, beneficial or harmful) on the disease. The AC score takes into account the magnitude and direction of change in proteins’ abundance underlying disease, and the magnitude and direction of change in proteins’ abundance caused by drugs. Thereby, the AC score proposes to predict the drugs with a beneficial effect on disease-protein abundance and clinical phenotype. Hence, inclusion of the AC score, with the FM score, in our final FAM score, is expected to help mitigate the risk of deleterious compounds with high FM scores being included in our candidate drugs. Still, it is possible that some aggravating drugs are included in our candidate compounds. Hence, experimental validation of candidate drugs is essential before clinical use, as with all *in silico* drug prediction methods. We tested five of our candidate compounds in a rodent model: all five compounds had a significant dose-dependent effect on seizures. Interestingly, one of the candidate compounds (betahistine) had a significant dose-dependent pro-convulsant effect in the animal model. This finding could be explained by the possibility that some of our predicted compounds are aggravating, as discussed. However, it is also possible that the pro-convulsant effect of betahistine in our study is a reflection of species- or model-specific behaviour. Indeed, a recent study (published after our animal experiments had ended) showed that betahistine has a significant antiepileptogenic and anticonvulsant effect on pentylenetetrazole-induced generalized seizures in a different mouse strain.^66^

Whilst acknowledging these limitations and some aberrant predictions, we note that our method outperforms alternative methods for predicting drugs that have efficacy against common epilepsies in clinical studies and experience. Our method also predicts which ASMs are amongst the more efficacious in clinical practice, and which ASMs are amongst the less efficacious in clinical practice, for each of the main syndromes of common epilepsy, and it predicts the distinct order of efficacy of individual ASMs in clinical trials of different common epilepsies. This aspect is key to the clinical translation of drug predictions for common epilepsies, but is missing from previously published studies that have predicted drugs for epilepsy.^13–17^

In this study, we have used the tissue-wide association study method to identify the protein abundance changes underlying disease. A closely-related alternative method is to use Mendelian randomization. In future studies, both methods could be compared and/or combined in order to determine if this improves the drug predictions. Mendelian randomization is discussed at greater length in the Supplementary material.

As our method uses GWAS data, it cannot be applied to monogenic diseases. It is conceivable that this method could be adapted to make it applicable to monogenic diseases, and we plan to explore this possibility in a future study dedicated to this objective.

We have used results from the latest epilepsy GWAS mega-analysis, which includes previously published and unpublished epilepsy GWAS analyses, making it the largest epilepsy GWAS to date.^11^ However, compared to other common neurological diseases, even the largest epilepsy GWAS had a modest sample size, with 15 212 cases and 29 677 controls, and produced a modest number of discoveries, with 16 loci identified. The latest schizophrenia GWAS, for example, included 36 989 cases and 113 075 controls, resulting in the identification of 108 risk loci.^67^ It is hoped that expanded cohort sizes of future epilepsy GWAS analyses will increase power and improve drug predictions. In this analysis, we predicted drugs for the main epilepsy syndromes that had risk loci identified in the latest epilepsy GWAS. It is hoped that future epilepsy GWAS will be large enough to report results for additional epilepsy syndromes, and drugs can be predicted for them using the method presented here. Finally, it is likely that our method can be applied to the GWAS results of other common complex phenotypes.

## Supplementary material

Supplementary material is available at *Brain Communications* online.

## Supplementary Material

fcab287_Supplementary_Data

## Appendix I

The International League Against Epilepsy Consortium on Complex Epilepsies author names are shown below. Members are listed in alphabetical order. Author affiliations can be found in the Supplementary material.

Bassel Abou-Khalil, Pauls Auce, Andreja Avbersek, Melanie Bahlo, David J. Balding, Thomas Bast, Larry Baum, Albert J. Becker, Felicitas Becker Bianca Berghuis, Samuel F. Berkovic, Katja E. Boysen, Jonathan P. Bradfield, Lawrence C. Brody, Russell J. Buono, Ellen Campbell, Gregory D. Cascino, Claudia B. Catarino, Gianpiero L. Cavalleri, Stacey S. Cherny, Krishna Chinthapalli, Alison J. Coffey, Alastair Compston, Antonietta Coppola, Patrick Cossette, John J. Craig, Gerrit-Jan de Haan, Peter De Jonghe, Carolien G. F. de Kovel, Norman Delanty, Chantal Depondt, Orrin Devinsky, Dennis J. Dlugos, Colin P. Doherty, Christian E. Elger, Johan G. Eriksson, Thomas N. Ferraro, Martha Feucht, Ben Francis, Andre Franke, Jacqueline A. French, Saskia Freytag, Verena Gaus, Eric B. Geller, Christian Gieger, Tracy Glauser, Simon Glynn, David B. Goldstein, Hongsheng Gui, Youling Guo, Kevin F. Haas, Hakon Hakonarson, Kerstin Hallmann, Sheryl Haut, Erin L. Heinzen, Ingo Helbig, Christian Hengsbach, Helle Hjalgrim, Michele Iacomino, Andrés Ingason, Jennifer Jamnadas-Khoda, Michael R. Johnson, Reetta Kälviäinen, Anne-Mari Kantanen, Dalia Kasperavičiūte, Dorothee Kasteleijn-Nolst Trenite, Heidi E. Kirsch, Robert C. Knowlton, Bobby P. C. Koeleman, Roland Krause, Martin Krenn, Wolfram S. Kunz, Ruben Kuzniecky, Patrick Kwan, Dennis Lal, Yu-Lung Lau, Holger Lerche, Costin Leu, Wolfgang Lieb, Dick Lindhout, Warren D. Lo, Iscia Lopes-Cendes, Daniel H. Lowenstein, Alberto Malovini, Anthony G. Marson, Thomas Mayer, Mark McCormack, James L. Mills, Nasir Mirza, Martina Moerzinger, Rikke S. Møller, Anne M. Molloy, Hiltrud Muhle, Mark Newton, Ping-Wing Ng, Markus M. Nöthen, Peter Nürnberg, Terence J. O’Brien, Karen L. Oliver, Aarno Palotie, Faith Pangilinan, Sarah Peter, Slavé Petrovski, Annapurna Poduri, Michael Privitera, Rodney Radtke, Sarah Rau, Philipp S. Reif, Eva M. Reinthaler, Felix Rosenow, Josemir W. Sander, Thomas Sander, Theresa Scattergood, Steven C. Schachter, Christoph J. Schankin, Ingrid E. Scheffer, Bettina Schmitz, Susanne Schoch, Pak C. Sham, Jerry J. Shih, Graeme J. Sills, Sanjay M. Sisodiya, Lisa Slattery, Alexander Smith, David F. Smith, Michael C. Smith, Philip E. Smith, Anja C. M. Sonsma, Doug Speed, Michael R. Sperling, Bernhard J. Steinhoff, Ulrich Stephani, Remi Stevelink, Konstantin Strauch, Pasquale Striano, Hans Stroink, Rainer Surges, K. Meng Tan, Liu Lin Thio, G. Neil Thomas, Marian Todaro, Rossana Tozzi, Maria S. Vari, Eileen P. G. Vining, Frank Visscher, Sarah von Spiczak, Nicole M. Walley, Yvonne G. Weber, Zhi Wei, Judith Weisenberg, Christopher D. Whelan, Peter Widdess-Walsh, Markus Wolff, Stefan Wolking, Wanling Yang, Federico Zara and Fritz Zimprich.

## Abbreviations

AC score =: abundance correction score
ASM =: antiseizure medication
CAE =: childhood absence epilepsy
FAM score =: function and abundance modulation score
FE =: focal epilepsy
FM score =: function modulation score
GE =: generalized epilepsy
GWAS =: Genome-Wide Association Study
HS =: hippocampal sclerosis
ILAE =: International League against Epilepsy
JME =: juvenile myoclonic epilepsy
ROC =: area under receiver operated characteristics curve
