# Supplementary material for: Using common genetic variants to find drugs for common epilepsies
Source: Brain Commun. 2021 Dec 4;3(4):fcab287. doi: 10.1093/braincomms/fcab287 (PMC8710935; doi:10.1093/braincomms/fcab287)
Supplement: fcab287_Supplementary_Data [file fcab287_supplementary_data.docx]

**Supplementary Material**

Contents

[Supplementary Methods 3](#_Toc66329935)

[Data collection 3](#_Toc66329936)

[Protein activity changes found in disease and produced by drugs 3](#_Toc66329937)

[A complete set of all ASMs 3](#_Toc66329938)

[Specific subsets of ASMs that are more effective or less effective for specific common epilepsy syndromes 4](#_Toc66329939)

[Metrics to compare methods for predicting effective drug-sets 5](#_Toc66329940)

[Developing and utilising scores for predicting effective drugs 6](#_Toc66329941)

[Developing the Function Modulation (FM) score 6](#_Toc66329942)

[Comparison with existing alternative enhanced methods 10](#_Toc66329943)

[Adoption of the ‘disease-association of all proteins affected and the magnitude of effect’ method 11](#_Toc66329944)

[Utilising the Abundance Correction (AC) score 12](#_Toc66329945)

[Developing the Function and Abundance Modulation (FAM) score 16](#_Toc66329946)

[Comparison with existing alternative enhanced methods 16](#_Toc66329947)

[Validation of the FAM score. 17](#_Toc66329948)

[Are the drug predictions driven by individual highly disease-associated proteins? 19](#_Toc66329949)

[Filtered lists of promising candidate drugs 19](#_Toc66329950)

[Animal model testing 20](#_Toc66329951)

[Collating the top proteins that contribute most to the FAM scores 23](#_Toc66329952)

[Supplementary Results 24](#_Toc66329953)

[Our sequential methodological enhancements, sequentially improve performance 24](#_Toc66329954)

[Our methods outperform existing alternative enhanced methods 24](#_Toc66329955)

[Utilising more drug activity data further improves the performance of our best method 24](#_Toc66329956)

[Integrating the FM and AC scores leads to improved performance 25](#_Toc66329957)

[Determining whether the drug predictions are dominated by the FM or AC sore 25](#_Toc66329958)

[References for Table 2 26](#_Toc66329959)

[Supplementary Tables and Figures 38](#_Toc66329960)

[The International League Against Epilepsy Consortium on Complex Epilepsies author names 55](#_Toc66329961)

[Supplementary References 63](#_Toc66329962)

# Supplementary Methods

# Data collection

## Protein activity changes found in disease and produced by drugs

Summary statistics from the latest and largest GWAS of AE, FE, HS, GE, JME and CAE were downloaded (<http://www.epigad.org/gwas_ilae2018_16loci.html>; accessed 01/03/2019). The summary statistics were converted to gene-based p-values in FUMA (<https://fuma.ctglab.nl/>) (Watanabe *et al.*, 2017) using default settings. The gene-based p-values were used as proxies for the relative association of the corresponding proteins with the phenotype. Changes in the abundance of gene transcripts (and, by proxy, the corresponding proteins) were calculated from GWAS summary statistics using Fusion (see below).

Data for proteins changed in function by drugs was downloaded from the ChEMBL database (<https://www.ebi.ac.uk/chembl/g/#browse/mechanisms_of_action>; accessed 12/08/2019). In order to reduce the computational burden, this list was limited to prescribable drugs (Brown and Patel, 2017b; Santos *et al.*, 2017), including all drugs used effectively (now or in the past) to treat epilepsy (see below). Drug-target affinity data, which has been collated from multiple sources and made available within the Drug Target Commons database, was downloaded (https://drugtargetcommons.fimm.fi; accessed 15/08/2019). Data for proteins changed in abundance by drugs was obtained from the Library of Integrated Network-Based Cellular Signatures (LINCS) Program (Subramanian *et al.*, 2017), as provided in the Combination Connectivity Mapping (version 1.4.0) bioconductor package (<https://www.bioconductor.org/packages/release/bioc/html/ccmap.html>) (Pickering, 2017).

## A complete set of all ASMs

To enable optimal objective assessment of the methodologies for predicting ASMs, we created a comprehensive list of effective drugs used currently or previously for the treatment of epilepsy or seizures in people. In this set, we included all drugs licensed for the treatment of seizures or epilepsy according to the British National Formulary (<https://bnf.nice.org.uk/>; accessed on the 15^th^ of January, 2019), and all antiepileptic drugs listed in the World Health Organization (WHO) Anatomical Therapeutic Chemical (ATC) Classification System (<https://www.whocc.no/atc_ddd_index/>; accessed on the 15^th^ of January, 2019) category N03A, and other drugs effectively used, now or in the past, for the treatment of epilepsy or seizures, including status epilepticus, according to published literature (Shorvon *et al.*; Shorvon, 2009a, b; Brodie, 2010). The set of all ASMs included 28 compounds also found in our collated drug datasets: acetazolamide, carbamazepine, chlordiazepoxide, diazepam, ethosuximide, ethotoin, felbamate, gabapentin, lamotrigine, levetiracetam, lidocaine, lorazepam, mephenytoin, midazolam, oxcarbazepine, pentobarbital, phensuximide, phenytoin, piracetam, primidone, propofol, stiripentol, tiagabine, topiramate, trimethadione, valproic acid, vigabatrin and zonisamide.

## Specific subsets of ASMs that are more effective or less effective for specific common epilepsy syndromes

Clinical studies and experience show that, for each common epilepsy syndrome, some ASMs can be classified into a more clinically-effective subset and some into a less clinically-effective subset. We aimed to determine if our methodology correctly predicts which ASMs are more clinically-effective, and which ASMs are less clinically-effective, for each common epilepsy syndrome. We collated the more clinically-effective and less clinically-effective ASM subsets that are specific to each phenotype. We included ASMs that are less effective, ineffective or aggravating for a phenotype, in its less clinically-effective subset. We created ASM subsets that were as complete and comprehensive as possible, based upon currently available objective evidence. For each phenotype, some ASMs cannot be definitively classified into the more or the less clinically-effective subset, because of the lack of relevant studies; these indeterminate ASMs were not included in either subset. The common epilepsy syndromes and the specific subsets that are more clinically-effective or less clinically-effective for them are summarised below:

- GE and JME
  - More effective subset (‘broad-spectrum’ ASMs): ASMs that are effective for all types of focal-onset and generalised-onset seizures (Supplementary Table 4) (Shorvon *et al.*)
  - Less effective subset (‘narrow-spectrum’ ASMs): ASMs that are effective for focal-onset and some types of generalised-onset seizures, but are ineffective or aggravating for other types of generalised-onset seizures (Supplementary Table 4) (Shorvon *et al.*)
- CAE
  - More effective subset: ASMs effective for CAE or typical absences (Supplementary Table 5)
  - Less effective subset: ASMs ineffective or aggravating for CAE or typical absences (Supplementary Table 5).
- HS: ASMs are often ineffective against HS. There is no clinical trial data to indicate if any subset of ASMs is relatively more effective for HS. The largest retrospective database study of the comparative effectiveness of ASMs in HS analysed ten drugs. We dichotomised this set of ASMs into the more effective half (carbamazepine, lamotrigine, levetiracetam, oxcarbazepine, valproicacid), and the less effective half (clobazam, gabapentin, pregabalin, topiramate, vigabatrin), according to reported response rates (12-month seizure freedom rates). The mean (±standard deviation) response rate for the more effective ASM subset was 8.34% (±2.04%), whilst that for the less effective ASM subset was 2.84% (±1.6%); one-tailed t-test t-value=4.70 and p-value=7.69e-04.

# Metrics to compare methods for predicting effective drug-sets

Two measures were used to compare how well different methods predict effective drug-sets.

1. Identification of effective drugs: we use area under receiver operated characteristics curve (AUROC) analysis to determine the accuracy with which the scores that are assigned to each of the drugs discriminate ASMs from other drugs, or discriminate more from less clinically-effective subsets of ASMs.
2. Prioritisation of effective drugs: amongst all of the method’s drug predictions for a phenotype, we determine the average rank of ASMs, or compare the average rank of more clinically-effective and less clinically-effective subsets of ASMs for the phenotype (if known), or compare the rank of individual ASMs whose relative clinical efficacy for the phenotype is known.

AUROC was calculated using the package PRROC (version 1.3.1) (Grau *et al.*, 2015) in R (version 3.4.3). In assessing the identification of ASMs from amongst all other drugs, there is a marked class imbalance, because a very small fraction of all drugs are ASMs. To correct for this imbalance, we employ the standard technique of random under-sampling, which is commonly used in published studies (Cao *et al.*, 2012; Sun *et al.*, 2012; Guney *et al.*, 2016; Mousavian *et al.*, 2016; Rayhan *et al.*, 2017; Cheng *et al.*, 2019; Ivanov *et al.*, 2019; Kim *et al.*, 2019; Rayhan *et al.*, 2019). AUROC is calculated using the set of ASMs and a randomly selected set of other drugs equal in number to the ASMs. This process is repeated 1000 times, and mean (± standard deviation) AUROC is calculated. When differentiating more from less effective ASMs, class imbalance is not an issue and, hence, random under-sampling is not employed.

Prioritisation is calculated using drugs’ ranks, when all drugs have been ranked from highest to lowest predicted effect on the phenotype. To ease conceptualisation and interpretation of results, we convert ranks to percentile ranks. For example, a drug with a percentile rank of 90 is ranked higher/better than 90% of all drugs. Like numerous published studies (Bornigen *et al.*, 2012; Fang and Gough, 2013; Tarca *et al.*, 2013; Masino *et al.*, 2014; Chen *et al.*, 2015; Cai *et al.*, 2016; Chen *et al.*, 2016; Chen and Xu, 2016; Zhai *et al.*, 2016; Simillion *et al.*, 2017; Donner *et al.*, 2018; Mansoori *et al.*, 2019) we use the median in order to compute the average of ranks, as it is less liable to skewing by outliers.

# Developing and utilising scores for predicting effective drugs

## Developing the Function Modulation (FM) score

In the standard approach, compounds are predicted to be efficacious if they modulate the function of proteins that are associated with the disease, according to the GWAS, at a genome-wide level of significance (Sanseau *et al.*, 2012)

Starting from the standard approach, we create three consecutive methodological enhancements (Supplementary Table 6), each of which builds progressively upon the last, by incorporating additional features from the data that describes the relationship between drugs-proteins-disease, to generate a superior ‘raw’ score for each drug at each step. The unique premise and method underlying each enhancement is detailed below.

*First enhancement: A drug is more likely to affect a disease if it affects the function of a protein more strongly associated with the disease—the ‘most disease-associated protein affected’ method*

A simple enhancement, to the standard method, is based on the premise that a drug that alters the function of a more disease-associated protein is more likely to be effective than a drug that alters the function of a less disease-associated protein. The approach, in principle, is to sequentially relax the GWAS gene-based p-value threshold, allowing the next most strongly disease-associated protein to pass the threshold at each step, and to ascribe a sequentially lower score to the compounds that change the function of a sequentially less disease-associated protein. In practice, this method is executed as follows: each drug is ascribed a raw score equal to the multiplicative inverse of the GWAS gene-based p-value of the most disease-associated protein it alters in function.

Knowledge about the specific set of proteins changed in function by each drug is still incomplete. Current databases’ lists of proteins changed in function by drugs is less complete for some drugs than for other drugs. Drugs known to alter the function of more proteins are likely to have more highly disease-associated proteins amongst them and, hence, to acquire a higher raw score. Hence, if drugs are compared using their raw scores, then drugs that are known to affect the function of more proteins are advantaged, even if their raw score is no greater than that expected by chance, for the number of proteins affected. Therefore, we compare drugs using an adjusted score that controls for the different number of proteins affected by each drug. The adjusted score is equivalent to the likelihood of observing by chance raw scores equal to (or greater than) the magnitude seen, for the number of proteins known to be affected by each drug. Practically, this is the frequency with which random scores (from a null distribution generated by permutation of the GWAS gene-based p-values) equal or exceed the raw score. To generate the null distribution, GWAS gene-based p-values were permuted 10^6^ times.

By permutation of adjusted scores, we determined if this method is significantly better than chance observation at prioritising and discriminating known ASMs.

*Second enhancement: A drug is more likely to affect a disease if it affects the function of more proteins more strongly associated with the disease—the ‘disease-association of all proteins affected’ method*

An enhancement to the preceding method is based upon the premise that a drug’s effects on multiple proteins produce an additive effect on the clinical phenotype (Bolognesi, 2013; Anighoro *et al.*, 2014; Zhang *et al.*, 2016; Proschak *et al.*, 2019). Therefore, drugs are more likely to be effective if they affect the function of more proteins more strongly associated with the disease.

A raw score for each drug is obtained by summing the multiplicative inverse of the GWAS gene-based p-value of each protein it changes in function. Knowledge about the specific set of proteins changed in function by each drug is still incomplete (Peterson, 2008; Keiser *et al.*, 2009; Nobeli *et al.*, 2009). Current databases’ lists of proteins changed in function by drugs is less complete for some drugs than for other drugs. If drugs are compared using their raw scores, then drugs that are known to affect the function of more proteins are advantaged, even if their raw score is no greater than that expected by chance, for the number of proteins affected. Therefore, we compare drugs using an adjusted score that controls for the different number of proteins affected by each drug. The adjusted score is equivalent to the likelihood of observing by chance raw scores equal to (or greater than) the magnitude seen, for the number of proteins known to be affected by each drug. Practically, this is the frequency with which random scores (from a null distribution generated by permutation of the GWAS gene-based p-values) equal or exceed the raw score. To generate the null distribution, GWAS gene-based p-values were permuted 10^6^ times. This ‘adjusted score’ is expected to prioritise compounds that affect the function of more proteins more strongly associated with the disease.

By permutation of adjusted scores, we determined if this method is significantly better than chance observation at prioritising and discriminating known ASMs.

*Third enhancement: A drug is more likely to affect a disease if it has a stronger effect on the function of more proteins more strongly associated with the disease—the ‘disease-association of all proteins affected and the magnitude of effect’ method*

An enhancement on the preceding method is based upon the following premise: drugs are more likely to be effective if they affect the function of proteins that are more disease-associated and if they do so more potently. The multiplicative inverse of the GWAS gene-based p-value of each protein that a drug changes in function is boosted in proportion to the affinity of the drug for the protein, up to twice the original inverse p-value (see below). Capping the boost in the inverse gene-based p-value at 100% of the original inverse gene-based p-value, and calculating it as a multiplicative increase in the original inverse gene-based p-value, protects the primacy of disease-association within the scoring system. This implementation limits the possibility that scores for drugs with high affinities for weakly disease-associated proteins will exceed scores for drugs that change the function of highly disease-associated proteins. The boosted inverse p-values for all proteins that a drug changes in function are summed, giving the raw score for the drug. If no affinity data is available for a drug-protein pair, the protein’s inverse p-value is used unchanged in the drug’s raw score.

Knowledge about the specific set of proteins changed in function by each drug is still incomplete (Peterson, 2008; Keiser *et al.*, 2009; Nobeli *et al.*, 2009). Current databases’ lists of proteins changed in function by drugs is less complete for some drugs than for other drugs. If drugs are compared using their raw scores, then drugs that are known to affect the function of more proteins are advantaged, even if their raw score is no greater than that expected by chance, for the number of proteins affected. Similarly, knowledge of drugs’ affinities for different proteins is still incomplete: drug-protein affinities are known for some, but not all, drug-protein pairs. Current knowledge of drugs’ affinities for different proteins is less complete for some drugs than for other drugs. If drugs are compared using their raw scores, then drugs with known affinity values for more proteins are advantaged, even if their raw score is no greater than that expected by chance, for the relevant number of proteins. Therefore, we compare drugs using an adjusted score that controls for the different number of proteins affected by each drug, and the different number of proteins for which each drug has known affinity values. The adjusted score is equivalent to the likelihood of observing by chance raw scores equal to (or greater than) the magnitude seen, for the number of proteins known to be affected by each drug and the number of proteins for which each drug has known affinity values. Practically, this is the frequency with which random scores (from a null distribution generated by permutation of the GWAS gene-based p-values and drug affinities) equal or exceed the raw scores. To generate the null distribution, GWAS gene-based p-values were permuted 10^6^ times. This ‘adjusted score’ is expected to prioritise compounds that have a stronger effect on the function of more proteins more strongly associated with the disease.

By permutation of adjusted scores, we determined if this method is significantly better than chance observation at prioritising and discriminating known ASMs.

For use in the scoring, drug-protein affinities were extracted and rescaled as follows. Drug affinities (in nM) were extracted from Drug Target Commons (Tanoli *et al.*, 2018) (<https://drugtargetcommons.fimm.fi/>; accessed 15/08/2019). Chemical identifiers were converted to drug names and protein identifiers were converted to HGNC-approved gene symbols. In order to reduce the computational burden, drug-protein affinity values were limited to prescribable drugs (Brown and Patel, 2017b; Santos *et al.*, 2017), including all drugs used effectively (now or in the past) to treat epilepsy (see above). Affinity values of 10,000 nM, or stronger, were taken as evidence of affinity, in keeping with previous databases and studies (Gilson *et al.*, 2016; Guney *et al.*, 2016; Cheng *et al.*, 2018; Cheng *et al.*, 2019), and weaker affinities were discarded, in order to exclude potentially spurious data. For drug-protein pairs with multiple affinity values, the minimum affinity value was used. Hence, the final dataset comprised one affinity value per drug-protein pair. The affinity values were then rescaled from 1 (weakest affinity) to 2 (strongest affinity).

### Comparison with existing alternative enhanced methods

*Identifying drugs that affect the function of proteins in the disease-proteins’ interaction network*

In order to expand the number of potentially-effective candidate compounds, some studies identify the drugs that target genome-wide significant disease-proteins and, in addition, the drugs that target the proteins interacting with genome-wide significant disease-proteins (Okada *et al.*, 2014; Wain *et al.*, 2017; Wang *et al.*, 2018). An efficient and validated tool for executing this analysis is the GUILDify v2.0 Web Server (<http://aleph.upf.edu/guildify2/>; accessed 16/08/2019). Using a set of ‘seed’ proteins provided by the user, GUILDify v2.0 constructs a network of proteins that interact with the seeds, and lists drugs that target the seed and the interacting proteins. Guildify was run using default settings (Species: Homo sapiens; Tissue: all; Network: BIANCA; NetScore repetitions 3 and iterations 2). The manuscript of the epilepsy GWAS includes a set of ‘prioritized biological epilepsy genes’ for the AE phenotype (International League Against Epilepsy Consortium on Complex Epilepsies, 2018); these genes were chosen as seeds for generating the protein-protein interaction network in Guildify v2.0, as they are more numerous than the genes that pass the GWAS gene-based Benjamini–Hochberg-corrected p-value <0.05 threshold. The list of drugs that target the seed and the interacting proteins was downloaded from the Guildify v2.0 website. This list of drugs was used to calculate the precision, recall and F-score for predicting ASMs.

*Gene-set t-test method of So, et al.*

We compared our results with those from a recently-published method (So *et al.*, 2018) of using GWAS data to identify effective drugs. In this method, GWAS gene-based p-values are converted to z-statistics. Then, for each drug, a single-sided two-sample t-test is used to determine if the mean z-statistic of the proteins that it changes in function is lower than that of other proteins. It is hypothesised that drugs with smaller t-test p-values are more likely to be effective. In this analysis, we included only drugs that change the function of at least two proteins, as at least two samples per group are required for performing a t-test in R (version 3.6.1).

### Adoption of the ‘disease-association of all proteins affected and the magnitude of effect’ method

As the best results were achieved with our ‘Disease-association of all proteins affected and the magnitude of effect’ method, it was adopted for generating the FM score.

The ‘disease-association of all proteins affected and the magnitude of effect’ analysis was repeated with a more comprehensive dataset (extracted from additional databases) of drugs and the proteins that they change in function. In order to reduce the computational burden, the dataset was limited to prescribable drugs (Brown and Patel, 2017b; Santos *et al.*, 2017), including all drugs used effectively (now or in the past) to treat epilepsy (see above). We used the following additional databases of drugs and the proteins that they change in function. We wished to limit drug-protein pairs to those in which drugs affect the *function* of their target proteins. For this, each database download was formatted as described below.

1. The Drug-Gene Interaction Database 3.0 (<http://www.dgidb.org/downloads>; accessed 01/09/2020). The dataset was limited to drug-protein pairs with the following types of interactions: agonist, allosteric modulator, antagonist, antibody, binder, blocker, channel blocker, cofactor, gating inhibitor, inhibitor, inhibitory allosteric modulator, inverse agonist, modulator, negative modulator, partial agonist, positive allosteric modulator.
2. STITCH 5.0 (<http://stitch.embl.de/>; accessed 01/09/2020). The dataset was limited to interactions with the following features: ‘mode’ is binding, ‘action’ is inhibition or activation, and ‘a_is_acting’ indicates that the directionality of effect is drug upon protein.
3. DrugBank (<https://www.drugbank.ca/unearth/advanced/drugs>; accessed 01/09/2020). We included all drugs that alter the function of one or more proteins.
4. The ChEMBL database (<https://www.ebi.ac.uk/chembl/g/#browse/mechanisms_of_action>; accessed 12/08/2019), as mentioned above.

The expanded dataset (of drugs and the proteins that they change in function) was not used in the initial method development and benchmarking steps, as the process is computational intensive, and use of the larger dataset exacerbates computational cost.

## Utilising the Abundance Correction (AC) score

*Transcriptomes for subtypes of epilepsy*

Transcriptomes were imputed from GWAS summary statistics with FUSION (Gusev *et al.*, 2016), as described previously (International League Against Epilepsy Consortium on Complex Epilepsies, 2018). Briefly, differentially expressed genes were imputed from GWAS summary statistics with FUSION (Gusev *et al.*, 2016) using dorsolateral prefrontal cortex tissue RNA-sequencing data (n=452), obtained from the CommonMind consortium (Fromer *et al.*, 2016). We calculated z-scores for the association between phenotype and changes in expression of all significantly heritable genes.

The dorsolateral prefrontal cortex was chosen as the expression reference tissue as it showed the highest enrichment in the tissue-specific heritability enrichment analysis performed in our previous study (International League Against Epilepsy Consortium on Complex Epilepsies, 2018). In that study, dorsolateral prefrontal cortex was used for imputing expression for all the epilepsy types/syndromes, and revealed significant findings in multiple epilepsy types/syndromes. Hippocampus expression reference data was sought for imputing differentially expressed genes from the HS GWAS. However, the largest available hippocampus expression reference dataset (n=111) was too small for effective imputation. It should be noted that local gene expression regulation is shared across tissues (Consortium, 2015; Consortium *et al*., 2017; Liu *et al*., 2017), and expression reference data from organs/tissues entirely unrelated to a disease can be used to impute the genes differentially expressed in the disease, for example, adipose tissue for schizophrenia (Gusev *et al*., 2018), and dorsolateral prefrontal cortex for prostate cancer (Mancuso *et al*., 2018).

*Using transcriptomic data to prioritise drugs with antiepileptic efficacy*

Empirically determining the optimal list-length of imputed differentially-expressed genes for drug prioritisation: Currently, there is no consensus on the optimal list-length of imputed differentially-expressed genes to use for generating drug prioritisation—whole lists or subsets of various sizes have been arbitrarily employed in previous studies (So *et al.*, 2017; International League Against Epilepsy Consortium on Complex , 2018). Excessively trimming the list to only the most significantly differentially expressed genes risks omitting many functionally-relevant gene expression changes that could inform the drug prediction analysis, whereas expanding the list excessively risks including spurious findings that will compromise the drug prediction analysis. Where the balance lies, for any individual experiment, cannot be determined *a priori*. The power of the study to detect differential expression and the heritability of gene expression changes underlying the disease will influence the optimal list-length size. Hence, for the current study, we empirically determined the optimal list-length.

Our methodology is based on the premise that the optimal list-length is the one that provides the highest rankings for validated drugs. Our hypothesis was as follows: for any disease transcriptome, the highest ranking of validated drugs is obtained using optimal list-length *l*, and using progressively longer or shorter lists leads to a progressive decay in the ranking of validated drugs. To test this hypothesis, we imputed the differentially-expressed genes for AE from its GWAS. Then, the imputed genes were ranked by absolute z-score for the association between phenotype and change in gene expression, and progressively longer lists of differentially-expressed genes were created, increasing in increments from the top 5% to 100% genes. For the drug prioritisation obtained using each list-length, drugs were ranked by cosine distance, from the most negative (most therapeutic) to the most positive (least therapeutic). For each list, the median rank (see below for details) of the validated drug-set (ASMs) was determined. For consistency with our previously published AE results (International League Against Epilepsy Consortium on Complex Epilepsies, 2018) we used the same set of ASMs for this analysis; using the new set of ASMs produces a similar pattern (data not shown). The results are shown in Supplementary Figure 1 and Supplementary Table 7. The results are consistent with our hypothesis that the highest ranking of validated drugs is obtained using optimal list-length *l*, and using progressively longer or shorter lists leads to a progressive decay in the ranking of validated drugs.

Based upon the above findings, for other epilepsy types, we employed a similar approach in order to identify the optimal list-length. Our strategy was to use progressively longer gene-list lengths, stopping when the median ranking for the validated drug-set peaked and started to decline. The gene-list length providing the peak ranking was selected. A list-length of 1% genes was insufficient to provide drug prediction results for all epilepsy types. Hence, we started with the top 5% genes, and increased list-length in increments of 5%. Results are shown in Supplementary Table 8. For FE, the validated drug-set comprised all ASMs, whereas for the other epilepsy subtypes, the validated drug-set comprised the specific subset of ASMs that it is more effective for the subtype.

To identify drugs able to rectify disease-associated gene-expression changes, we used the Combination Connectivity Mapping (version 1.4.0) bioconductor package (<https://bioconductor.org/packages/release/bioc/html/ccmap.html>) and the Library of Integrated Network-Based Cellular Signatures (LINCS) data. This package utilises cosine distance as the (dis)similarity metric. A higher (more negative) cosine distance value indicates that the drug induces gene-expression changes more strongly opposed to those associated with the disease. A lower (more positive) cosine distance value indicates that the drug induces gene-expression changes more similar to those associated with the disease. In the LINCS dataset, some drugs have been profiled in more than one cell line, concentration and time-point. For such drugs, the highest absolute cosine distance, whether positive or negative, is selected, as this value is least likely to occur by chance. This is a validated strategy for effectively identifying drugs with antiepileptic efficacy (International League Against Epilepsy Consortium on Complex Epilepsies, 2018).

*Can Mendelian randomisation be used to predict drugs for common epilepsies?*

Mendelian randomisation has been used to predict drugs for common diseases from their GWAS results. Mendelian randomisation for drug repurposing, as originally designed, has the prerequisites listed below (and illustrated using an example of ‘lipid-lowering drugs for Alzheimer’s disease’) (Williams *et al.*, 2020):

- A measurable and modifiable biological trait that is postulated to affect the disease (in our example, lipid levels).
- Candidate drugs that affect the biological trait (in our example, drugs that lower serum lipids).
- Knowledge of the protein target(s) through which the candidate drugs affect the biological trait (for example, HMG-CoA for statins).
- A genome-wide association study of the biological trait (in our example, a genome-wide association study of lipid levels).
- A genome-wide association study of the disease (in our example, a genome-wide association study of Alzheimer’s disease).

Using the above data, it is possible to predict the proteins which when manipulated have a therapeutic effect on the disease through the biological trait studied. Then, potentially repurposable drugs can be predicted based on existing knowledge of the drugs that target the identified proteins to produce the desired effect on the biological trait (Interleukin-6 Receptor Mendelian Randomisation Analysis *et al.*, 2012). Because such a biological trait has not been identified yet for epilepsy, this Mendelian randomisation method cannot be applied to epilepsy at present. In a new implementation of the Mendelian randomisation approach, gene transcript or protein abundance (expression quantitative trait loci or protein quantitative trait loci) are used as the biological trait (Schmidt *et al.*, 2020; Storm *et al.*, 2020). This identifies the protein abundance changes that underlie the disease of interest. This method is closely related to the tissue-wide association study method (Richardson *et al.*, 2020; Zhu and Zhou, 2020) that we have employed in the current study to calculate AC sores. When the tissue-wide association study method and the Mendelian randomisation method were both applied in the same study, most protein abundance changes were identified by both methods (Richardson *et al.*, 2020), but some protein abundance changes were identified by one method but not the either. Neither method was deemed superior. In future studies, both methods could be compared and/or combined for calculating AC scores in order to determine if this improves the drug predictions.

## Developing the Function and Abundance Modulation (FAM) score

The FM and AC scores were converted into their respective z-scores. The FAM score for each drug is the weighted mean of its FM and AC z-scores. The optimal relative weight is determined empirically, based upon the following criterion: The optimal relative weight provides the highest sum of (1) the (mean) AUROC for differentiating ASMs from all other drugs, and (2) the AUROC for differentiating more from less effective ASMs, if applicable. Practically, the optimal relative weight is determined as follows: Whilst holding the relative weight for the FM score constant at 1, the relative weight for the AC score is incrementally increased from 0 to 100, in steps of 0.01. AUROC values are calculated at each step.

## Comparison with existing alternative enhanced methods

*Identifying drugs that affect the function of proteins in the disease-proteins’ interaction network*

In order to expand the number of potentially-effective candidate compounds, some studies identify the drugs that target genome-wide significant disease-proteins and, in addition, the drugs that target the proteins interacting with genome-wide significant disease-proteins (Okada et al., 2014; Wain et al., 2017; Wang et al., 2018). An efficient and validated tool for executing this analysis is the GUILDify v2.0 Web Server (<http://aleph.upf.edu/guildify2/>; accessed 16/08/2019). Using a set of ‘seed’ proteins provided by the user, GUILDify v2.0 constructs a network of proteins that interact with the seeds, and lists drugs that target the seed and the interacting proteins. Guildify was run using default settings (Species: Homo sapiens; Tissue: all; Network: BIANCA; NetScore repetitions 3 and iterations 2). The manuscript of the epilepsy GWAS includes a set of ‘prioritized biological epilepsy genes’ for the AE phenotype (International League Against Epilepsy Consortium on Complex, 2018); these genes were chosen as seeds for generating the protein-protein interaction network in Guildify v2.0, as they are more numerous than the genes that pass the GWAS gene-based Benjamini–Hochberg-corrected p-value <0.05 threshold. The list of drugs that target the seed and the interacting proteins was downloaded from the Guildify v2.0 website. This list of drugs was used to calculate the precision, recall and F-score for predicting ASMs.

*Gene-set t-test method of So, et al.*

We compared our results with those from a recently-published method (So et al., 2018) of using GWAS data to identify effective drugs. In this method, GWAS gene-based p-values are converted to z-statistics. Then, for each drug, a single-sided two-sample t-test is used to determine if the mean z-statistic of the proteins that it changes in function is lower than that of other proteins. It is hypothesised that drugs with smaller t-test p-values are more likely to be effective. In this analysis, we included only drugs that change the function of at least two proteins, as at least two samples per group are required for performing a t-test in R (version 3.6.1).

## Validation of the FAM score.

The FAM score for AE was validated using all ASMs drug-set, and the prioritisation and classification metrics described above. In addition, we tested the following hypothesis: When drugs are ranked by their FAM score for AE, drugs used to treat epilepsy are ranked higher then drugs used to treat any other human disease. To test this hypothesis, the RepoDB database (<http://apps.chiragjpgroup.org/repoDB/>; accessed 01/09/2019) was downloaded. Drugs for the following epilepsies were merged into the epilepsy drug-set: absence epilepsy, epilepsies, epilepsy, epilepsy characterized by intractable complex partial seizures, epileptic drop attack, infantile severe myoclonic epilepsy, mixed epileptic, motor cortex epilepsy, simple partial seizures, status epilepticus, tonic-clonic epilepsy, tonic-clonic seizures. In the database, sertraline is listed as a drug for epilepsy, which is erroneous and, hence, sertraline was deleted from the epilepsy drug-set. Diseases with less than 20 drugs were excluded, leaving 107 diseases in total. We calculated the median rank of the drug-set for each disease, within our ranked drug prioritisations for AE. By randomly permuting our predicted drug rankings (10,000 times), we calculated the chance probability that the median rank of ASMs is (1) highest, and (2) at least as many ranks higher than all other drug-sets used to treat all other human diseases as found in our predictions.

Next, we tested the following postulate: the most accurate drug predictions for a syndrome are obtained by using the FAM score for that syndrome, i.e., in order to predict which ASMs are more clinically-effective and which ASMs are less clinically-effective for a syndrome, the best results are obtained by using the FAM score for that syndrome. For this, we determined if the subset of ASMs that is more effective for CAE is favoured over the subset of ASMs that is less effective for CAE, when drugs are predicted using the FAM scores for CAE, AE, GE, JME, FE or HS. For this analysis, we opted not to use broad- and narrow-spectrum ASMs as they are more and less effective, respectively, for a number of epilepsy subtypes, and we did not use the subset of ASMs more effective and less effective for HS, as these are small drug-sets.

**The prioritised order of efficacy of ASMs for FE matches that seen in the SANAD trial**

The SANAD study is the largest published head-to-head comparison of multiple ASMs for FE, and the largest published randomised controlled trial of ASMs for FE (Marson *et al.*, 2007). ASMs studied in the FE arm of SANAD were carbamazepine, gabapentin, lamotrigine, oxcarbazepine and topiramate. Time to 12-month remission, in the intention-to-treat analysis, was the primary efficacy-based outcome measure of the SANAD trial. Using this outcome measure, we ranked the studied ASMs according to order of efficacy. According to the published results, carbamazepine was the ‘preferred treatment for this outcome in all pair-wise comparisons’. Hence, carbamazepine was ranked highest. In order to assign positions to the other ASMs, we examined their hazard ratios (point estimates and 95% confidence intervals) compared to carbamazepine, as provided in the results of the SANAD trial. These results are plotted in Supplementary Figure 2.

As shown in Supplementary Figure 2, gabapentin is least effective based upon the point estimates and limits of the confidence intervals and, hence, gabapentin was ranked lowest. Topiramate is more effective than gabapentin but less effective than oxcarbazepine and lamotrigine based upon the point estimates and limits of the confidence intervals and, hence, topiramate was assigned second to last rank. However, oxcarbazepine and lamotrigine cannot be differentiated as the point estimates are alike (0.92 and 0.91, respectively), and the upper limit of the confidence intervals favours the latter, whilst the lower limit of the confidence intervals favours the former. Hence, oxcarbazepine and lamotrigine are ranked below carbamazepine, but the relative ranks of oxcarbazepine and lamotrigine are undetermined. For the five ASMs, we determined if our prioritised order of efficacy matches the observed order. As the relative observed ranks of oxcarbazepine and lamotrigine are undetermined, prediction is considered consistent with observation if both oxcarbazepine and lamotrigine are prioritised less effective than carbamazepine but more effective than the other ASMs, regardless of the relative efficacies of oxcarbazepine and lamotrigine. By permutation, we determined the probability that these drugs are ranked as highly and in the correct order purely by chance.

**Do our drug predictions identify and prioritise the compounds being tested as ASMs in clinical trials?**

We determined whether the compounds that are being tested as ASMs in clinical trials, even though they are licensed for other conditions, are identified and prioritised by our drug predictions. ClinicalTrials.gov (accessed on the 27^th^ of January, 2021) was searched for epilepsy drug trials. We excluded drugs being trialled for single monogenic epilepsy syndromes (for example, ganaxolone for female children with PCDH19-related epilepsy), and drugs that suppress seizures through pharmacokinetic potentiation of concomitantly-given conventional antiseizure drugs (for example, verapamil through inhibition of drug-efflux transporters at the blood-brain barrier).

## Are the drug predictions driven by individual highly disease-associated proteins?

For each epilepsy, FAM scores were re-calculated after excluding, one at a time, the top 5 most strongly disease-associated proteins from the TWAS, and the top 5 most strongly disease-associated proteins from the GWAS (Supplementary Table 3). Only proteins that are affected by a drug were considered for exclusion. Proteins that are not affected by a drug do not contribute to the FAM score and, hence, they were not considered for exclusion. Drug ranks obtained after excluding a protein were compared with the original drug ranks, using Kendall’s τ.

## Filtered lists of promising candidate drugs

The optimal FAM score cut-point was determined empirically, and separately, for each phenotype. To determine the optimal FAM score cut-point, the “minimum p-value” approach was adopted. In our implementation of this approach, we sequentially took every observed FAM score as a candidate cut-point, and ran a hypergeometric test for the enrichment of (more effective) ASMs at each of the candidate cut-points. The optimal cut-point is the FAM score that maximises the enrichment of (more effective) ASMs and, thereby, minimises the hypergeometric test p-value. For phenotypes with a more and a less effective subset of ASMs, we also computed the relative enrichment of more effective compounds compared to less effective compounds (by calculating the ratio of the respective hypergeometric test p-values). It is common practice to use the “minimum p-value” approach for optimisation (Colomer *et al.*, 1997; Bouchet *et al.*, 1998; Pappinen *et al.*, 2005; Cella *et al.*, 2009; Mizuno *et al.*, 2009; Park *et al.*, 2009; Teramukai *et al.*, 2009; Mlecnik *et al.*, 2010; Fasching *et al.*, 2011; Hansen *et al.*, 2013; Dunkel *et al.*, 2014; Chalkidou *et al.*, 2015; Diouf *et al.*, 2015; Yamamoto *et al.*, 2015; Vanniyasingam *et al.*, 2016; Kantarelis and Kantarelis, 2017; Groot *et al.*, 2019; Wong *et al.*, 2019), and hypergeometric enrichment for validation (Chiang and Butte, 2009; Jin *et al.*, 2012; Gottlieb and Altman, 2014; Brown *et al.*, 2016a; Issa *et al.*, 2016; Shaked *et al.*, 2016; Brown and Patel, 2017a; Lee and Yoon, 2018), and to combine the two (Gottlieb *et al.*, 2011; Amand *et al.*, 2019)

## Animal model testing

*Choice of model*: As our drug predictions are based upon genetic variants, we tested our predictions using a complex genetic model of epilepsy. Relevant models include genetic models of generalised seizures, of which the DBA/2 mouse model is most well-recognised and widely used, and genetic models of absence seizures, such as GAERS. We considered absence seizures to be a lower priority, as currently available ASMs are well-suited to meeting the needs of patients with absence seizures. Hence, the DBA/2 mouse model was selected for our study.

*Choice of drug candidates*: We tested the top five drug repurposing candidates for GE; this number was based upon resource limitations. We tested the five most highly ranked predictions for GE, after filtering out known ASMs, compounds with existing published evidence in the DBA/2 mouse model, drugs lacking evidence of blood-brain barrier permeability, drugs lacking evidence of safe long-term oral use in humans, compounds insoluble in water or saline, and ‘controlled substances’ that require exceptional legal authorisation for procurement under the laws of France (where the animal experiments were performed). Supplementary Table 9 lists the compounds that were filtered out and the respective reasons. The compounds and doses tested were: acamprosate (125, 250 and 500 mg/kg), betahistine (75, 150 and 300 mg/kg), dyclonine (5, 10 and 20 mg/kg), orphenadrine (12.5, 25 and 50 mg/kg) and trimeprazine (2.5, 5 and 10 mg/kg). Valproate (180 mg/kg i.p.) was used as the reference compound.

*Animal experiment protocol*. The method followed that described by Dürmüller *et al* (Durmuller *et al.*, 1993). Mice (DBA/2, 3-4 weeks old) were placed in a Plexiglas jar (Diameter = 40 cm; Height = 35 cm) mounted with an electric bell (110-120 dB). The bell was activated until a seizure occurred or for a maximum of 60 seconds. The test was performed blind. Latency to tonic and clonic seizures was the outcome for analysis. Each test substance was evaluated at three doses, administered twice, 8 hours and 30 minutes before the test, and compared with a vehicle control (saline) group. All compounds were dissolved in normal saline. All compounds were administered intraperitoneally. Ten mice were tested with each dose; the number of mice was based upon available resources. For each compound, one set of ten mice was administered the highest non-toxic dose, based on literature review, and the next set of ten mice was administered half the highest non-toxic dose, and the final set of ten mice was administered quarter the highest non-toxic dose. Candidate compound treated groups were compared with the vehicle control group using the Kruskal-Wallis test followed by the Mann-Whitney U test. Statistical analysis was performed in R (version 3.4.3). The animal experiments were performed by the Porsolt Research Laboratory (France; <https://www.porsolt.com/>).

*Additional experimental details.*

Species used: Male DBA/2 mice, 3 - 4 weeks old, weighing 8 - 16 g at the beginning of the experiments.

Number: 175 males (including 5 spare).

Reason for selection of species: The characteristics of the animals used (age, strain, and species) are comparable with those described in the scientific literature. In addition, Porsolt maintains historical data for tracking biological responses in positive and negative control groups over time for these standard tests and animals.

Breeder: Janvier Labs, 53940 Le Genest-Saint-Isle, France.

Receipt and acclimation period: Animals were delivered to the laboratory at least 3 days before the experiments during which time they were acclimatized to laboratory conditions.

Identification: Indelible marker on the tail.

Housing and Environmental conditions: Mice were housed grouped in macrolon cages (no more than 5 animals per cage) on wood litter (SAFE, 89290 Augy, France). Environmental enrichment (such as tunnel, gnawing material, nesting material) were provided. The animal house was maintained under artificial lighting (12 hours) between 7:00 and 19:00 in a controlled ambient temperature of 22 ± 2°C, and relative humidity between 30-70%.

Food and water: All animals had free access to food (Code A04 - SAFE, 89290 Augy, France) and water.

*Regulatory compliance*

The study was conducted in compliance with Animal Health regulations, in particular:

- Council Directive No. 2010/63/UE of September 22^nd^ 2010 on the protection of animals used for scientific purposes and French decree No. 2013-118 of February 1^st^ 2013 on the protection of animals;
- Porsolt facility accreditation for experimentation (E 53 1031, renewed on April 19^th^, 2016);
- The recommendations of the Association for Assessment and Accreditation of Laboratory Animal Care (AAALAC) of which the accreditation was granted in June 2012 and renewed in 2018.

In-house ethics review procedure:

Porsolt has an in-house ethics program, which covers animal care and use within the facility. Porsolt’s Ethical Committee evaluates this program to ensure that:

- animal use is carefully considered and fully justified;
- all possibilities for reduction, refinement and replacement have been evaluated;
- every effort is made to achieve a high standard of animal welfare.

The present study plan was declared to Porsolt’s Ethical Committee before the initiation of the study.

### Collating the top proteins that contribute most to the FAM scores

The top proteins that contribute to the FM score: proteins with the highest value for *boosted* inverse GWAS gene-based p-value, as defined above.

The top proteins that contribute to the AC score: proteins with the largest negative value for: {abundance change in transcriptome of disease} × {abundance change in transcriptome of drug}

# Supplementary Results

## Our sequential methodological enhancements, sequentially improve performance

Starting from the standard method, we created three consecutive methodological enhancements, each of which builds progressively upon the last, by incorporating additional features from the data that describes the relationship between drugs-proteins-disease (Supplementary Table 6). Each methodological enhancement outperforms the preceding method for the identification and prioritisation of ASMs (Supplementary Table 10).

## Our methods outperform existing alternative enhanced methods

For benchmarking, we used two existing and contrasting enhanced methods for GWAS-based drug predictions to predict drugs for AE.

*Network-based method*

Like the standard approach, this network-based method (Aguirre-Plans *et al.*, 2019) produces dichotomous categorisation of compounds into drugs that are predicted to be effective or ineffective, and does not score or rank individual drugs according to their relative predicted efficacy. Hence, analysis of predicted drug rankings and AUROC for method evaluation cannot been performed. Overall, this method produces results (recall of 49%, precision of 18%, F-score of 27%) that are less favourable than the standard approach for the detection of ASMs.

*Gene-set analysis method*

For identifying and prioritising ASMs, this method (So *et al.*, 2017) achieved AUROC (mean ± standard deviation) of 0.61±0.04 and average percentile of 64, respectively, which is not as good as our results (Supplementary Table 1).

## Utilising more drug activity data further improves the performance of our best method

As the best results were achieved with our ‘Disease-association of all proteins affected and the magnitude of effect’ method, it was adopted for generating the FM score.

The ‘Disease-association of all proteins affected and the magnitude of effect’ analysis was repeated with a more comprehensive dataset of drugs and the proteins that they change in function. This led to a further improvement in the identification and prioritisation of ASMs (Supplementary Table 1). The more comprehensive dataset of drugs and the proteins that they change in function was extracted from additional databases listed in the Supplementary Methods. This method and these datasets were used for generating the FM score in all subsequent analyses. This expanded dataset (of drugs and the proteins that they change in function) was not used in the initial method development and benchmarking steps, as the process is computational intensive, and use of the larger dataset exacerbates computational cost.

## Integrating the FM and AC scores leads to improved performance

Next, FM and AC scores were integrated to form an aggregate score: the disease-protein **f**unction and **a**bundance **m**odulation (FAM) score. The AE FAM score is superior to the AE FM and AC scores for identifying and prioritising ASMs (Supplementary Table 1); the AE FAM score’s performance is statistically significant (permutation-based p-value < 1 x 10^–6^).

## Determining whether the drug predictions are dominated by the FM or AC sore

We determined if the drug rankings predicted by the FAM score are correlated more strongly with the drug rankings predicted by the FM score or the drug rankings predicted by the AC core. For HS, the correlation between the drug rankings predicted by the FAM and FM scores is 8x stronger than the correlation between the drug rankings predicted by the FAM and AC scores. For CAE, the correlation between the drug rankings predicted by the FAM and AC scores is 4x stronger than the correlation between the drug rankings predicted by the FAM and FM scores. For the other epilepsies, relative correlations lie between this range (see below).

The correlation between drug ranks based on FAM and FM scores, relative to the correlation between drug ranks based on FAM and AC scores for:

- AE = 2.2
- GE = 1.3
- JME = 4.0
- CAE = 0.2
- FE = 2.1
- HS = 8.0

Correlation was measured using Kendall’s tau.

## The FAM score predicts compounds being tested as ASMs in clinical trials

We determined whether the compounds that are being tested in clinical trials as ASMs, even though they are licensed for other conditions, are identified and prioritised by our drug predictions. We identified two drugs that are licensed for conditions other than epilepsy but are being tested as ASMs in clinical trials: memantine (<https://clinicaltrials.gov/ct2/show/NCT03779672>) and voronistat (<https://clinicaltrials.gov/ct2/show/NCT03894826>). Both drugs are being trialled in children with multiple different types of epilepsies. Broad-spectrum ASMs are most suitable for this cohort. Our GE predictions identify and prioritise broad-spectrum antiseizure drugs. Interestingly, both memantine and voronistat are identified within our top predictions for GE (memantine is ranked 28^th^ and voronistat is ranked 45^th^). The antiseizure efficacy of memantine in rodent models has long been recognised (Apland and Cann, 1995; Cakil *et al.*, 2011; Zaitsev *et al.*, 2015; Amador *et al.*, 2020; Dogan *et al.*, 2020). Voronistat was recently identified as a potential ASM through screening of 870 compounds in multiple zebrafish models of seizures, followed by validation in multiple rodent models of seizures (Ibhazehiebo *et al.*, 2018).

## Additional analyses on the top five predicted drugs for GE that we tested in a mouse model

We performed the following additional analyses on the top five predicted drugs for GE that we tested in a mouse model:

The five predicted drugs, which that we tested in an animal model, are not heavily dominated by the same set of proteins. We collated the top proteins that contribute most to the FAM scores of the five drugs: for each drug, the top three proteins that contribute to its FM score, and the top three proteins that contribute to its AC score. None of these proteins contributes to the FAM score of more than two drugs; most of these proteins (65%) contribute to the FAM score of one drug only. (See Supplementary Methods for how the top proteins were identified.)

Dyclonine and acamprosate are two of the drugs we tested in an animal model. It was noted that one of the proteins affected by dyclonine is SCN1A, and one of the proteins affected by acamprosate is GABRA2. These two proteins are strongly associated with epilepsy. For dyclonine and acamprosate, we excluded from our analysis the other proteins they affect, retaining only SCN1A for dyclonine and GABRA2 for acamprosate. With this, the predicted rank for dyclonine fell from 1 to 80, and for acamprosate from 3 to 82. This suggests that the high predicted ranks of these drugs cannot be explained by individual highly disease-associated proteins, but rather rely on multiple diverse proteins affected in function and abundance by the drugs.

## References for Table 2

1. Ohno Y, Sofue N, Imaoku T, et al. Serotonergic modulation of absence-like seizures in groggy rats: a novel rat model of absence epilepsy. *J Pharmacol Sci*. 2010;114(1):99-105. doi:10.1254/jphs.10156fp

2. Sakakihara Y, Oka A, Kubota M, Ohashi Y. Reduction of seizure frequency with clomipramine in patients with complex partial seizures. *Brain Dev*. Jul-Aug 1995;17(4):291-3. doi:10.1016/0387-7604(95)00040-i

3. Palmer GC, Harris EW, Ray R, Stagnitto ML, Schmiesing RJ. Classification of compounds for prevention of NMDLA-induced seizures/mortality, or maximal electroshock and pentylenetetrazol seizures in mice and antagonism of MK801 binding in vitro. *Arch Int Pharmacodyn Ther*. May-Jun 1992;317:16-34.

4. Sun XY, Zhang L, Wei CX, Piao HR, Quan ZS. Characterization of the anticonvulsant activity of doxepin in various experimental seizure models in mice. *Pharmacol Rep*. Mar-Apr 2009;61(2):245-51. doi:10.1016/s1734-1140(09)70028-3

5. Ahmad M, Abu-Taweel GM, Aboshaiqah AE, Ajarem JS. The effects of quinacrine, proglumide, and pentoxifylline on seizure activity, cognitive deficit, and oxidative stress in rat lithium-pilocarpine model of status epilepticus. *Oxid Med Cell Longev*. 2014;2014:630509. doi:10.1155/2014/630509

6. Plotnikoff N, Huang J, Havens P. Effect of monoamino oxidase inhibitors on audiogenic seizures. *Journal of pharmaceutical sciences*. 1963;52(2):172-173.

7. Wallenstein MC, Mauss EA. Effect of prostaglandin synthetase inhibitors on experimentally induced convulsions in rats. *Pharmacology*. 1984;29(2):85-93. doi:10.1159/000137996

8. Stone B, Evans L, Coleman J, Kuebler D. Genetic and pharmacological manipulations that alter metabolism suppress seizure-like activity in Drosophila. *Brain Res*. Feb 16 2013;1496:94-103. doi:10.1016/j.brainres.2012.12.007

9. Loscher W, Lehmann H, Teschendorf HJ, Traut M, Gross G. Inhibition of monoamine oxidase type A, but not type B, is an effective means of inducing anticonvulsant activity in the kindling model of epilepsy. *J Pharmacol Exp Ther*. Mar 1999;288(3):984-92.

10. Kobayashi K, Nishizawa Y, Sawada K, Ogura H, Miyabe M. K(+)-channel openers suppress epileptiform activities induced by 4-aminopyridine in cultured rat hippocampal neurons. *J Pharmacol Sci*. Dec 2008;108(4):517-28. doi:10.1254/jphs.08214fp

11. Zaluska K, Kondrat-Wrobel MW, Luszczki JJ. Comparison of the anticonvulsant potency of various diuretic drugs in the maximal electroshock-induced seizure threshold test in mice. *Adv Clin Exp Med*. May 2018;27(5):609-613. doi:10.17219/acem/68694

12. Hesdorffer DC, Stables JP, Hauser WA, Annegers JF, Cascino G. Are certain diuretics also anticonvulsants? *Ann Neurol*. Oct 2001;50(4):458-62. doi:10.1002/ana.1136

13. Thompson RG, Aldrete JA. Interaction between local anesthetics and analeptic drugs. *South Med J*. Feb 1975;68(2):189-92. doi:10.1097/00007611-197502000-00017

14. Kodama M, Yamada N, Sato K, Sato T, Morimoto K, Kuroda S. The insular but not the perirhinal cortex is involved in the expression of fully-kindled amygdaloid seizures in rats. *Epilepsy Res*. Aug 2001;46(2):169-78. doi:10.1016/s0920-1211(01)00279-0

15. Banach M, Piskorska B, Borowicz-Reutt KK. Propafenone enhances the anticonvulsant action of classical antiepileptic drugs in the mouse maximal electroshock model. *Pharmacol Rep*. Jun 2016;68(3):555-60. doi:10.1016/j.pharep.2016.01.002

16. Palencia G, Calderon A, Sotelo J. Thalidomide inhibits pentylenetetrazole-induced seizures. *J Neurol Sci*. Jul 15 2007;258(1-2):128-31. doi:10.1016/j.jns.2007.03.010

17. Palencia G, Rubio C, Custodio-Ramirez V, Paz C, Sotelo J. Strong anticonvulsant effect of thalidomide on amygdaloid kindling. *Epilepsy Res*. Aug 2011;95(3):263-9. doi:10.1016/j.eplepsyres.2011.04.008

18. Payandemehr B, Rahimian R, Gooshe M, et al. Nitric oxide mediates the anticonvulsant effects of thalidomide on pentylenetetrazole-induced clonic seizures in mice. *Epilepsy Behav*. May 2014;34:99-104. doi:10.1016/j.yebeh.2014.03.020

19. Gaudreault J, Varin F, Pollack GM. Pharmacokinetics and anticonvulsant effect of a new hypnotic, CL 284,846, in rats. *Pharm Res*. Nov 1995;12(11):1592-7. doi:10.1023/a:1016224629614

20. Vlainic J, Pericic D. Effects of acute and repeated zolpidem treatment on pentylenetetrazole-induced seizure threshold and on locomotor activity: comparison with diazepam. *Neuropharmacology*. Jun 2009;56(8):1124-30. doi:10.1016/j.neuropharm.2009.03.010

21. Vlainic J, Pericic D. Zolpidem is a potent anticonvulsant in adult and aged mice. *Brain Res*. Jan 15 2010;1310:181-8. doi:10.1016/j.brainres.2009.11.018

22. Sheikhi M, Shirzadian A, Dehdashtian A, et al. Involvement of ATP-sensitive potassium channels and the opioid system in the anticonvulsive effect of zolpidem in mice. *Epilepsy Behav*. Sep 2016;62:291-6. doi:10.1016/j.yebeh.2016.07.014

23. Ozbakis-Dengiz G, Bakirci A. Anticonvulsant and hypnotic effects of amiodarone. *J Zhejiang Univ Sci B*. Apr 2009;10(4):317-22. doi:10.1631/jzus.B0820316

24. Pelletier MR, Corcoran ME. Intra-amygdaloid infusions of clonidine retard kindling. *Brain Res*. Dec 11 1992;598(1-2):51-8. doi:10.1016/0006-8993(92)90167-8

25. Ormandy GC, Song L, Jope RS. Analysis of the convulsant-potentiating effects of lithium in rats. *Exp Neurol*. Mar 1991;111(3):356-61. doi:10.1016/0014-4886(91)90103-j

26. Gilbert ME, Mack CM. Enhanced susceptibility to kindling by chlordimeform may be mediated by a local anesthetic action. *Psychopharmacology (Berl)*. 1989;99(2):163-7. doi:10.1007/bf00442802

27. McIntyre DC, Giugno L. Effect of clonidine on amygdala kindling in normal and 6-hydroxydopamine-pretreated rats. *Exp Neurol*. Jan 1988;99(1):96-106. doi:10.1016/0014-4886(88)90130-6

28. Loscher W, Czuczwar SJ. Comparison of drugs with different selectivity for central alpha 1-and alpha 2-adrenoceptors in animal models of epilepsy. *Epilepsy Res*. May 1987;1(3):165-72. doi:10.1016/0920-1211(87)90037-4

29. Scotti de Carolis A, Passarelli F, Pezzola A. Study on the anticonvulsant activity of clonidine against pentylenetetrazol-induced seizures in rats: pharmacological evidence of alpha 2-adrenoceptors mediation. *Arch Int Pharmacodyn Ther*. Aug 1986;282(2):209-18.

30. Gellman RL, Kallianos JA, McNamara JO. Alpha-2 receptors mediate an endogenous noradrenergic suppression of kindling development. *J Pharmacol Exp Ther*. Jun 1987;241(3):891-8.

31. Kotlinska J, Langwinski R. Involvement of opioid and other systems in ethanol abstinence audiogenic seizures in the rat? *Pol J Pharmacol Pharm*. Mar-Apr 1985;37(2):103-11.

32. Horton R, Anlezark G, Meldrum B. Noradrenergic influences on sound-induced seizures. *J Pharmacol Exp Ther*. Aug 1980;214(2):437-42.

33. Lazarova M, Samanin R. Potentiation by yohimbine of pentylenetetrazol-induced seizures in rats: role of alpha 2 adrenergic receptors. *Pharmacol Res Commun*. Apr 1983;15(4):419-25. doi:10.1016/s0031-6989(83)80051-4

34. Tacke U, Kolonen S. The effect of clonidine and yohimbine on audiogenic seizures (AGS) in rats. *Pharmacol Res Commun*. Oct 1984;16(10):1019-30. doi:10.1016/s0031-6989(84)80066-1

35. Lazarova M, Samanin R. Serotonin mediation of the protective effect of clonidine against pentylenetetrazol-induced seizures in rats. *Life Sci*. May 16 1983;32(20):2343-8. doi:10.1016/0024-3205(83)90764-6

36. Ohata K, Murata T, Sakamoto H, et al. [Pharmacological studies of guanabenz: effects of the central nervous system]. *Nihon Yakurigaku Zasshi*. Dec 1982;80(6):471-80.

37. Kulkarni SK. Actions of clonidine on convulsions and behaviour. *Arch Int Pharmacodyn Ther*. Jul 1981;252(1):124-32.

38. Amabeoku G, Chandomba R. Strychnine-induced seizures in mice: the role of noradrenaline. *Prog Neuropsychopharmacol Biol Psychiatry*. Jul 1994;18(4):753-63. doi:10.1016/0278-5846(94)90082-5

39. Amabeoku GJ. Gamma-aminobutyric acid and glutamic acid receptors may mediate theophylline-induced seizures in mice. *Gen Pharmacol*. Mar 1999;32(3):365-72. doi:10.1016/s0306-3623(98)00201-8

40. Enginar N, Yamanturk P, Nurten A, Koyuncuoglu H. Scopolamine-induced convulsions in food given fasted mice: effects of clonidine and tizanidine. *Epilepsy Res*. Jun 1999;35(2):155-60. doi:10.1016/s0920-1211(99)00008-x

41. Shafaroodi H, Moezi L, Bahremand A, Dehpour AR. The role of alpha(2)-adrenoceptors in the anti-convulsant effects of cannabinoids on pentylenetetrazole-induced seizure threshold in mice. *Eur J Pharmacol*. Aug 15 2013;714(1-3):1-6. doi:10.1016/j.ejphar.2013.05.040

42. Read MI, Andreianova AA, Harrison JC, Goulton CS, Sammut IA, Kerr DS. Cardiac electrographic and morphological changes following status epilepticus: effect of clonidine. *Seizure*. Jan 2014;23(1):55-61. doi:10.1016/j.seizure.2013.09.012

43. Read MI, Harrison JC, Kerr DS, Sammut IA. Atenolol offers better protection than clonidine against cardiac injury in kainic acid-induced status epilepticus. *Br J Pharmacol*. Oct 2015;172(19):4626-38. doi:10.1111/bph.13132

44. Shouse MN, Scordato JC, Farber PR, de Lanerolle N. The alpha2 adrenoreceptor agonist clonidine suppresses evoked and spontaneous seizures, whereas the alpha2 adrenoreceptor antagonist idazoxan promotes seizures in amygdala-kindled kittens. *Brain Res*. Mar 16 2007;1137(1):58-68. doi:10.1016/j.brainres.2006.12.033

45. Yan QS, Dailey JW, Steenbergen JL, Jobe PC. Anticonvulsant effect of enhancement of noradrenergic transmission in the superior colliculus in genetically epilepsy-prone rats (GEPRs): a microinjection study. *Brain Res*. Jan 12 1998;780(2):199-209. doi:10.1016/s0006-8993(97)01139-6

46. al-Tajir G, Starr MS. D-2 agonists protect rodents against pilocarpine-induced convulsions by stimulating D-2 receptors in the striatum, but not in the substantia nigra. *Pharmacol Biochem Behav*. May 1991;39(1):109-13. doi:10.1016/0091-3057(91)90405-q

47. Kamal JA, Nadig RS, Joseph T, David J. Effect of calcium channel blockers on experimentally induced seizures in rats. *Indian J Exp Biol*. Jul 1990;28(7):605-8.

48. Lukawski K, Raszewski G, Czuczwar SJ. Interactions of aliskiren, a direct renin inhibitor, with antiepileptic drugs in the test of maximal electroshock in mice. *Eur J Pharmacol*. Jan 15 2018;819:108-113. doi:10.1016/j.ejphar.2017.11.037

49. Lukawski K, Raszewski G, Czuczwar SJ. Effect of aliskiren, a direct renin inhibitor, on the protective action of antiepileptic drugs against pentylenetetrazole-induced clonic seizures in mice. *Fundam Clin Pharmacol*. Apr 2019;33(2):191-198. doi:10.1111/fcp.12421

50. Lembeck F, Beubler E. Convulsions induced by hyperbaric oxygen: inhibition by phenobarbital, diazepam and baclofen. *Naunyn Schmiedebergs Arch Pharmacol*. Mar 1977;297(1):47-51. doi:10.1007/bf00508809

51. Amabeoku GJ, Chikuni O. Effects of some GABAergic agents on quinine-induced seizures in mice. *Experientia*. Jul 15 1992;48(7):659-62. doi:10.1007/bf02118313

52. Amabeoku G. Involvement of GABAergic mechanisms in chloroquine-induced seizures in mice. *Gen Pharmacol*. Mar 1992;23(2):225-9. doi:10.1016/0306-3623(92)90015-c

53. Aley KO, Kulkarni SK. Altered response to GABAergic agents following electro and chemo convulsions in mice. *Indian J Exp Biol*. Mar 1991;29(3):241-3.

54. Gautam SK, Kulkarni SK. GABA/BZ-and NMDA-receptor interaction in digoxin-induced convulsions in rats. *Indian J Exp Biol*. Jul 1991;29(7):636-40.

55. Turski L, Niemann W, Stephens DN. Differential effects of antiepileptic drugs and beta-carbolines on seizures induced by excitatory amino acids. *Neuroscience*. 1990;39(3):799-807. doi:10.1016/0306-4522(90)90262-3

56. Wurpel JN, Sperber EF, Moshe SL. Baclofen inhibits amygdala kindling in immature rats. *Epilepsy Res*. Jan-Feb 1990;5(1):1-7. doi:10.1016/0920-1211(90)90060-9

57. Sperber EF, Wurpel JN, Moshe SL. Evidence for the involvement of nigral GABAB receptors in seizures of rat pups. *Brain Res Dev Brain Res*. May 1 1989;47(1):143-6. doi:10.1016/0165-3806(89)90117-x

58. Frye GD, McCown TJ, Breese GR, Peterson SL. GABAergic modulation of inferior colliculus excitability: role in the ethanol withdrawal audiogenic seizures. *J Pharmacol Exp Ther*. May 1986;237(2):478-85.

59. Uemura S, Kimura H. Amygdaloid kindling with bicuculline methiodide in rats. *Exp Neurol*. Dec 1988;102(3):346-53. doi:10.1016/0014-4886(88)90230-0

60. Dzoljic MR, v d Poel-Heisterkamp AL. The effects of GABA-ergic drugs on enkephalin-induced motor seizure phenomena in the rat. *Clin Exp Pharmacol Physiol*. Mar-Apr 1981;8(2):141-50. doi:10.1111/j.1440-1681.1981.tb00145.x

61. Georgiev VP, Lazarova MB, Kambourova TS. Further evidence for the interactions between angiotensin II and GABAergic transmission in pentylenetetrazol kindling seizures in mice. *Neuropeptides*. Jan 1995;28(1):29-34. doi:10.1016/0143-4179(95)90071-3

62. Faingold CL, Marcinczyk MJ, Casebeer DJ, Randall ME, Arneric SP, Browning RA. GABA in the inferior colliculus plays a critical role in control of audiogenic seizures. *Brain Res*. Mar 21 1994;640(1-2):40-7. doi:10.1016/0006-8993(94)91855-4

63. Young D, Dragunow M. Status epilepticus may be caused by loss of adenosine anticonvulsant mechanisms. *Neuroscience*. Jan 1994;58(2):245-61. doi:10.1016/0306-4522(94)90032-9

64. Wurpel JN. Baclofen prevents rapid amygdala kindling in adult rats. *Experientia*. May 15 1994;50(5):475-8. doi:10.1007/bf01920750

65. Garant DS, Xu SG, Sperber EF, Moshe SL. The influence of thalamic GABA transmission on the susceptibility of adult rats to flurothyl induced seizures. *Epilepsy Res*. Jul 1993;15(3):185-92. doi:10.1016/0920-1211(93)90055-c

66. Veliskova J, Velisek L, Moshe SL. Age-specific effects of baclofen on pentylenetetrazol-induced seizures in developing rats. *Epilepsia*. Aug 1996;37(8):718-22. doi:10.1111/j.1528-1157.1996.tb00641.x

67. Kubova H, Haugvicova R, Mares P. Moderate anticonvulsant action of baclofen does not change during development. *Biol Neonate*. 1996;69(6):405-12. doi:10.1159/000244338

68. Chen L, Chan YS, Yung WH. GABA-B receptor activation in the rat globus pallidus potently suppresses pentylenetetrazol-induced tonic seizures. *J Biomed Sci*. Jul-Aug 2004;11(4):457-64. doi:10.1007/bf02256094

69. Mussi-Ribeiro A, Miranda A, Gobbo-Netto L, Peporine Lopes N, dos Santos WF. A anticonvulsive fraction from Scaptocosa raptoria (Araneae: Lycosidae) spider venom. *Neurosci Lett*. Nov 23 2004;371(2-3):171-5. doi:10.1016/j.neulet.2004.08.064

70. Liberato JL, Cunha AO, Mortari MR, et al. Anticonvulsant and anxiolytic activity of FrPbAII, a novel GABA uptake inhibitor isolated from the venom of the social spider Parawixia bistriata (Araneidae: Araneae). *Brain Res*. Dec 8 2006;1124(1):19-27. doi:10.1016/j.brainres.2006.09.052

71. Mares P, Slamberova R. Opposite effects of a GABA(B) antagonist in two models of epileptic seizures in developing rats. *Brain Res Bull*. Dec 11 2006;71(1-3):160-6. doi:10.1016/j.brainresbull.2006.08.013

72. Malfatti CR, Perry ML, Schweigert ID, et al. Convulsions induced by methylmalonic acid are associated with glutamic acid decarboxylase inhibition in rats: a role for GABA in the seizures presented by methylmalonic acidemic patients? *Neuroscience*. Jun 8 2007;146(4):1879-87. doi:10.1016/j.neuroscience.2007.03.022

73. Mares P, Tabashidze N. Contradictory effects of GABA-B receptor agonists on cortical epileptic afterdischarges in immature rats. *Brain Res Bull*. Jan 31 2008;75(1):173-8. doi:10.1016/j.brainresbull.2007.09.001

74. Pacey LK, Heximer SP, Hampson DR. Increased GABA(B) receptor-mediated signaling reduces the susceptibility of fragile X knockout mice to audiogenic seizures. *Mol Pharmacol*. Jul 2009;76(1):18-24. doi:10.1124/mol.109.056127

75. Brown JW, Moeller A, Schmidt M, et al. Anticonvulsant effects of structurally diverse GABA(B) positive allosteric modulators in the DBA/2J audiogenic seizure test: Comparison to baclofen and utility as a pharmacodynamic screening model. *Neuropharmacology*. Feb 2016;101:358-69. doi:10.1016/j.neuropharm.2015.10.009

76. Tyurenkov IN, Borodkina LE, Bagmetova VV, Berestovitskaya VM, Vasil'eva OS. Comparison of Nootropic and Neuroprotective Features of Aryl-Substituted Analogs of Gamma-Aminobutyric Acid. *Bull Exp Biol Med*. Feb 2016;160(4):465-9. doi:10.1007/s10517-016-3198-4

77. Jiang KW, Gao F, Shui QX, Yu ZS, Xia ZZ. Effect of diazoxide on regulation of vesicular and plasma membrane GABA transporter genes and proteins in hippocampus of rats subjected to picrotoxin-induced kindling. *Neurosci Res*. Nov 2004;50(3):319-29. doi:10.1016/j.neures.2004.08.001

78. Huang CW, Wu SN, Cheng JT, Tsai JJ, Huang CC. Diazoxide reduces status epilepticus neuron damage in diabetes. *Neurotox Res*. May 2010;17(4):305-16. doi:10.1007/s12640-009-9104-3

79. Taha AY, Huot PS, Reza-Lopez S, et al. Seizure resistance in fat-1 transgenic mice endogenously synthesizing high levels of omega-3 polyunsaturated fatty acids. *J Neurochem*. Apr 2008;105(2):380-8. doi:10.1111/j.1471-4159.2007.05144.x

80. Xiao Y, Li X. Polyunsaturated fatty acids modify mouse hippocampal neuronal excitability during excitotoxic or convulsant stimulation. *Brain Res*. Oct 30 1999;846(1):112-21. doi:10.1016/s0006-8993(99)01997-6

81. Omrani S, Taheri M, Omrani MD, Arsang-Jang S, Ghafouri-Fard S. The effect of omega-3 fatty acids on clinical and paraclinical features of intractable epileptic patients: a triple blind randomized clinical trial. *Clin Transl Med*. Jan 16 2019;8(1):3. doi:10.1186/s40169-019-0220-2

82. Ibrahim FAS, Ghebremeskel K, Abdel-Rahman ME, et al. The differential effects of eicosapentaenoic acid (EPA) and docosahexaenoic acid (DHA) on seizure frequency in patients with drug-resistant epilepsy - A randomized, double-blind, placebo-controlled trial. *Epilepsy Behav*. Oct 2018;87:32-38. doi:10.1016/j.yebeh.2018.08.016

83. DeGiorgio CM, Miller PR, Harper R, et al. Fish oil (n-3 fatty acids) in drug resistant epilepsy: a randomised placebo-controlled crossover study. *J Neurol Neurosurg Psychiatry*. Jan 2015;86(1):65-70. doi:10.1136/jnnp-2014-307749

84. Yoon JR, Lee EJ, Kim HD, Lee JH, Kang HC. Polyunsaturated fatty acid-enriched diet therapy for a child with epilepsy. *Brain Dev*. Feb 2014;36(2):163-6. doi:10.1016/j.braindev.2013.01.017

85. Yuen AW, Sander JW, Fluegel D, et al. Omega-3 fatty acid supplementation in patients with chronic epilepsy: a randomized trial. *Epilepsy Behav*. Sep 2005;7(2):253-8. doi:10.1016/j.yebeh.2005.04.014

86. Akarsu ES, Ayhan IH. Effects of intracerebral iloprost injections on motor activity and chemically-induced seizures in rats. *Methods Find Exp Clin Pharmacol*. Sep 1992;14(7):517-22.

87. Gamaniel K, Wuorela H, Vapaatalo H. Effects of iloprost, prostaglandin E1 (PGE1) and prostacyclin (PGI2) on chemically and electrically induced seizures in mice. *Prostaglandins Leukot Essent Fatty Acids*. Feb 1989;35(2):63-8. doi:10.1016/0952-3278(89)90166-x

88. Foster LA, Johnson MR, MacDonald JT, et al. Infantile Epileptic Encephalopathy Associated With SCN2A Mutation Responsive to Oral Mexiletine. *Pediatr Neurol*. Jan 2017;66:108-111. doi:10.1016/j.pediatrneurol.2016.10.008

89. Borowicz KK, Banach M. Antiarrhythmic drugs and epilepsy. *Pharmacol Rep*. Aug 2014;66(4):545-51. doi:10.1016/j.pharep.2014.03.009

90. Nakazawa M, Okumura A, Niijima S, et al. Oral mexiletine for lidocaine-responsive neonatal epilepsy. *Brain Dev*. Aug 2013;35(7):667-9. doi:10.1016/j.braindev.2012.10.011

91. Enoki H, Hata H, Ohmori I, Maniwa S, Ohta H, Kobayashi K. [Clinical applications and the effect of mexiletine on refractory epilepsies]. *No To Hattatsu*. Jan 2000;32(1):29-34.

92. Miyamoto A, Takahashi S, Oki J. [A successful treatment with intravenous lidocaine followed by oral mexiletine in a patient with Lennox-Gastaut syndrome]. *No To Hattatsu*. Sep 1999;31(5):459-64.

93. Kohyama J, Shimohira M, Watanabe S, Fukuda C, Iwakawa Y. Mexiletine hydrochloride in an infant with intractable epilepsy. *Brain Dev*. 1988;10(4):258-60. doi:10.1016/s0387-7604(88)80009-3

94. Kryzhanovskii GN, Shandra AA. [Use of nicotinamide and pyridoxal-5-phosphate to treat experimental epilepsy]. *Zh Nevropatol Psikhiatr Im S S Korsakova*. 1981;81(6):801-9. Primenenie nikotinamida i piridoksal'-5-fosfata dlia kupirovaniia eksperimental'noi epilepsii.

95. Bourgeois BF, Dodson WE, Ferrendelli JA. Potentiation of the antiepileptic activity of phenobarbital by nicotinamide. *Epilepsia*. Apr 1983;24(2):238-44. doi:10.1111/j.1528-1157.1983.tb04885.x

96. Akhundov RA, Dzhafarova SA, Aliev AN. [The search for new anticonvulsant agents based on nicotinamide]. *Eksp Klin Farmakol*. Jan-Feb 1992;55(1):27-9. Izyskanie novykh protivosudorozhnykh sredstv na osnove nikotinamida.

97. Maitre M, Chesielski L, Lehmann A, Kempf E, Mandel P. Protective effect of adenosine and nicotinamide against audiogenic seizure. *Biochem Pharmacol*. Oct 15 1974;23(20):2807-16. doi:10.1016/0006-2952(74)90054-9

98. Kryzhanovskii GN, Shandra AA, Godlevskii LS, Nikushkin EV. [Effect of pantogam, nicotinamide, and phenazepam on seizure activity]. *Biull Eksp Biol Med*. Sep 1982;94(9):61-4. Vliianie pantogama, nikotinamida i fenazepama na sudorozhnuiu aktivnost'.

99. Kryzhanovskii GN, Shandra AA, Godlevskii LS. [Effect of vitamin preparations on epileptic activity]. *Biull Eksp Biol Med*. Aug 1984;98(8):150-3. Vliianie nekotorykh vitaminnykh preparatov na epilepticheskuiu aktivnost'.

100. Kryzhanovskii GN, Shandra AA, Godlevskii LS. [Effect of nicotinamide on generalized convulsions in mice]. *Farmakol Toksikol*. Nov-Dec 1982;45(6):13-7. Vliianie nikotinamida na generalizovannye sudorogi u myshei.

101. Braslavskii VE, Shchavelev VA, Kryzhanovskii GN, Nikushkin EV, Germanov SB. [Effect of nicotinamide on focal and generalized epileptic activity in the cerebral cortex]. *Biull Eksp Biol Med*. Aug 1982;94(8):39-42. Vliianie nikotinamida na ochagovuiu i generalizovannuiu epilepticheskuiu aktivnost' v kore golovnogo mozga.

102. Lapin IP. Nicotinamide, inosine and hypoxanthine, putative endogenous ligands of the benzodiazepine receptor, opposite to diazepam are much more effective against kynurenine-induced seizures than against pentylenetetrazol-induced seizures. *Pharmacol Biochem Behav*. May 1981;14(5):589-93. doi:10.1016/0091-3057(81)90117-9

103. Kryzhanovskii GN, Shandra AA, Godlevskii LS, Beliaeva AI. [Further study of the antiepileptic properties of nicotinamide]. *Biull Eksp Biol Med*. 1981;91(1):42-5. Dal'neishee izuchenie protivoepilepticheskikh svoistv nikotinamida.

104. Lenz QF, Arroyo DS, Temp FR, et al. Cysteinyl leukotriene receptor (CysLT) antagonists decrease pentylenetetrazol-induced seizures and blood-brain barrier dysfunction. *Neuroscience*. Sep 26 2014;277:859-71. doi:10.1016/j.neuroscience.2014.07.058

105. Takahashi Y, Imai K, Ikeda H, Kubota Y, Yamazaki E, Susa F. Open study of pranlukast add-on therapy in intractable partial epilepsy. *Brain Dev*. Mar 2013;35(3):236-44. doi:10.1016/j.braindev.2012.04.001

106. De Sarro G, Siniscalchi A, Ferreri G, Gallelli L, De Sarro A. NMDA and AMPA/kainate receptors are involved in the anticonvulsant activity of riluzole in DBA/2 mice. *Eur J Pharmacol*. Nov 10 2000;408(1):25-34. doi:10.1016/s0014-2999(00)00709-3

107. Yoshida M, Noguchi E, Tsuru N, Ohkoshi N. Effect of riluzole on the acquisition and expression of amygdala kindling. *Epilepsy Res*. Aug 2001;46(2):101-9. doi:10.1016/s0920-1211(01)00251-0

108. Gondran M, Eckeli AL, Migues PV, Gabilan NH, Rodrigues AL. The crude extract from the sea anemone, Bunodosoma caissarum elicits convulsions in mice: possible involvement of the glutamatergic system. *Toxicon*. Dec 2002;40(12):1667-74. doi:10.1016/s0041-0101(02)00181-2

109. Zgrajka W, Nieoczym D, Czuczwar M, et al. Evidences for pharmacokinetic interaction of riluzole and topiramate with pilocarpine in pilocarpine-induced seizures in rats. *Epilepsy Res*. Feb 2010;88(2-3):269-74. doi:10.1016/j.eplepsyres.2009.11.010

# Supplementary Tables and Figures

Supplementary Table 1. Performance of the three scores, measured by the identification and prioritisation of ASMs. AUROC is calculated using drugs’ scores (see text) from the respective methods. AUROC is computed using the technique of random under-sampling, and presented as mean ± standard deviation (see Supplementary Methods). Prioritisation is calculated using drugs’ ranks, when all drugs have been ranked from highest to lowest predicted effect on the phenotype. Prioritisation result shown is the average (median) rank of ASMs, expressed as a percentile; it is equivalent to the percentage of all drugs ranked below the middle-ranked ASM (see Supplementary Methods). The network-based method (Aguirre-Plans *et al.*, 2019) produces dichotomous categorisation of compounds into drugs that are predicted to be effective or ineffective, and does not score or rank individual drugs according to their relative predicted efficacy. Hence, analysis of predicted drug rankings and AUROC for method evaluation cannot been performed. Results for the network-based method are given in the text above. FM: Function Modulation Score; AC: Abundance Correction Score; FAM: Function and Abundance Modulation Score; AUROC: area under receiver operating characteristic curve; SD: standard deviation

| Method | Identification of ASMs (mean ± SD of AUROC) | Prioritisation of ASMs (average percentile) |
| --- | --- | --- |
| Network | - | - |
| So *et al.* | 0.61±0.04 | 64 |
| FM | 0.79±0.03 | 90 |
| AC | 0.63±0.04 | 77 |
| FAM | 0.83±0.03 | 94 |

Supplementary Table 2. When considering the ability to distinguish more effective ASMs from all drugs and from less effective ASMs, the FAM score outperforms its constituent scores. Data is in the following format: {mean AUROC for discriminating more effective ASMs from all drugs} + {AUROC for discriminating more from less effective ASMs} = {sum}. Detailed metrics are shown in Tables S11 and S12. AUROC: area under receiver operating characteristic; Epi: epilepsy type or syndrome; FM: Function Modulation Score; AC: Abundance Correction Score; FAM: Function and Abundance Modulation Score; CAE: childhood absence epilepsy; GE: generalized epilepsy; HS: focal epilepsy with hippocampal sclerosis; JME: juvenile myoclonic epilepsy.

| Epi | FM | AC | FAM |
| --- | --- | --- | --- |
| HS | {0.63}+{0.80}={1.43} | {0.62}+{0.60}={1.22} | {0.65}+{0.87}={1.52} |
| GE | {0.82}+{0.51}={1.33} | {0.69}+{0.72}={1.41} | {0.85}+{0.71}={1.56} |
| JME | {0.83}+{0.67}={1.50} | {0.73}+{0.73}={1.46} | {0.88}+{0.72}={1.60} |
| CAE | {0.64}+{0.65}={1.29} | {0.71}+{0.77}={1.48} | {0.75}+{0.79}={1.54} |

Supplementary Table 3. The top 5 most strongly disease-associated proteins from the TWAS, and the top 5 most strongly disease-associated proteins from the GWAS, which were excluded for the analysis described in the text. Only proteins that are affected by a drug were considered for exclusion. Proteins that are not affected by a drug do not contribute to the FAM score and, hence, they were not considered for exclusion.

| Epi | GWAS | TWAS |
| --- | --- | --- |
| AE | SCN1A, SCN9A, SLCO3A1, ITPKB, PPARA | PTPRK, NFKBIB, FAM69A, ICAM3, SNX6 |
| GE | SCN1A, LGALS1, HDAC5, GABRA2, DHODH | PTPRK, NFKBIB, FAM69A, H2AFV, TSPAN4 |
| JME | SLC34A2, ALDOA, PLA2G6, SLC25A4, VKORC1 | PCCB, NCAPD2, RNF167, RRP1B, DLD |
| CAE | HCAR1, PSMD13, NNMT, KCNN2, GSTO1 | PSMG1, WRB, RPA1, FAT1, CCDC92 |
| FE | SCN1A, SCN9A, SLCO3A1, CUL4A, TFPI | PTPRK, ICAM3, VPS28, TEX10, MAP2K5 |
| HS | SLC14A2, IMPA2, PKD2L1, PDE7A, RBP1 | TEX10, IGHMBP2, PTPRK, CDC25B, LPGAT1 |

Supplementary Table 4. Broad- and narrow-spectrum ASMs. Because of the time-intensive nature of literature review required for classifying ASMs into these categories, classification was performed only for ASMs that are found in our datasets of drugs’ effects upon protein function and abundance.

| ASM | Subset | Notes |
| --- | --- | --- |
| Acetazolamide | Broad | A broad-spectrum ASM (Neufeld, 2015) |
| Carbamazepine | Narrow | Ineffective against or may exacerbate certain types of generalized onset seizures (Shields and Saslow, 1983; Snead and Hosey, 1985; Horn *et al.*, 1986; Liporace *et al.*, 1994; So *et al.*, 1994; Talwar *et al.*, 1994; Atakli *et al.*, 1998; Parker *et al.*, 1998; Parmeggiani *et al.*, 1998; Prasad *et al.*, 1998; Osorio *et al.*, 2000; Kochen *et al.*, 2002; Marini *et al.*, 2005; Wallengren *et al.*, 2005; Liu *et al.*, 2006) |
| Chlordiazepoxide | Broad | A benzodiazepine with broad-spectrum antiepileptic efficacy (Trinka and Brigo, 2015) |
| Diazepam | Broad | A benzodiazepine with broad-spectrum antiepileptic efficacy (Trinka and Brigo, 2015) |
| Ethotoin | Narrow | A hydantoin derivative, like phenytoin, which is ineffective against or may exacerbate certain types of generalized onset seizures (Dam, 1980; Korberly *et al.*, 1981) |
| Felbamate | Broad | A broad-spectrum ASM (French *et al.*, 1999; Pellock *et al.*, 2006; Leppik and White, 2015) |
| Gabapentin | Narrow | Ineffective against or may exacerbate certain types of generalized onset seizures (Trudeau *et al.*, 1996; Vossler, 1996; Asconape *et al.*, 2000; Genton, 2000; Cho and Hong, 2008; Mantoan and Walker, 2011; Desai *et al.*, 2019) |
| Lamotrigine | Broad | Although lamotrigine-induced exacerbation of myoclonus has been reported (Guerrini *et al.*, 1998; Guerrini *et al.*, 1999; Biraben *et al.*, 2000; Janszky *et al.*, 2000; Carrazana and Wheeler, 2001; Crespel *et al.*, 2005; Genton *et al.*, 2006; Fernandez Corcuera *et al.*, 2008; Tombini *et al.*, 2017) it is amongst the first-line treatments for JME in women of child-bearing age (Mantoan and Walker, 2011; Serafini *et al.*, 2019) and is considered a broad-spectrum ASM (Riaz and Matsuo, 2015) |
| Levetiracetam | Broad | A broad-spectrum ASM (Chong and French, 2015) |
| Lidocaine | Narrow | An intravenous ASM for prolonged seizures or frequent recurrent seizures in neonates and children (Yanagihara *et al.*, 1996; Kobayashi *et al.*, 1999; Mori *et al.*, 2004; Shany *et al.*, 2007; Lundqvist *et al.*, 2013; Holtkamp, 2015; Yozawitz and Moshé, 2015; Weeke *et al.*, 2016) but may provoke generalized onset seizures (Usubiaga *et al.*, 1966; Sundaram, 1987; Hess and Walson, 1988; Smith *et al.*, 1992; Pantuck *et al.*, 1997; Resar and Helfaer, 1998; Lee *et al.*, 1999; DeToledo, 2000; DeToledo *et al.*, 2002; Moran *et al.*, 2004; Dorf *et al.*, 2006; Rezvani *et al.*, 2007; Bouwman and Morre, 2013; Kumar and Manjunath, 2013; Aminiahidashti *et al.*, 2015; Hsieh *et al.*, 2015) |
| Lorazepam | Broad | A benzodiazepine with broad-spectrum antiepileptic efficacy (Trinka and Brigo, 2015) |
| Mephenytoin | Narrow | A hydantoin derivative, like phenytoin, which is ineffective against or may exacerbate certain types of generalized onset seizures (Troupin *et al.*, 1976) |
| Midazolam | Broad | A benzodiazepine with broad-spectrum antiepileptic efficacy (Trinka and Brigo, 2015) |
| Nitrazepam | Broad | A benzodiazepine with broad-spectrum antiepileptic efficacy (Trinka and Brigo, 2015) |
| Oxcarbazepine | Narrow | Ineffective against or may exacerbate certain types of generalized onset seizures (Gelisse *et al.*, 2004; Hahn *et al.*, 2004; Kaddurah and Holmes, 2006; Vendrame *et al.*, 2007; Mantoan and Walker, 2011; Deng *et al.*, 2012; Fanella *et al.*, 2013) |
| Pentobarbital | Broad | A broad-spectrum ASM (Michelucci *et al.*, 2015) |
| Phenytoin | Narrow | Ineffective against or may exacerbate certain types of generalized onset seizures (Eldridge *et al.*, 1983; Atakli *et al.*, 1998; Osorio *et al.*, 2000; Mantoan and Walker, 2011) |
| Primidone | Broad | A broad-spectrum ASM (Michelucci *et al.*, 2015) |
| Propofol | Broad | Broad-spectrum efficacy against all types of seizures in status epilepticus (Walker and Shorvon, 2015) |
| Tiagabine | Narrow | May precipitate or exacerbate certain types of generalized onset seizures (Knake *et al.*, 1999; Zhu and Vaughn, 2002; Skardoutsou *et al.*, 2003; Vollmar and Noachtar, 2007; Mantoan and Walker, 2011) |
| Topiramate | Broad | A broad-spectrum ASM (Cross and Riney, 2015) |
| Trimethadione | Narrow | Effective for absences (Lennox, 1945; Butter, 1948, 1952) and for mesial temporal lobe epilepsy (De Jong, 1946; De, 1946; Gibbs *et al.*, 1948) but exacerbates certain types of generalized onset seizures (Lennox, 1945; Mustard and Livingston, 1949) |
| Valproic acid | Broad | A broad-spectrum ASM (Marson and Sills, 2015) |
| Vigabatrin | Narrow | May precipitate or exacerbate certain types of generalized onset seizures (Lortie *et al.*, 1993; de Krom *et al.*, 1995; Parker *et al.*, 1998; Mantoan and Walker, 2011) |
| Zonisamide | Broad | A broad-spectrum ASM (Holder and Wilfong, 2011) |

Supplementary Table 5. Effective and ineffective ASMs for CAE or typical absences. Because of the time-intensive nature of literature review required for classifying ASMs into these categories, classification was performed only for ASMs that are found in our datasets of drugs’ effects upon protein function and abundance.

| Drug | Subset | Notes |
| --- | --- | --- |
| Acetazolamide | Effective | Effective (Neufeld, 2016) |
| Carbamazepine | Ineffective | May worsen absence seizures or cause absence status (Somerville, 2009; Kessler and McGinnis, 2019) |
| Chlordiazepoxide | Effective | Benzodiazepines (Hooshmand, 1972; Gram, 1995; Panayiotopoulos, 1999; Vrielynck, 2013) including chlordiazepoxide (Bercel, 1961; Jeavons, 1962; Le Vann, 1962; Watson *et al.*, 1964; Auckland, 1965; Browne and Penry, 1973) are effective |
| Diazepam | Effective | Benzodiazepines are effective (Hooshmand, 1972; Gram, 1995; Panayiotopoulos, 1999; Vrielynck, 2013) |
| Ethosuximide | Effective | Effective (Committee, 2019; Kessler and McGinnis, 2019) |
| Felbamate | Effective | Effective (Devinsky, 1992; Devinsky *et al.*, 1994; French *et al.*, 1999; Pearl and Holmes, 2008) |
| Gabapentin | Ineffective | Ineffective for typical absence seizures (Trudeau *et al.*, 1996; Kessler and McGinnis, 2019) |
| Lamotrigine | Effective | Effective (Committee, 2019; Kessler and McGinnis, 2019) |
| Levetiracetam | Effective | Effective (Committee, 2019; Kessler and McGinnis, 2019) |
| Lorazepam | Effective | Benzodiazepines are effective (Hooshmand, 1972; Gram, 1995; Panayiotopoulos, 1999; Vrielynck, 2013) |
| Midazolam | Effective | Benzodiazepines are effective (Hooshmand, 1972; Gram, 1995; Panayiotopoulos, 1999; Vrielynck, 2013) |
| Oxcarbazepine | Ineffective | May worsen absence seizures or cause absence status (Somerville, 2009; Kessler and McGinnis, 2019) |
| Phensuximide | Effective | A succinimide derivative, like ethosuximide, effective for absence seizures (Millichap, 1952; Doyle *et al.*, 1953; Carter, 1954; Forrer, 1954; Lemere, 1954; Zimmerman, 1954) |
| Phenytoin | Ineffective | May worsen absence seizures or cause absence status (Somerville, 2009; Kessler and McGinnis, 2019) |
| Primidone | Ineffective | Ineffective for typical absence seizures (Michelucci and Pasini, 2015; Committee, 2019) |
| Stiripentol | Effective | Effective (Martinez-Lage, 1984; Loiseau, 1989; Farwell *et al.*, 1993; Trojnar *et al.*, 2005; Keränen, 2015) |
| Tiagabine | Ineffective | May worsen absence seizures or cause absence status (Somerville, 2009; Kessler and McGinnis, 2019) |
| Topiramate | Effective | Effective (Committee, 2019; Kessler and McGinnis, 2019) |
| Trimethadione | Effective | Effective for absence seizures (Lennox, 1945; Butter, 1948, 1952) |
| Valproic acid | Effective | Effective (Committee, 2019; Kessler and McGinnis, 2019) |
| Vigabatrin | Ineffective | May worsen absence seizures or cause absence status (Somerville, 2009; Kessler and McGinnis, 2019) |
| Zonisamide | Effective | Effective (Committee, 2019; Kessler and McGinnis, 2019) |

Supplementary Table 6: Comparing the premise and method underlying the standard approach and our three enhanced approaches.

| Approach | Premise | How a raw score is generated for each drug |
| --- | --- | --- |
| Standard approach | A drug is likely to affect a disease if it affects the function of a protein associated with the disease | No score is generated; only a binary classification is applied, dividing drugs into those that do or do not alter the function of a protein associated with the disease |
| Most disease-associated protein affected | A drug is more likely to affect a disease if it affects the function of a protein more strongly associated with the disease | The raw score for each drug is the inverse GWAS gene-based p-value of the most disease-associated protein it alters in function |
| Disease-association of all proteins affected | A drug is more likely to affect a disease if it affects the function of more proteins more strongly associated with the disease | The raw score for each drug is the cumulative inverse GWAS gene-based p-value of each protein it changes in function |
| Disease-association of all proteins affected and the magnitude of effect | A drug is more likely to affect a disease if it has a stronger effect on the function of more proteins more strongly associated with the disease | The raw score for each drug is the cumulative *boosted* inverse GWAS gene-based p-value of each protein it changes in function, where each inverse GWAS gene-based p-value has been *boosted* in proportion to the affinity of the drug for the protein |

Supplementary Table 7. For all epilepsy, using the top 10% of imputed differentially-expressed genes provides the best drug prioritisation in which the validated drug-set is ranked highest. Using progressively longer or shorter lists leads to a progressive decay in the ranking of validated drugs. For the bottom 30% of imputed differentially-expressed genes, the gene expression changes are too small to make a perceptible difference in the drug prioritisation.

| List-length of genes (%) | Median rank | Median percentile rank |
| --- | --- | --- |
| 5 | 4046.5 | 83 |
| 10 | 3725 | 85 |
| 15 | 5333 | 78 |
| 20 | 5426 | 77 |
| 30 | 7342.5 | 69 |
| 40 | 7619.5 | 68 |
| 50 | 9365 | 61 |
| 60 | 9437.5 | 61 |
| 70 | 10307 | 57 |
| 80 | 10307 | 57 |
| 90 | 10307 | 57 |
| 100 | 10307 | 57 |

Supplementary Table 8. Median ranks of validated drug-sets amongst drug prioritisation made using increasing list-lengths of differentially-expressed genes for different types of epilepsy. Shaded cells indicate the best median rank of the validated drug-set and, hence, the list-length of genes used for the respective type of epilepsy. ND: not done

| Epilepsy type | List-length of imputed differentially-expressed genes | | | | | |
| --- | --- | --- | --- | --- | --- | --- |
|  | 5% | 10% | 15% | 20% | 25% | 30% |
| FE | 5328 | 5221 | 6403 | ND | ND | ND |
| HS | 19551 | 5054 | 6148 | ND | ND | ND |
| GE | 7135.5 | 4694 | 5145.5 | ND | ND | ND |
| JME | 16692.5 | 19581.5 | 6818 | 2655.5 | 6499 | 7045.5 |
| CAE | 7767 | 2154 | 6081 | ND | ND | ND |

Supplementary Table 9. Compounds that were filtered out and the respective reasons.

| Drug | Reason for exclusion |
| --- | --- |
| Metaraminol | Published evidence indicative of blood-brain barrier impermeability (Nelson, 1974; Anton and Berk, 1977) |
| Riluzole | Existing published evidence of efficacy against generalised seizures in animal models (Borowicz *et al.*, 2004; Kim *et al.*, 2007; Jadhav *et al.*, 2016) including DBA/2(De Sarro *et al.*, 2000) |
| Thiocolchicoside | Not safe for long-term human use, according to the European Medicines Agency (<https://www.ema.europa.eu/en/news/european-medicines-agency-recommends-restricting-use-thiocolchicoside-mouth-injection>; accessed 01/04/2020) |
| Ropivacaine | No oral formulation |
| Etomidate | No oral formulation |
| Clomifene | Existing evidence of antiepileptic efficacy in humans (Check *et al.*, 1982; Login and Dreifuss, 1983; Nicoletti *et al.*, 1985; Herzog, 1988a, b) |
| Primaquine | An antimicrobial lacking evidence of safe long-term human use |
| Famciclovir | An antimicrobial lacking evidence of safe long-term human use |
| Propafenone | Not soluble in water or saline |
| Norethisterone | Hormonal medication deemed undesirable for use in males |
| Phentermine | A controlled substance in France |
| Phenazopyridine | Not soluble in water or saline |

Supplementary Table 10: Performance of the standard approach and our three enhanced approaches, measured by the identification and prioritisation of ASMs. AUROC is calculated using drugs’ adjusted scores (see text) from the respective methods. AUROC is computed using the technique of random under-sampling, and presented as mean ± standard deviation (see Supplementary Methods). Prioritisation is calculated using drugs’ ranks, when all drugs have been ranked from highest to lowest predicted effect on the phenotype. Prioritisation result shown is the average (median) rank of ASMs, expressed as a percentile; it is equivalent to the percentage of all drugs ranked below the middle-ranked ASM (see Supplementary Methods). p: permutation-based p-value, after Benjamini-Hochberg correction; AUROC: area under receiver operating characteristic curve; SD: standard deviation; np: not possible; nd: not done.

| Method | Identification of ASMs (mean ± SD of AUROC) | Prioritisation of ASMs (average percentile) | P |
| --- | --- | --- | --- |
| Standard approach | np | np | nd |
| Most disease-associated protein affected | 0.65±0.04 | 67 | 1 x 10^–4^ |
| Disease-association of all proteins affected | 0.67±0.03 | 73 | <1 x 10^–6^ |
| Disease-association of all proteins affected, and the magnitude of effect | 0.74±0.03 | 89 | <1 x 10^–6^ |

Supplementary Table 11. Performance of the FM score, measured by the identification and prioritisation of ASMs. Constituents of the ‘More effective ASMs’ and ‘Less effective ASMs’ drug-sets are specific to each phenotype. ‘Less effective ASMs’ comprise the set of less effective, ineffective, or aggravating ASMs for that phenotype. AUROC is calculated using drugs’ FM scores. AUROC for identifying ASMs from all drugs is computed using the technique of random under-sampling, and presented as mean ± standard deviation. Prioritisation is calculated using drugs’ ranks, when all drugs have been ranked from highest to lowest predicted effect on the phenotype. Prioritisation result shown is the average (median) rank of ASMs, expressed as a percentile; it is equivalent to the percentage of all drugs ranked below the middle-ranked ASM (see Methods). Epi: epilepsy type or syndrome; AUROC: area under the receiver operating characteristics; SD: standard deviation; p: permutation-based p-value after Benjamini–Hochberg correction; CAE: childhood absence epilepsy; GE: generalized epilepsy; HS: focal epilepsy with hippocampal sclerosis; JME: juvenile myoclonic epilepsy.

| Epi | Prioritisation | | AUROC | | |
| --- | --- | --- | --- | --- | --- |
|  | More effective ASMs | Less effective ASMs | More effective ASMs  from all drugs | Less effective ASMs  from all drugs | More from less  effective ASMs |
| HS | 69 | 32 | 0.63±0.14 | 0.34±0.14 | 0.8 |
| GE | 95 | 97 | 0.82±0.03 | 0.78±0.06 | 0.51 |
| JME | 92 | 90 | 0.83±0.05 | 0.73±0.08 | 0.67 |
| CAE | 84 | 59 | 0.64±0.06 | 0.52±0.12 | 0.65 |

Supplementary Table 12. Performance of the AC score, measured by the identification and prioritisation of ASMs. Constituents of the ‘More effective ASMs’ and ‘Less effective ASMs’ drug-sets are specific to each phenotype. ‘Less effective ASMs’ comprise the set of less effective, ineffective, or aggravating ASMs for that phenotype. AUROC is calculated using drugs’ AC scores. AUROC for identifying ASMs from all drugs is computed using the technique of random under-sampling, and presented as mean ± standard deviation. Prioritisation is calculated using drugs’ ranks, when all drugs have been ranked from highest to lowest predicted effect on the phenotype. Prioritisation result shown is the average (median) rank of ASMs, expressed as a percentile; it is equivalent to the percentage of all drugs ranked below the middle-ranked ASM (see Methods). Epi: epilepsy type or syndrome; AUROC: area under the receiver operating characteristics; SD: standard deviation; p: permutation-based p-value after Benjamini–Hochberg correction; CAE: childhood absence epilepsy; GE: generalized epilepsy; HS: focal epilepsy with hippocampal sclerosis; JME: juvenile myoclonic epilepsy.

| Epi | Prioritisation | | AUROC | | |
| --- | --- | --- | --- | --- | --- |
|  | More effective ASMs | Less effective ASMs | More effective ASMs  from all drugs | Less effective ASMs  from all drugs | More from less  effective ASMs |
| HS | 79 | 33 | 0.62±0.10 | 0.48±0.18 | 0.6 |
| GE | 80 | 48 | 0.69±0.07 | 0.43±0.09 | 0.72 |
| JME | 89 | 57 | 0.73±0.06 | 0.49±0.07 | 0.73 |
| CAE | 91 | 34 | 0.71±0.05 | 0.38±0.12 | 0.77 |

Supplementary Figure 1. For all epilepsy, using the top 10% of imputed differentially-expressed genes provides the best drug prioritisation in which the validated drug-set is ranked highest. Using progressively longer or shorter lists leads to a progressive decay in the ranking of validated drugs. For the bottom 30% of imputed differentially-expressed genes, the gene expression changes are too small to make a perceptible difference in the drug prioritisation.

Supplementary Figure 2. Results of the focal epilepsy arm of the SANAD trial. Data for 95% confidence intervals is shown. OXC: oxcarbazepine; LTG: lamotrigine; TPM: topiramate; GBP: gabapentin

# The International League Against Epilepsy Consortium on Complex Epilepsies author names

*Members listed in alphabetical order:*

Bassel Abou-Khalil^1^, Pauls Auce^2, 3^, Andreja Avbersek^4^, Melanie Bahlo^5-7^, David J Balding^8, 9^, Thomas Bast^10, 11^, Larry Baum^12-14^, Albert J Becker^15^, Felicitas Becker^16, 17^ Bianca Berghuis^18^, Samuel F Berkovic^19^, Katja E Boysen^19^, Jonathan P Bradfield^20, 21^, Lawrence C Brody^22^, Russell J Buono^20, 23, 24^, Ellen Campbell^25^, Gregory D Cascino^26^, Claudia B Catarino^4^, Gianpiero L Cavalleri^27, 28^, Stacey S Cherny^13, 29^, Krishna Chinthapalli^4^, Alison J Coffey^30^, Alastair Compston^31^, Antonietta Coppola^32, 33^, Patrick Cossette^34^, John J Craig^35^, Gerrit-Jan de Haan^36^, Peter De Jonghe^37, 38^, Carolien G F de Kovel^39^, Norman Delanty^27, 28, 40^, Chantal Depondt^41^, Orrin Devinsky^42^, Dennis J Dlugos^43^, Colin P Doherty^28, 44^, Christian E Elger^45^, Johan G Eriksson^46^, Thomas N Ferraro^23, 47^, Martha Feucht^48^, Ben Francis^49^, Andre Franke^50^, Jacqueline A French^51^, Saskia Freytag^5^, Verena Gaus^52^, Eric B Geller^53^, Christian Gieger^54, 55^, Tracy Glauser^56^, Simon Glynn^57^, David B Goldstein^58, 59^, Hongsheng Gui^13^, Youling Guo^13^, Kevin F Haas^1^, Hakon Hakonarson^20, 60^, Kerstin Hallmann^45, 61^, Sheryl Haut^62^, Erin L Heinzen^58, 59^, Ingo Helbig^43, 63^, Christian Hengsbach^16^, Helle Hjalgrim^64, 65^, Michele Iacomino^33^, Andrés Ingason^66^, Jennifer Jamnadas-Khoda^4, 67^, Michael R Johnson^68^, Reetta Kälviäinen^69, 70^, Anne-Mari Kantanen^69^, Dalia Kasperavičiūte^4^, Dorothee Kasteleijn-Nolst Trenite^39^, Heidi E Kirsch^71^, Robert C Knowlton^72^, Bobby P C Koeleman^39^, Roland Krause^73^, Martin Krenn^74^, Wolfram S Kunz^45^, Ruben Kuzniecky^75^, Patrick Kwan^12, 76, 77^, Dennis Lal^78^, Yu-Lung Lau^79^, Holger Lerche^16^, Costin Leu^4, 78, 80^, Wolfgang Lieb^81^, Dick Lindhout^36, 39^, Warren D Lo^82^, Iscia Lopes-Cendes^83, 84^, Daniel H Lowenstein^71^, Alberto Malovini^85^, Anthony G Marson^2^, Thomas Mayer^86^, Mark McCormack^27^, James L Mills^87^, Nasir Mirza^2^, Martina Moerzinger^48^, Rikke S Møller^64, 65^, Anne M Molloy^88^, Hiltrud Muhle^63^, Mark Newton^89^, Ping-Wing Ng^90^, Markus M Nöthen^91^, Peter Nürnberg^92^, Terence J O’Brien^76, 77^, Karen L Oliver^19^, Aarno Palotie^93, 94^, Faith Pangilinan^22^, Sarah Peter^73^, Slavé Petrovski^76, 95^, Annapurna Poduri^96^, Michael Privitera^97^, Rodney Radtke^98^, Sarah Rau^16^, Philipp S Reif^99, 100^, Eva M Reinthaler^74^, Felix Rosenow^99, 100^, Josemir W Sander^4, 36, 101^, Thomas Sander^52, 92^, Theresa Scattergood^102^, Steven C Schachter^103^, Christoph J Schankin^104^, Ingrid E Scheffer^19, 105^, Bettina Schmitz^52^, Susanne Schoch^15^, Pak C Sham^13^, Jerry J Shih^106^, Graeme J Sills^2^, Sanjay M Sisodiya^4, 101^, Lisa Slattery^107^, Alexander Smith^78^, David F Smith^3^, Michael C Smith^108^, Philip E Smith^109^, Anja C M Sonsma^39^, Doug Speed^8,110^, Michael R Sperling^111^, Bernhard J Steinhoff^10^, Ulrich Stephani^63^, Remi Stevelink^39^, Konstantin Strauch^112, 113^, Pasquale Striano^114^, Hans Stroink^115^, Rainer Surges^45^, K Meng Tan^76^, Liu Lin Thio^116^, G Neil Thomas^117^, Marian Todaro^76^, Rossana Tozzi^118^, Maria S Vari^114^, Eileen P G Vining^119^, Frank Visscher^120^, Sarah von Spiczak^63^, Nicole M Walley^58,121^, Yvonne G Weber^122^, Zhi Wei^123^, Judith Weisenberg^116^, Christopher D Whelan^27^, Peter Widdess-Walsh^27,28,40^, Markus Wolff^124^, Stefan Wolking^16^, Wanling Yang^79^, Federico Zara^33^, Fritz Zimprich^74^

1. Vanderbilt University Medical Center, Nashville, TN 37232, USA.

2. Department of Pharmacology & Therapeutics, University of Liverpool, Liverpool L69 3GL, UK.

3. The Walton Centre NHS Foundation Trust, Liverpool L9 7LJ, UK.

4. Department of Clinical and Experimental Epilepsy, UCL Institute of Neurology, Queen Square, London WC1N 3BG, UK.

5. Population Health and Immunity Division, The Walter and Eliza Hall Institute of Medical Research, Parkville 3052, Australia.

6. Department of Biology, University of Melbourne, Parkville 3010, Australia.

7. School of Mathematics and Statistics, University of Melbourne, Parkville 3010, Australia.

8. UCL Genetics Institute, University College London, London WC1E 6BT, UK.

9. Melbourne Integrative Genomics, University of Melbourne, Parkville 3052, Australia.

10. Epilepsy Center Kork, Kehl-Kork 77694, Germany.

11. Medical Faculty of the University of Freiburg, Freiburg 79085, Germany.

12. Centre for Genomic Sciences, The University of Hong Kong, Hong Kong.

13. Department of Psychiatry, The University of Hong Kong, Hong Kong.

14. The State Key Laboratory of Brain and Cognitive Sciences, University of Hong Kong, Hong Kong, China.

15. Section for Translational Epilepsy Research, Department of Neuropathology, University of Bonn Medical Center, Bonn 53105, Germany.

16. Department of Neurology and Epileptology, Hertie Institute for Clinical Brain Research, University of Tübingen, Tübingen 72076, Germany.

17. Department of Neurology, University of Ulm, Ulm 89081, Germany.

18. Stichting Epilepsie Instellingen Nederland (SEIN), Zwolle 8025 BV, The Netherlands.

19. Epilepsy Research Centre, University of Melbourne, Austin Health, Heidelberg 3084, Australia.

20. Center for Applied Genomics, The Children's Hospital of Philadelphia, Philadelphia, PA 19104, USA.

21. Quantinuum Research LLC, San Diego, CA 92101, USA.

22. National Human Genome Research Institute, National Institutes of Health, Bethesda, MD 20892, USA.

23. Department of Biomedical Sciences, Cooper Medical School of Rowan University Camden, NJ 08103, USA.

24. Department of Neurology, Thomas Jefferson University Hospital, Philadelphia, PA 19107, USA.

25. Belfast Health and Social Care Trust, Belfast BT9 7AB, UK.

26. Division of Epilepsy, Department of Neurology, Mayo Clinic, Rochester, MN 55902, USA.

27. Department of Molecular and Cellular Therapeutics, The Royal College of Surgeons in Ireland, Dublin 2, Ireland.

28. The FutureNeuro Research Centre, Dublin 2, Ireland.

29. Department of Epidemiology and Preventive Medicine, School of Public Health, Sackler Faculty of Medicine, Tel Aviv University, Tel Aviv 6997801, Israel.

30. The Wellcome Trust Sanger Institute, Hinxton, Cambridge CB10 1SA, UK.

31. Department of Clinical Neurosciences, Cambridge Biomedical Campus, Cambridge CB2 0SL, UK.

32. Department of Neuroscience, Reproductive and Odontostomatological Sciences, University Federico II, Naples 80138, Italy.

33. Laboratory of Neurogenetics and Neurosciences, Institute G. Gaslini, Genova 16148, Italy.

34. Department of Neurosciences, Université de Montréal, Montréal, CA 26758, Canada.

35. Department of Neurology, Royal Victoria Hospital, Belfast Health and Social Care Trust, Grosvenor Road, Belfast BT12 6BA, UK.

36. Stichting Epilepsie Instellingen Nederland (SEIN), Heemstede 2103 SW, The Netherlands.

37. Neurogenetics Group, Center for Molecular Neurology, VIB and Laboratory of Neurogenetics, Institute Born-Bunge, University of Antwerp, Antwerp 2610, Belgium.

38. Department of Neurology, Antwerp University Hospital, Edegem 2650, Belgium.

39. Department of Genetics, University Medical Center Utrecht, Utrecht 3584 CX, The Netherlands.

40. Division of Neurology, Beaumont Hospital, Dublin D09 FT51, Ireland.

41. Department of Neurology, Hôpital Erasme, Université Libre de Bruxelles, Bruxelles 1070, Belgium.

42. Comprehensive Epilepsy Center, New York University School of Medicine, New York, NY 10016, USA.

43. Department of Neurology, The Children's Hospital of Philadelphia, Philadelphia, PA 19104, USA.

44. Neurology Department, St. James’s Hospital, Dublin D03 VX82, Ireland.

45. Department of Epileptology, University of Bonn Medical Centre, Bonn 53127, Germany.

46. Department of General Practice and Primary Health Care, University of Helsinki and Helsinki University Hospital, Helsinki 0014, Finland.

47. Department of Pharmacology and Psychiatry, University of Pennsylvania Perlman School of Medicine, Philadelphia, PA 19104, USA.

48. Department of Pediatrics and Neonatology, Medical University of Vienna, Vienna 1090, Austria.

49. Department of Biostatistics, University of Liverpool, Liverpool L69 3GL, UK.

50. Institute of Clinical Molecular Biology, Christian-Albrechts-University of Kiel, University Hospital Schleswig Holstein, Kiel 24105, Germany.

51. Department of Neurology, NYU School of Medicine, New York City, NY 10003, USA.

52. Department of Neurology, Charité Universitaetsmedizin Berlin, Campus Virchow-Clinic, Berlin 13353, Germany.

53. Institute of Neurology and Neurosurgery at St. Barnabas, Livingston, NJ 07039, USA.

54. Research Unit of Molecular Epidemiology, Helmholtz Zentrum München - German Research Center for Environmental Health, Neuherberg D-85764, Germany.

55. Institute of Epidemiology, Helmholtz Zentrum München - German Research Center for Environmental Health, Neuherberg D-85764, Germany.

56. Comprehensive Epilepsy Center, Division of Neurology, Cincinnati Children's Hospital Medical Center, Cincinnati, OH 45229, USA.

57. Department of Neurology, University of Michigan, Ann Arbor, MI 48109, USA.

58. Center for Human Genome Variation, Duke University School of Medicine, Durham, NC 27710, USA.

59. Institute for Genomic Medicine, Columbia University Medical Center, New York, NY 10032, USA.

60. Division of Human Genetics, Department of Pediatrics, The Perelman School of Medicine, University of Pennsylvania, Philadelphia, PA 19104, USA.

61. Life and Brain Center, University of Bonn Medical Center, Bonn 53127, Germany.

62. Montefiore Medical Center, Bronx, NY 10467, USA.

63. Department of Neuropediatrics, University Medical Center Schleswig-Holstein (UKSH), Kiel 24105, Germany.

64. Danish Epilepsy Centre, Dianalund 4293, Denmark.

65. Institute of Regional Health Services Research, University of Southern Denmark, Odense 5000, Denmark.

66. deCODE genetics, Reykjavik IS-101, Iceland.

67. Department of Psychiatry and Applied Psychology, Institute of Mental Health University of Nottingham, Nottingham NG7 2TU, UK.

68. Faculty of Medicine, Imperial College London, London SW7 2AZ, UK.

69. Kuopio Epilepsy Center, Neurocenter, Kuopio University Hospital, Kuopio 70029, Finland.

70. Institute of Clinical Medicine, University of Eastern Finland, Kuopio 70029, Finland.

71. Department of Neurology, University of California, San Francisco, CA 94143, USA.

72. University of Alabama Birmingham, Department of Neurology, Birmingham, AL 35233, USA.

73. Luxembourg Centre for Systems Biomedicine, University of Luxembourg, Esch-sur-Alzette L-4362, Luxembourg.

74. Department of Neurology, Medical University of Vienna, Vienna 1090, Austria.

75. Department of Neurology, Zucker-Hofstra Northwell School of Medicine, NY 10075, USA.

76. Department of Medicine, University of Melbourne, Royal Melbourne Hospital, Parkville 3050, Australia.

77. Department of Neuroscience, Central Clinical School, Monash University, Melbourne 3004, Australia.

78. Stanley Center for Psychiatric Research, Broad Institute of Harvard and M.I.T., Cambridge, MA 02142, USA.

79. Department of Paediatrics and Adolescent Medicine, The University of Hong Kong, Hong Kong.

80. Genomic Medicine Institute, Lerner Research Institute, Cleveland Clinic, Cleveland, OH 44195, USA.

81. Institut für Epidemiologie Christian-Albrechts-Universität zu Kiel, Kiel 24105, Germany.

82. Department of Pediatrics and Neurology, Ohio State University and Nationwide Children's Hospital, Columbus, OH 43205, USA.

83. Department of Medical Genetics, School of Medical Sciences, University of Campinas (UNICAMP), Campinas, SP 13083-887, Brazil.

84. Brazilian Institute of Neuroscience and Neurotechnology (BRAINN), Campinas, SP 13083-970, Brazil.

85. Istituti Clinici Scientifici Maugeri, Pavia 27100, Italy.

86. Epilepsy Center Kleinwachau, Radeberg 01454, Germany.

87. Division of Intramural Population Health Research, Eunice Kennedy Shriver National Institute of Child Health and Human Development, National Institutes of Health, Bethesda, MD 20892, USA*.*

88. School of Medicine, Trinity College Dublin, Dublin 2, Ireland.

89. Department of Neurology, Austin Health, Heidelberg 3084, Australia.

90. United Christian Hospital, Hong Kong.

91. Institute of Human Genetics, University of Bonn Medical Center, Bonn 53127, Germany.

92. Cologne Center for Genomics, University of Cologne, Cologne 50931, Germany.

93. Institute for Molecular Medicine Finland (FIMM), University of Helsinki, Helsinki 0014, Finland*.*

94. The Broad Institute of M.I.T. and Harvard, Cambridge, MA 02142, USA.

95. AstraZeneca Centre for Genomics Research, Precision Medicine and Genomics, IMED Biotech Unit, AstraZeneca, Cambridge CB2 0AA, UK.

96. Department of Neurology, Boston Children's Hospital, Harvard Medical School, Boston, MA 02115, USA.

97. Department of Neurology, Neuroscience Institute, University of Cincinnati Medical Center, Cincinnati, OH 45220, USA.

98. Department of Neurology, Duke University School of Medicine, Durham, NC 27710, USA.

99. Epilepsy-Center Hessen, Department of Neurology, University Medical Center Giessen and Marburg, Marburg, Germany and Philipps-University Marburg, Marburg 35043, Germany.

100. Epilepsy Center Frankfurt Rhine-Main, Center of Neurology and Neurosurgery, University Hospital Frankfurt and LOEWE Center for Personalized Translational Epilepsy Research (CePTER), Goethe University Frankfurt, Frankfurt 60528, Germany.

101. Chalfont Centre for Epilepsy, Chalfont-St-Peter, Buckinghamshire SL9 0RJ, UK.

102. Department of Endocrinology, Hospital of The University of Pennsylvania, Philadelphia, PA 19104, USA.

103. Departments of Neurology, Beth Israel Deaconess Medical Center, Massachusetts General Hospital, and Harvard Medical School, Boston, MA 02215, USA.

104. Department of Neurology, Inselspital, Bern University Hospital, University of Bern, Bern 3010, Switzerland.

105. Department of Neurology, Royal Children's Hospital, Parkville 3052, Australia.

106. Department of Neurosciences, University of California, San Diego, La Jolla, CA 92037, USA.

107. The Royal College of Surgeons in Ireland, Dublin D02 YN77, Ireland.

108. Rush University Medical Center, Chicago, IL 60612, USA.

109. Department of Neurology, Alan Richens Epilepsy Unit, University Hospital of Wales, Cardiff CF14 4XW, UK.

110. Aarhus Institute of Advanced Studies (AIAS), Aarhus University, 8000 Aarhus, Denmark.

111. Department of Neurology and Comprehensive Epilepsy Center, Thomas Jefferson University, Philadelphia, PA 19107, USA.

112. Institute of Genetic Epidemiology, Helmholtz Zentrum München - German Research Center for Environmental Health, Neuherberg D-85764, Germany.

113. Chair of Genetic Epidemiology, IBE, Faculty of Medicine, LMU Munich 80539, Germany.

114. Pediatric Neurology and Muscular Diseases Unit, Department of Neurosciences, Rehabilitation, Ophthalmology, Genetics, Maternal and Child Health, G. Gaslini Institute, University of Genoa, Genova 16148, Italy.

115. CWZ Hospital, 6532 SZ Nijmegen, The Netherlands.

116. Department of Neurology, Washington University School of Medicine, St. Louis, MO 63110, USA*.*

117. Institute for Applied Health Research, University of Birmingham, Birmingham B15 2TT, UK.

118. C. Mondino National Neurological Institute, Pavia 27100, Italy.

119. Departments of Neurology and Pediatrics, The Johns Hopkins University School of Medicine, Baltimore, MD 21287, USA.

120. Department of Neurology, Admiraal De Ruyter Hospital, Goes 4462, The Netherlands.

121. Division of Medical Genetics, Department of Pediatrics, Duke University Medical Center, Durham, NC 27710, USA.

122. Department of Neurology and Epileptology, University of Aachen, Aachen 52074, Germany.

123. Department of Computer Science, New Jersey Institute of Technology, NJ 07102, USA.

124. Department of Pediatric Neurology, Vivantes Hospital Neukölln, 12351 Berlin, Germany.

# Supplementary References

Ahmad M, Abu-Taweel GM, Aboshaiqah AE, Ajarem JS. The effects of quinacrine, proglumide, and pentoxifylline on seizure activity, cognitive deficit, and oxidative stress in rat lithium-pilocarpine model of status epilepticus. Oxid Med Cell Longev 2014; 2014: 630509.

Akarsu ES, Ayhan IH. Effects of intracerebral iloprost injections on motor activity and chemically-induced seizures in rats. Methods Find Exp Clin Pharmacol 1992; 14(7): 517-22.

Akhundov RA, Dzhafarova SA, Aliev AN. [The search for new anticonvulsant agents based on nicotinamide]. Eksp Klin Farmakol 1992; 55(1): 27-9.

al-Tajir G, Starr MS. D-2 agonists protect rodents against pilocarpine-induced convulsions by stimulating D-2 receptors in the striatum, but not in the substantia nigra. Pharmacol Biochem Behav 1991; 39(1): 109-13.

Aley KO, Kulkarni SK. Altered response to GABAergic agents following electro and chemo convulsions in mice. Indian J Exp Biol 1991; 29(3): 241-3.

Amabeoku G. Involvement of GABAergic mechanisms in chloroquine-induced seizures in mice. Gen Pharmacol 1992; 23(2): 225-9.

Amabeoku G, Chandomba R. Strychnine-induced seizures in mice: the role of noradrenaline. Prog Neuropsychopharmacol Biol Psychiatry 1994; 18(4): 753-63.

Amabeoku GJ. Gamma-aminobutyric acid and glutamic acid receptors may mediate theophylline-induced seizures in mice. Gen Pharmacol 1999; 32(3): 365-72.

Amabeoku GJ, Chikuni O. Effects of some GABAergic agents on quinine-induced seizures in mice. Experientia 1992; 48(7): 659-62.

Amador A, Bostick CD, Olson H, Peters J, Camp CR, Krizay D, et al. Modelling and treating GRIN2A developmental and epileptic encephalopathy in mice. Brain 2020; 143(7): 2039-57.

Amand J, Fehlmann T, Backes C, Keller A. DynaVenn: web-based computation of the most significant overlap between ordered sets. BMC Bioinformatics 2019; 20(1): 743.

Aminiahidashti H, Laali A, Nosrati N, Jahani F. Recurrent seizures after lidocaine ingestion. J Adv Pharm Technol Res 2015; 6(1): 35-7.

Anighoro A, Bajorath J, Rastelli G. Polypharmacology: challenges and opportunities in drug discovery. J Med Chem 2014; 57(19): 7874-87.

Anton AH, Berk AI. Distribution of metaraminol and its relation to norepinephrine. Eur J Pharmacol 1977; 44(2): 161-7.

Apland JP, Cann FJ. Anticonvulsant effects of memantine and MK-801 in guinea pig hippocampal slices. Brain Res Bull 1995; 37(3): 311-6.

Asconape J, Diedrich A, DellaBadia J. Myoclonus associated with the use of gabapentin. Epilepsia 2000; 41(4): 479-81.

Atakli D, Sozuer D, Atay T, Baybas S, Arpaci B. Misdiagnosis and treatment in juvenile myoclonic epilepsy. Seizure 1998; 7(1): 63-6.

Auckland NL. Drugs for petit mal. Can Med Assoc J 1965; 93(13): 707-8.

Banach M, Piskorska B, Borowicz-Reutt KK. Propafenone enhances the anticonvulsant action of classical antiepileptic drugs in the mouse maximal electroshock model. Pharmacol Rep 2016; 68(3): 555-60.

Bercel N. Chlordiazepoxide (Librium) as an anti-convulsant. DISEASES OF THE NERVOUS SYSTEM 1961; 22(7): 17-&.

Biraben A, Allain H, Scarabin JM, Schuck S, Edan G. Exacerbation of juvenile myoclonic epilepsy with lamotrigine. Neurology 2000; 55(11): 1758.

Bolognesi ML. Polypharmacology in a single drug: multitarget drugs. Curr Med Chem 2013; 20(13): 1639-45.

Bornigen D, Tranchevent LC, Bonachela-Capdevila F, Devriendt K, De Moor B, De Causmaecker P*, et al.* An unbiased evaluation of gene prioritization tools. Bioinformatics 2012; 28(23): 3081-8.

Borowicz KK, Banach M. Antiarrhythmic drugs and epilepsy. Pharmacol Rep 2014; 66(4): 545-51.

Borowicz KK, Sekowski A, Drelewska E, Czuczwar SJ. Riluzole enhances the anti-seizure action of conventional antiepileptic drugs against pentetrazole-induced convulsions in mice. Pol J Pharmacol 2004; 56(2): 187-93.

Bouchet C, Spyratos F, Hacene K, Durcos L, Becette V, Oglobine J. Prognostic value of urokinase plasminogen activator in primary breast carcinoma: comparison of two immunoassay methods. Br J Cancer 1998; 77(9): 1495-501.

Bourgeois BF, Dodson WE, Ferrendelli JA. Potentiation of the antiepileptic activity of phenobarbital by nicotinamide. Epilepsia 1983; 24(2): 238-44.

Bouwman NA, Morre HH. Lidocaine-induced seizure during carotid endarterectomy. Clin Neurophysiol 2013; 124(7): 1481-3.

Braslavskii VE, Shchavelev VA, Kryzhanovskii GN, Nikushkin EV, Germanov SB. [Effect of nicotinamide on focal and generalized epileptic activity in the cerebral cortex]. Biull Eksp Biol Med 1982; 94(8): 39-42.

Brodie MJ. Antiepileptic drug therapy the story so far. Seizure 2010; 19(10): 650-5.

Brown AS, Kong SW, Kohane IS, Patel CJ. ksRepo: a generalized platform for computational drug repositioning. BMC Bioinformatics 2016a; 17: 78.

Brown AS, Patel CJ. MeSHDD: Literature-based drug-drug similarity for drug repositioning. J Am Med Inform Assoc 2017a; 24(3): 614-8.

Brown AS, Patel CJ. A standard database for drug repositioning. Sci Data 2017b; 4: 170029.

Brown JW, Moeller A, Schmidt M, Turner SC, Nimmrich V, Ma J*, et al.* Anticonvulsant effects of structurally diverse GABA(B) positive allosteric modulators in the DBA/2J audiogenic seizure test: Comparison to baclofen and utility as a pharmacodynamic screening model. Neuropharmacology 2016b; 101: 358-69.

Browne TR, Penry JK. Benzodiazepines in the treatment of epilepsy. A review. Epilepsia 1973; 14(3): 277-310.

Butter AJ. Tridione in the treatment of petit mal. Br Med J 1948; 1(4539): 13.

Butter AJ. Tridione compared with malidone in the treatment of petit mal. J Neurol Neurosurg Psychiatry 1952; 15(1): 37-8.

Cai X, Chen Y, Gao Z, Xu R. Explore Small Molecule-induced Genome-wide Transcriptional Profiles for Novel Inflammatory Bowel Disease Drug. AMIA Jt Summits Transl Sci Proc 2016; 2016: 22-31.

Cakil D, Yildirim M, Ayyildiz M, Agar E. The effect of co-administration of the NMDA blocker with agonist and antagonist of CB1-receptor on penicillin-induced epileptiform activity in rats. Epilepsy Res 2011; 93(2-3): 128-37.

Cao DS, Liu S, Xu QS, Lu HM, Huang JH, Hu QN*, et al.* Large-scale prediction of drug-target interactions using protein sequences and drug topological structures. Anal Chim Acta 2012; 752: 1-10.

Carrazana EJ, Wheeler SD. Exacerbation of juvenile myoclonic epilepsy with lamotrigine. Neurology 2001; 56(10): 1424-5.

Carter CH. Use of milontin in the control of petit mal epilepsy. Neurology 1954; 4(12): 935-7.

Cella D, Nichol MB, Eton D, Nelson JB, Mulani P. Estimating clinically meaningful changes for the Functional Assessment of Cancer Therapy--Prostate: results from a clinical trial of patients with metastatic hormone-refractory prostate cancer. Value Health 2009; 12(1): 124-9.

Chalkidou A, O'Doherty MJ, Marsden PK. False Discovery Rates in PET and CT Studies with Texture Features: A Systematic Review. PLoS One 2015; 10(5): e0124165.

Check JH, Lublin FD, Mandel MM. Clomiphene as an anticonvulsant drug: A case report. Arch Neurol 1982; 39(12): 784.

Chen L, Chan YS, Yung WH. GABA-B receptor activation in the rat globus pallidus potently suppresses pentylenetetrazol-induced tonic seizures. J Biomed Sci 2004; 11(4): 457-64.

Chen Y, Cai X, Xu R. Combining Human Disease Genetics and Mouse Model Phenotypes towards Drug Repositioning for Parkinson's disease. AMIA Annu Symp Proc 2015; 2015: 1851-60.

Chen Y, Gao Z, Wang B, Xu R. Towards precision medicine-based therapies for glioblastoma: interrogating human disease genomics and mouse phenotypes. BMC Genomics 2016; 17 Suppl 7: 516.

Chen Y, Xu R. Drug repurposing for glioblastoma based on molecular subtypes. J Biomed Inform 2016; 64: 131-8.

Cheng F, Desai RJ, Handy DE, Wang R, Schneeweiss S, Barabasi AL*, et al.* Network-based approach to prediction and population-based validation of in silico drug repurposing. Nat Commun 2018; 9(1): 2691.

Cheng F, Kovacs IA, Barabasi AL. Network-based prediction of drug combinations. Nat Commun 2019; 10(1): 1197.

Chiang AP, Butte AJ. Systematic evaluation of drug-disease relationships to identify leads for novel drug uses. Clin Pharmacol Ther 2009; 86(5): 507-10.

Cho KT, Hong SK. Myoclonus induced by the use of gabapentin. J Korean Neurosurg Soc 2008; 43(5): 237-8.

Chong DJ, French JA. Levetiracetam. The Treatment of Epilepsy 2015: 516-32.

Colomer R, Aparicio J, Montero S, Guzman C, Larrodera L, Cortes-Funes H. Low levels of basic fibroblast growth factor (bFGF) are associated with a poor prognosis in human breast carcinoma. Br J Cancer 1997; 76(9): 1215-20.

Committee JF. BNF 77 (British National Formulary) March 2019: Pharmaceutical Press; 2019.

Consortium GT. Human genomics. The Genotype-Tissue Expression (GTEx) pilot analysis: multitissue gene regulation in humans. Science 2015; 348(6235): 648-60.

Consortium GT, Laboratory DA, Coordinating Center -Analysis Working G, Statistical Methods groups-Analysis Working G, Enhancing Gg, Fund NIHC, et al. Genetic effects on gene expression across human tissues. Nature 2017; 550(7675): 204-13.

Crespel A, Genton P, Berramdane M, Coubes P, Monicard C, Baldy-Moulinier M*, et al.* Lamotrigine associated with exacerbation or de novo myoclonus in idiopathic generalized epilepsies. Neurology 2005; 65(5): 762-4.

Cross JH, Riney CJ. Topiramate. The Treatment of Epilepsy 2015: 642-51.

Dam M. Recent advances in the treatment of epilepsy. Acta Neurol Scand Suppl 1980; 78: 88-102.

De Jong RN. Further observations on the use of tridione in the control of psychomotor attacks. Am J Psychiatry 1946; 103(2): 162-4.

De JR. Effect of tridione in the control of psychomotor attacks. J Am Med Assoc 1946; 130: 565-7.

de Krom MC, Verduin N, Visser E, Kleijer M, Scholtes F, De Groen JH. Status epilepticus during vigabatrin treatment: a report of three cases. Seizure 1995; 4(2): 159-62.

De Sarro G, Siniscalchi A, Ferreri G, Gallelli L, De Sarro A. NMDA and AMPA/kainate receptors are involved in the anticonvulsant activity of riluzole in DBA/2 mice. Eur J Pharmacol 2000; 408(1): 25-34.

DeGiorgio CM, Miller PR, Harper R, Gornbein J, Schrader L, Soss J*, et al.* Fish oil (n-3 fatty acids) in drug resistant epilepsy: a randomised placebo-controlled crossover study. J Neurol Neurosurg Psychiatry 2015; 86(1): 65-70.

Deng S, Luo R, Mao M, Huang L. Myoclonus precipitated by oral suspension of oxcarbazepine in idiopathic generalized epilepsy. Int J Clin Pharmacol Ther 2012; 50(4): 300-1.

Desai A, Kherallah Y, Szabo C, Marawar R. Gabapentin or pregabalin induced myoclonus: A case series and literature review. J Clin Neurosci 2019; 61: 225-34.

DeToledo JC. Lidocaine and seizures. Ther Drug Monit 2000; 22(3): 320-2.

DeToledo JC, Minagar A, Lowe MR. Lidocaine-induced seizures in patients with history of epilepsy: effect of antiepileptic drugs. Anesthesiology 2002; 97(3): 737-9.

Devinsky O. Felbamate for absence seizures. Epilepsia 1992; 33(3): 84.

Devinsky O, Kothari M, Savino L, Luciano D. Felbamate for refractory absence seizures. Journal of Epilepsy 1994; 7(3): 189-94.

Diouf M, Bonnetain F, Barbare JC, Bouche O, Dahan L, Paoletti X*, et al.* Optimal cut points for quality of life questionnaire-core 30 (QLQ-C30) scales: utility for clinical trials and updates of prognostic systems in advanced hepatocellular carcinoma. Oncologist 2015; 20(1): 62-71.

Dogan E, Aygun H, Arslan G, Rzayev E, Avci B, Ayyildiz M, et al. The Role of NMDA Receptors in the Effect of Purinergic P2X7 Receptor on Spontaneous Seizure Activity in WAG/Rij Rats With Genetic Absence Epilepsy. Front Neurosci 2020; 14: 414.

Donner Y, Kazmierczak S, Fortney K. Drug Repurposing Using Deep Embeddings of Gene Expression Profiles. Mol Pharm 2018; 15(10): 4314-25.

Dorf E, Kuntz AF, Kelsey J, Holstege CP. Lidocaine-induced altered mental status and seizure after hematoma block. J Emerg Med 2006; 31(3): 251-3.

Doyle PJ, Livingston S, Pearson PH. Use of milontin in the treatment of petit mal epilepsy (three per second spike and wave dysrhythmia). J Pediatr 1953; 43(2): 164-6.

Dunkel IJ, Shi W, Salvaggio K, Marr BP, Brodie SE, Gobin YP*, et al.* Risk factors for severe neutropenia following intra-arterial chemotherapy for intra-ocular retinoblastoma. PLoS One 2014; 9(10): e108692.

Durmuller N, Smith SE, Meldrum BS. Proconvulsant and anticonvulsant effects of Evans blue dye in rodents. Neuroreport 1993; 4(6): 683-6.

Dzoljic MR, v d Poel-Heisterkamp AL. The effects of GABA-ergic drugs on enkephalin-induced motor seizure phenomena in the rat. Clin Exp Pharmacol Physiol 1981; 8(2): 141-50.

Eldridge R, Iivanainen M, Stern R, Koerber T, Wilder BJ. "Baltic" myoclonus epilepsy: hereditary disorder of childhood made worse by phenytoin. Lancet 1983; 2(8354): 838-42.

Enginar N, Yamanturk P, Nurten A, Koyuncuoglu H. Scopolamine-induced convulsions in food given fasted mice: effects of clonidine and tizanidine. Epilepsy Res 1999; 35(2): 155-60.

Enoki H, Hata H, Ohmori I, Maniwa S, Ohta H, Kobayashi K. [Clinical applications and the effect of mexiletine on refractory epilepsies]. No To Hattatsu 2000; 32(1): 29-34.

Faingold CL, Marcinczyk MJ, Casebeer DJ, Randall ME, Arneric SP, Browning RA. GABA in the inferior colliculus plays a critical role in control of audiogenic seizures. Brain Res 1994; 640(1-2): 40-7.

Fanella M, Egeo G, Fattouch J, Casciato S, Lapenta L, Morano A*, et al.* Oxcarbazepine-induced myoclonic status epilepticus in juvenile myoclonic epilepsy. Epileptic Disord 2013; 15(2): 181-7.

Fang H, Gough J. A disease-drug-phenotype matrix inferred by walking on a functional domain network. Mol Biosyst 2013; 9(7): 1686-96.

Farwell JR, Anderson GD, Kerr BM, Tor JA, Levy RH. Stiripentol in atypical absence seizures in children: an open trial. Epilepsia 1993; 34(2): 305-11.

Fasching PA, Heusinger K, Haeberle L, Niklos M, Hein A, Bayer CM*, et al.* Ki67, chemotherapy response, and prognosis in breast cancer patients receiving neoadjuvant treatment. BMC Cancer 2011; 11: 486.

Fernandez Corcuera P, Pomarol E, Amann B, McKenna P. Myoclonus provoked by lamotrigine in a bipolar patient. J Clin Psychopharmacol 2008; 28(2): 248-9.

Forrer GR. Use of milontin in treating mental patients with petit mal convulsive disorders. J Mich State Med Soc 1954; 53(3): 275-7.

Foster LA, Johnson MR, MacDonald JT, Karachunski PI, Henry TR, Nascene DR*, et al.* Infantile Epileptic Encephalopathy Associated With SCN2A Mutation Responsive to Oral Mexiletine. Pediatr Neurol 2017; 66: 108-11.

French J, Smith M, Faught E, Brown L. Practice advisory: The use of felbamate in the treatment of patients with intractable epilepsy: report of the Quality Standards Subcommittee of the American Academy of Neurology and the American Epilepsy Society. Neurology 1999; 52(8): 1540-5.

Fromer M, Roussos P, Sieberts SK, Johnson JS, Kavanagh DH, Perumal TM*, et al.* Gene expression elucidates functional impact of polygenic risk for schizophrenia. Nat Neurosci 2016; 19(11): 1442-53.

Frye GD, McCown TJ, Breese GR, Peterson SL. GABAergic modulation of inferior colliculus excitability: role in the ethanol withdrawal audiogenic seizures. J Pharmacol Exp Ther 1986; 237(2): 478-85.

Gamaniel K, Wuorela H, Vapaatalo H. Effects of iloprost, prostaglandin E1 (PGE1) and prostacyclin (PGI2) on chemically and electrically induced seizures in mice. Prostaglandins Leukot Essent Fatty Acids 1989; 35(2): 63-8.

Garant DS, Xu SG, Sperber EF, Moshe SL. The influence of thalamic GABA transmission on the susceptibility of adult rats to flurothyl induced seizures. Epilepsy Res 1993; 15(3): 185-92.

Gaudreault J, Varin F, Pollack GM. Pharmacokinetics and anticonvulsant effect of a new hypnotic, CL 284,846, in rats. Pharm Res 1995; 12(11): 1592-7.

Gautam SK, Kulkarni SK. GABA/BZ-and NMDA-receptor interaction in digoxin-induced convulsions in rats. Indian J Exp Biol 1991; 29(7): 636-40.

Gelisse P, Genton P, Kuate C, Pesenti A, Baldy-Moulinier M, Crespel A. Worsening of seizures by oxcarbazepine in juvenile idiopathic generalized epilepsies. Epilepsia 2004; 45(10): 1282-6.

Gellman RL, Kallianos JA, McNamara JO. Alpha-2 receptors mediate an endogenous noradrenergic suppression of kindling development. J Pharmacol Exp Ther 1987; 241(3): 891-8.

Genton P. When antiepileptic drugs aggravate epilepsy. Brain Dev 2000; 22(2): 75-80.

Genton P, Gelisse P, Crespel A. Lack of efficacy and potential aggravation of myoclonus with lamotrigine in Unverricht-Lundborg disease. Epilepsia 2006; 47(12): 2083-5.

Georgiev VP, Lazarova MB, Kambourova TS. Further evidence for the interactions between angiotensin II and GABAergic transmission in pentylenetetrazol kindling seizures in mice. Neuropeptides 1995; 28(1): 29-34.

Gibbs EL, Gibbs FA, Fuster B. Psychomotor epilepsy. Arch Neurol Psychiatry 1948; 60(4): 331-9.

Gilbert ME, Mack CM. Enhanced susceptibility to kindling by chlordimeform may be mediated by a local anesthetic action. Psychopharmacology (Berl) 1989; 99(2): 163-7.

Gilson MK, Liu T, Baitaluk M, Nicola G, Hwang L, Chong J. BindingDB in 2015: A public database for medicinal chemistry, computational chemistry and systems pharmacology. Nucleic Acids Res 2016; 44(D1): D1045-53.

Gondran M, Eckeli AL, Migues PV, Gabilan NH, Rodrigues AL. The crude extract from the sea anemone, Bunodosoma caissarum elicits convulsions in mice: possible involvement of the glutamatergic system. Toxicon 2002; 40(12): 1667-74.

Gottlieb A, Altman RB. Integrating systems biology sources illuminates drug action. Clin Pharmacol Ther 2014; 95(6): 663-9.

Gottlieb A, Stein GY, Ruppin E, Sharan R. PREDICT: a method for inferring novel drug indications with application to personalized medicine. Mol Syst Biol 2011; 7: 496.

Gram L. Treatment of typical absences: acetazolamide, benzodiazepines and lamotrigine. Typical absences and related epileptic syndromes 1995: 368-75.

Grau J, Grosse I, Keilwagen J. PRROC: computing and visualizing precision-recall and receiver operating characteristic curves in R. Bioinformatics 2015; 31(15): 2595-7.

Groot VP, Gemenetzis G, Blair AB, Rivero-Soto RJ, Yu J, Javed AA*, et al.* Defining and Predicting Early Recurrence in 957 Patients With Resected Pancreatic Ductal Adenocarcinoma. Ann Surg 2019; 269(6): 1154-62.

Guerrini R, Belmonte A, Parmeggiani L, Perucca E. Myoclonic status epilepticus following high-dosage lamotrigine therapy. Brain Dev 1999; 21(6): 420-4.

Guerrini R, Dravet C, Genton P, Belmonte A, Kaminska A, Dulac O. Lamotrigine and seizure aggravation in severe myoclonic epilepsy. Epilepsia 1998; 39(5): 508-12.

Guney E, Menche J, Vidal M, Barabasi AL. Network-based in silico drug efficacy screening. Nat Commun 2016; 7: 10331.

Gusev A, Ko A, Shi H, Bhatia G, Chung W, Penninx BW*, et al.* Integrative approaches for large-scale transcriptome-wide association studies. Nat Genet 2016; 48(3): 245-52.

Gusev A, Mancuso N, Won H, Kousi M, Finucane HK, Reshef Y, et al. Transcriptome-wide association study of schizophrenia and chromatin activity yields mechanistic disease insights. Nat Genet 2018; 50(4): 538-48.

Hahn A, Fischenbeck A, Stephani U. Induction of epileptic negative myoclonus by oxcarbazepine in symptomatic epilepsy. Epileptic Disord 2004; 6(4): 271-4.

Hansen J, Auprich M, Ahyai SA, de la Taille A, van Poppel H, Marberger M*, et al.* Initial prostate biopsy: development and internal validation of a biopsy-specific nomogram based on the prostate cancer antigen 3 assay. Eur Urol 2013; 63(2): 201-9.

Herzog AG. Clomiphene therapy in epileptic women with menstrual disorders. Neurology 1988a; 38(3): 432-4.

Herzog AG. Seizure control with clomiphene therapy. A case report. Arch Neurol 1988b; 45(2): 209-10.

Hesdorffer DC, Stables JP, Hauser WA, Annegers JF, Cascino G. Are certain diuretics also anticonvulsants? Ann Neurol 2001; 50(4): 458-62.

Hess GP, Walson PD. Seizures secondary to oral viscous lidocaine. Ann Emerg Med 1988; 17(7): 725-7.

Holder JL, Jr., Wilfong AA. Zonisamide in the treatment of epilepsy. Expert Opin Pharmacother 2011; 12(16): 2573-81.

Holtkamp M. Other less commonly used antiepileptic drugs. The treatment of epilepsy 4th ed West Sussex: Wiley 2015: 689-700.

Hooshmand H. IntracSupplementary Table eizures: Treatment With a New Benzodiazepine Anticonvulsant. Archives of Neurology 1972; 27(3): 205-8.

Horn CS, Ater SB, Hurst DL. Carbamazepine-exacerbated epilepsy in children and adolescents. Pediatr Neurol 1986; 2(6): 340-5.

Horton R, Anlezark G, Meldrum B. Noradrenergic influences on sound-induced seizures. J Pharmacol Exp Ther 1980; 214(2): 437-42.

Hsieh XX, Hsu YC, Cherng CH, Lin CC, Huang GS, Lin SL*, et al.* Grand mal seizure induced by low-dose fentanyl and lidocaine in a young child. Acta Anaesthesiol Taiwan 2015; 53(3): 105-8.

Huang CW, Wu SN, Cheng JT, Tsai JJ, Huang CC. Diazoxide reduces status epilepticus neuron damage in diabetes. Neurotox Res 2010; 17(4): 305-16.

Ibhazehiebo K, Gavrilovici C, de la Hoz CL, Ma SC, Rehak R, Kaushik G, et al. A novel metabolism-based phenotypic drug discovery platform in zebrafish uncovers HDACs 1 and 3 as a potential combined anti-seizure drug target. Brain 2018; 141(3): 744-61.

Ibrahim FAS, Ghebremeskel K, Abdel-Rahman ME, Ahmed AAM, Mohmed IM, Osman G*, et al.* The differential effects of eicosapentaenoic acid (EPA) and docosahexaenoic acid (DHA) on seizure frequency in patients with drug-resistant epilepsy - A randomized, double-blind, placebo-controlled trial. Epilepsy Behav 2018; 87: 32-8.

Interleukin-6 Receptor Mendelian Randomisation Analysis C, Swerdlow DI, Holmes MV, Kuchenbaecker KB, Engmann JE, Shah T, et al. The interleukin-6 receptor as a target for prevention of coronary heart disease: a mendelian randomisation analysis. Lancet 2012; 379(9822): 1214-24.

International League Against Epilepsy Consortium on Complex E. Genome-wide mega-analysis identifies 16 loci and highlights diverse biological mechanisms in the common epilepsies. Nat Commun 2018; 9(1): 5269.

Issa NT, Kruger J, Wathieu H, Raja R, Byers SW, Dakshanamurthy S. DrugGenEx-Net: a novel computational platform for systems pharmacology and gene expression-based drug repurposing. BMC Bioinformatics 2016; 17(1): 202.

Ivanov S, Lagunin A, Filimonov D, Poroikov V. Assessment of the cardiovascular adverse effects of drug-drug interactions through a combined analysis of spontaneous reports and predicted drug-target interactions. PLoS Comput Biol 2019; 15(7): e1006851.

Jadhav AR, Vakade KP, Nayak BB, Sangisetti VM, Abhavathi VN. The effect of riluzole alone and in combination with sodium valproate on pentylenetetrazole induced seizures in swiss-albino rats. International Journal of Basic & Clinical Pharmacology 2016; 5(3): 728.

Janszky J, Rasonyi G, Halasz P, Olajos S, Perenyi J, Szucs A*, et al.* Disabling erratic myoclonus during lamotrigine therapy with high serum level--report of two cases. Clin Neuropharmacol 2000; 23(2): 86-9.

Jeavons PM. The effect of chlordiazepoxide on the electroencephalogram. Epilepsia 1962; 3: 110-6.

Jiang KW, Gao F, Shui QX, Yu ZS, Xia ZZ. Effect of diazoxide on regulation of vesicular and plasma membrane GABA transporter genes and proteins in hippocampus of rats subjected to picrotoxin-induced kindling. Neurosci Res 2004; 50(3): 319-29.

Jin G, Fu C, Zhao H, Cui K, Chang J, Wong ST. A novel method of transcriptional response analysis to facilitate drug repositioning for cancer therapy. Cancer Res 2012; 72(1): 33-44.

Kaddurah AK, Holmes GL. Possible precipitation of myoclonic seizures with oxcarbazepine. Epilepsy Behav 2006; 8(1): 289-93.

Kamal JA, Nadig RS, Joseph T, David J. Effect of calcium channel blockers on experimentally induced seizures in rats. Indian J Exp Biol 1990; 28(7): 605-8.

Kantarelis TD, Kantarelis D. In search of the criterion standard test in diagnostic testing. American Journal of Medical Research 2017; 4(1): 118.

Keiser MJ, Setola V, Irwin JJ, Laggner C, Abbas AI, Hufeisen SJ*, et al.* Predicting new molecular targets for known drugs. Nature 2009; 462(7270): 175-81.

Keränen T. Stiripentol. The Treatment of Epilepsy 2015: 628-32.

Kessler SK, McGinnis E. A Practical Guide to Treatment of Childhood Absence Epilepsy. Paediatr Drugs 2019; 21(1): 15-24.

Kim E, Choi AS, Nam H. Drug repositioning of herbal compounds via a machine-learning approach. BMC Bioinformatics 2019; 20(Suppl 10): 247.

Kim JE, Kim DS, Kwak SE, Choi HC, Song HK, Choi SY*, et al.* Anti-glutamatergic effect of riluzole: comparison with valproic acid. Neuroscience 2007; 147(1): 136-45.

Knake S, Hamer HM, Schomburg U, Oertel WH, Rosenow F. Tiagabine-induced absence status in idiopathic generalized epilepsy. Seizure 1999; 8(5): 314-7.

Kobayashi K, Ito M, Miyajima T, Fujii T, Okuno T. Successful management of intractable epilepsy with intravenous lidocain and lidocain tapes. Pediatr Neurol 1999; 21(1): 476-80.

Kobayashi K, Nishizawa Y, Sawada K, Ogura H, Miyabe M. K(+)-channel openers suppress epileptiform activities induced by 4-aminopyridine in cultured rat hippocampal neurons. J Pharmacol Sci 2008; 108(4): 517-28.

Kochen S, Giagante B, Oddo S. Spike-and-wave complexes and seizure exacerbation caused by carbamazepine. Eur J Neurol 2002; 9(1): 41-7.

Kodama M, Yamada N, Sato K, Sato T, Morimoto K, Kuroda S. The insular but not the perirhinal cortex is involved in the expression of fully-kindled amygdaloid seizures in rats. Epilepsy Res 2001; 46(2): 169-78.

Kohyama J, Shimohira M, Watanabe S, Fukuda C, Iwakawa Y. Mexiletine hydrochloride in an infant with intractable epilepsy. Brain Dev 1988; 10(4): 258-60.

Korberly BH, Mrazik TJ, Graziani LJ. Ethotoin use in pediatric seizure patients. Am J Dis Child 1981; 135(12): 1139-40.

Kotlinska J, Langwinski R. Involvement of opioid and other systems in ethanol abstinence audiogenic seizures in the rat? Pol J Pharmacol Pharm 1985; 37(2): 103-11.

Kryzhanovskii GN, Shandra AA. [Use of nicotinamide and pyridoxal-5-phosphate to treat experimental epilepsy]. Zh Nevropatol Psikhiatr Im S S Korsakova 1981; 81(6): 801-9.

Kryzhanovskii GN, Shandra AA, Godlevskii LS. [Effect of nicotinamide on generalized convulsions in mice]. Farmakol Toksikol 1982a; 45(6): 13-7.

Kryzhanovskii GN, Shandra AA, Godlevskii LS. [Effect of vitamin preparations on epileptic activity]. Biull Eksp Biol Med 1984; 98(8): 150-3.

Kryzhanovskii GN, Shandra AA, Godlevskii LS, Beliaeva AI. [Further study of the antiepileptic properties of nicotinamide]. Biull Eksp Biol Med 1981; 91(1): 42-5.

Kryzhanovskii GN, Shandra AA, Godlevskii LS, Nikushkin EV. [Effect of pantogam, nicotinamide, and phenazepam on seizure activity]. Biull Eksp Biol Med 1982b; 94(9): 61-4.

Kubova H, Haugvicova R, Mares P. Moderate anticonvulsant action of baclofen does not change during development. Biol Neonate 1996; 69(6): 405-12.

Kulkarni SK. Actions of clonidine on convulsions and behaviour. Arch Int Pharmacodyn Ther 1981; 252(1): 124-32.

Kumar KJ, Manjunath VG. Seizures following lignocaine administration. Indian Pediatr 2013; 50(5): 521-2.

Lapin IP. Nicotinamide, inosine and hypoxanthine, putative endogenous ligands of the benzodiazepine receptor, opposite to diazepam are much more effective against kynurenine-induced seizures than against pentylenetetrazol-induced seizures. Pharmacol Biochem Behav 1981; 14(5): 589-93.

Lazarova M, Samanin R. Potentiation by yohimbine of pentylenetetrazol-induced seizures in rats: role of alpha 2 adrenergic receptors. Pharmacol Res Commun 1983a; 15(4): 419-25.

Lazarova M, Samanin R. Serotonin mediation of the protective effect of clonidine against pentylenetetrazol-induced seizures in rats. Life Sci 1983b; 32(20): 2343-8.

Le Vann LJ. Chlordiazepoxide, a tranquillizer with anticonvulsant properties. Can Med Assoc J 1962; 86: 123-5.

Lee DL, Ayoub C, Shaw RK, Fontes ML. Grand mal seizure during cardiopulmonary bypass: probable lidocaine toxicity. J Cardiothorac Vasc Anesth 1999; 13(2): 200-2.

Lee T, Yoon Y. Drug repositioning using drug-disease vectors based on an integrated network. BMC Bioinformatics 2018; 19(1): 446.

Lembeck F, Beubler E. Convulsions induced by hyperbaric oxygen: inhibition by phenobarbital, diazepam and baclofen. Naunyn Schmiedebergs Arch Pharmacol 1977; 297(1): 47-51.

Lemere F. Milontin in treatment of petit mal epilepsy. Northwest Med 1954; 53(5): 482.

Lennox WG. The petit mal epilepsies; their treatment with tridione. J Am Med Assoc 1945; 129: 1069-74.

Lenz QF, Arroyo DS, Temp FR, Poersch AB, Masson CJ, Jesse AC*, et al.* Cysteinyl leukotriene receptor (CysLT) antagonists decrease pentylenetetrazol-induced seizures and blood-brain barrier dysfunction. Neuroscience 2014; 277: 859-71.

Leppik IE, White JR. Felbamate. The treatment of epilepsy 2015: 472-8.

Liberato JL, Cunha AO, Mortari MR, Gelfuso EA, Beleboni Rde O, Coutinho-Netto J*, et al.* Anticonvulsant and anxiolytic activity of FrPbAII, a novel GABA uptake inhibitor isolated from the venom of the social spider Parawixia bistriata (Araneidae: Araneae). Brain Res 2006; 1124(1): 19-27.

Liporace JD, Sperling MR, Dichter MA. Absence seizures and carbamazepine in adults. Epilepsia 1994; 35(5): 1026-8.

Liu L, Zheng T, Morris MJ, Wallengren C, Clarke AL, Reid CA*, et al.* The mechanism of carbamazepine aggravation of absence seizures. J Pharmacol Exp Ther 2006; 319(2): 790-8.

Liu X, Finucane HK, Gusev A, Bhatia G, Gazal S, O'Connor L, et al. Functional Architectures of Local and Distal Regulation of Gene Expression in Multiple Human Tissues. Am J Hum Genet 2017; 100(4): 605-16.

Login IS, Dreifuss FE. Anticonvulsant activity of clomiphene. Arch Neurol 1983; 40(8): 525.

Loiseau P. Stiripentol in absence seizures: an open study updated. Epilepsia 1989; 30: 639.

Lortie A, Chiron C, Mumford J, Dulac O. The potential for increasing seizure frequency, relapse, and appearance of new seizure types with vigabatrin. Neurology 1993; 43(11 Suppl 5): S24-7.

Loscher W, Czuczwar SJ. Comparison of drugs with different selectivity for central alpha 1-and alpha 2-adrenoceptors in animal models of epilepsy. Epilepsy Res 1987; 1(3): 165-72.

Loscher W, Lehmann H, Teschendorf HJ, Traut M, Gross G. Inhibition of monoamine oxidase type A, but not type B, is an effective means of inducing anticonvulsant activity in the kindling model of epilepsy. J Pharmacol Exp Ther 1999; 288(3): 984-92.

Lukawski K, Raszewski G, Czuczwar SJ. Interactions of aliskiren, a direct renin inhibitor, with antiepileptic drugs in the test of maximal electroshock in mice. Eur J Pharmacol 2018; 819: 108-13.

Lukawski K, Raszewski G, Czuczwar SJ. Effect of aliskiren, a direct renin inhibitor, on the protective action of antiepileptic drugs against pentylenetetrazole-induced clonic seizures in mice. Fundam Clin Pharmacol 2019; 33(2): 191-8.

Lundqvist M, Agren J, Hellstrom-Westas L, Flink R, Wickstrom R. Efficacy and safety of lidocaine for treatment of neonatal seizures. Acta Paediatr 2013; 102(9): 863-7.

Maitre M, Chesielski L, Lehmann A, Kempf E, Mandel P. Protective effect of adenosine and nicotinamide against audiogenic seizure. Biochem Pharmacol 1974; 23(20): 2807-16.

Malfatti CR, Perry ML, Schweigert ID, Muller AP, Paquetti L, Rigo FK*, et al.* Convulsions induced by methylmalonic acid are associated with glutamic acid decarboxylase inhibition in rats: a role for GABA in the seizures presented by methylmalonic acidemic patients? Neuroscience 2007; 146(4): 1879-87.

Mancuso N, Gayther S, Gusev A, Zheng W, Penney KL, Kote-Jarai Z, et al. Large-scale transcriptome-wide association study identifies new prostate cancer risk regions. Nat Commun 2018; 9(1): 4079.

Mansoori F, Rahgozar M, Kavousi K. FoPA: identifying perturbed signaling pathways in clinical conditions using formal methods. BMC Bioinformatics 2019; 20(1): 92.

Mantoan L, Walker M. Treatment options in juvenile myoclonic epilepsy. Curr Treat Options Neurol 2011; 13(4): 355-70.

Mares P, Slamberova R. Opposite effects of a GABA(B) antagonist in two models of epileptic seizures in developing rats. Brain Res Bull 2006; 71(1-3): 160-6.

Mares P, Tabashidze N. Contradictory effects of GABA-B receptor agonists on cortical epileptic afterdischarges in immature rats. Brain Res Bull 2008; 75(1): 173-8.

Marini C, Parmeggiani L, Masi G, D'Arcangelo G, Guerrini R. Nonconvulsive status epilepticus precipitated by carbamazepine presenting as dissociative and affective disorders in adolescents. J Child Neurol 2005; 20(8): 693-6.

Marson AG, Al-Kharusi AM, Alwaidh M, Appleton R, Baker GA, Chadwick DW*, et al.* The SANAD study of effectiveness of carbamazepine, gabapentin, lamotrigine, oxcarbazepine, or topiramate for treatment of partial epilepsy: an unblinded randomised controlled trial. Lancet 2007; 369(9566): 1000-15.

Marson AG, Sills GJ. Valproate. The treatment of Epilepsy 2015: 652-66.

Martinez-Lage J. Clinical antiepileptic efficacy of stiripentol in resistant partial epilepsies. Epilepsia 1984; 25: 673.

Masino AJ, Dechene ET, Dulik MC, Wilkens A, Spinner NB, Krantz ID*, et al.* Clinical phenotype-based gene prioritization: an initial study using semantic similarity and the human phenotype ontology. BMC Bioinformatics 2014; 15: 248.

McIntyre DC, Giugno L. Effect of clonidine on amygdala kindling in normal and 6-hydroxydopamine-pretreated rats. Exp Neurol 1988; 99(1): 96-106.

Michelucci R, Pasini E. Phenobarbital, primidone and other barbiturates. The Treatment of Epilepsy. London: John Wiley & Sons, Ltd; 2015. p. 555-73.

Michelucci R, Pasini E, Tassinari CA. Phenobarbital, primidone and other barbiturates. The treatment of epilepsy 2015: 555-73.

Millichap JG. Milontin: a new drug in the treatment of petit mal. Lancet 1952; 2(6741): 907-10.

Miyamoto A, Takahashi S, Oki J. [A successful treatment with intravenous lidocaine followed by oral mexiletine in a patient with Lennox-Gastaut syndrome]. No To Hattatsu 1999; 31(5): 459-64.

Mizuno H, Kitada K, Nakai K, Sarai A. PrognoScan: a new database for meta-analysis of the prognostic value of genes. BMC Med Genomics 2009; 2: 18.

Mlecnik B, Tosolini M, Charoentong P, Kirilovsky A, Bindea G, Berger A*, et al.* Biomolecular network reconstruction identifies T-cell homing factors associated with survival in colorectal cancer. Gastroenterology 2010; 138(4): 1429-40.

Moran LR, Hossain T, Insoft RM. Neonatal seizures following lidocaine administration for elective circumcision. J Perinatol 2004; 24(6): 395-6.

Mori K, Ito H, Toda Y, Hashimoto T, Miyazaki M, Saijo T*, et al.* Successful management of intractable epilepsy with lidocaine tapes and continuous subcutaneous lidocaine infusion. Epilepsia 2004; 45(10): 1287-90.

Mousavian Z, Khakabimamaghani S, Kavousi K, Masoudi-Nejad A. Drug-target interaction prediction from PSSM based evolutionary information. J Pharmacol Toxicol Methods 2016; 78: 42-51.

Mussi-Ribeiro A, Miranda A, Gobbo-Netto L, Peporine Lopes N, dos Santos WF. A anticonvulsive fraction from Scaptocosa raptoria (Araneae: Lycosidae) spider venom. Neurosci Lett 2004; 371(2-3): 171-5.

Mustard HS, Livingston S. Tridione therapy in epilepsy; a review of results in 156 patients with petit mal epilepsy with special reference to side reactions. J Pediatr 1949; 35(5): 540-5.

Nakazawa M, Okumura A, Niijima S, Yamashita S, Shimono K, Hirose S*, et al.* Oral mexiletine for lidocaine-responsive neonatal epilepsy. Brain Dev 2013; 35(7): 667-9.

Nelson SR. Effect of drugs on experimental brain edema in mice. J Neurosurg 1974; 41(2): 193-9.

Neufeld MY. Acetazolamide. The treatment of epilepsy 2015: 376-87.

Neufeld MY. Acetazolamide In: Shorvon S, Perucca E, Engel J, editors. The Treatment of Epilepsy. London: John Wiley & Sons, Ltd; 2016.

Nicoletti F, Speciale C, Sortino MA, Summa G, Caruso G, Patti F*, et al.* Comparative effects of estradiol benzoate, the antiestrogen clomiphene citrate, and the progestin medroxyprogesterone acetate on kainic acid-induced seizures in male and female rats. Epilepsia 1985; 26(3): 252-7.

Nobeli I, Favia AD, Thornton JM. Protein promiscuity and its implications for biotechnology. Nat Biotechnol 2009; 27(2): 157-67.

Ohata K, Murata T, Sakamoto H, Inoue K, Kobayashi M, Kohno S*, et al.* [Pharmacological studies of guanabenz: effects of the central nervous system]. Nihon Yakurigaku Zasshi 1982; 80(6): 471-80.

Ohno Y, Sofue N, Imaoku T, Morishita E, Kumafuji K, Sasa M*, et al.* Serotonergic modulation of absence-like seizures in groggy rats: a novel rat model of absence epilepsy. J Pharmacol Sci 2010; 114(1): 99-105.

Okada Y, Wu D, Trynka G, Raj T, Terao C, Ikari K*, et al.* Genetics of rheumatoid arthritis contributes to biology and drug discovery. Nature 2014; 506(7488): 376-81.

Omrani S, Taheri M, Omrani MD, Arsang-Jang S, Ghafouri-Fard S. The effect of omega-3 fatty acids on clinical and paraclinical features of intractable epileptic patients: a triple blind randomized clinical trial. Clin Transl Med 2019; 8(1): 3.

Ormandy GC, Song L, Jope RS. Analysis of the convulsant-potentiating effects of lithium in rats. Exp Neurol 1991; 111(3): 356-61.

Osorio I, Reed RC, Peltzer JN. Refractory idiopathic absence status epilepticus: A probable paradoxical effect of phenytoin and carbamazepine. Epilepsia 2000; 41(7): 887-94.

Ozbakis-Dengiz G, Bakirci A. Anticonvulsant and hypnotic effects of amiodarone. J Zhejiang Univ Sci B 2009; 10(4): 317-22.

Pacey LK, Heximer SP, Hampson DR. Increased GABA(B) receptor-mediated signaling reduces the susceptibility of fragile X knockout mice to audiogenic seizures. Mol Pharmacol 2009; 76(1): 18-24.

Palencia G, Calderon A, Sotelo J. Thalidomide inhibits pentylenetetrazole-induced seizures. J Neurol Sci 2007; 258(1-2): 128-31.

Palencia G, Rubio C, Custodio-Ramirez V, Paz C, Sotelo J. Strong anticonvulsant effect of thalidomide on amygdaloid kindling. Epilepsy Res 2011; 95(3): 263-9.

Palmer GC, Harris EW, Ray R, Stagnitto ML, Schmiesing RJ. Classification of compounds for prevention of NMDLA-induced seizures/mortality, or maximal electroshock and pentylenetetrazol seizures in mice and antagonism of MK801 binding in vitro. Arch Int Pharmacodyn Ther 1992; 317: 16-34.

Panayiotopoulos CP. Typical absence seizures and their treatment. Archives of Disease in Childhood 1999; 81: 351-5.

Pantuck AJ, Goldsmith JW, Kuriyan JB, Weiss RE. Seizures after ureteral stone manipulation with lidocaine. J Urol 1997; 157(6): 2248.

Pappinen S, Pasonen-Seppanen S, Suhonen M, Tammi R, Urtti A. Rat epidermal keratinocyte organotypic culture (ROC) as a model for chemically induced skin irritation testing. Toxicol Appl Pharmacol 2005; 208(3): 233-41.

Park JO, Qin LX, Prete FP, Antonescu C, Brennan MF, Singer S. Predicting outcome by growth rate of locally recurrent retroperitoneal liposarcoma: the one centimeter per month rule. Ann Surg 2009; 250(6): 977-82.

Parker AP, Agathonikou A, Robinson RO, Panayiotopoulos CP. Inappropriate use of carbamazepine and vigabatrin in typical absence seizures. Dev Med Child Neurol 1998; 40(8): 517-9.

Parmeggiani A, Fraticelli E, Rossi PG. Exacerbation of epileptic seizures by carbamazepine: report of 10 cases. Seizure 1998; 7(6): 479-83.

Payandemehr B, Rahimian R, Gooshe M, Bahremand A, Gholizadeh R, Berijani S*, et al.* Nitric oxide mediates the anticonvulsant effects of thalidomide on pentylenetetrazole-induced clonic seizures in mice. Epilepsy Behav 2014; 34: 99-104.

Pearl PL, Holmes GL. Childhood Absence Epilepsies. In: Pellock JM, Bourgeois BFD, Dodson EW, Nordli DR, Sankar R, editors. Pediatric Epilepsy: Diagnosis and Therapy, 3rd edition. New York: Demos Medical Publishing; 2008. p. 323-34.

Pelletier MR, Corcoran ME. Intra-amygdaloid infusions of clonidine retard kindling. Brain Res 1992; 598(1-2): 51-8.

Pellock JM, Faught E, Leppik IE, Shinnar S, Zupanc ML. Felbamate: consensus of current clinical experience. Epilepsy Res 2006; 71(2-3): 89-101.

Peterson RT. Chemical biology and the limits of reductionism. Nat Chem Biol 2008; 4(11): 635-8.

Pickering A. ccmap: Combination Connectivity Mapping. 2017.

Plotnikoff N, Huang J, Havens P. Effect of monoamino oxidase inhibitors on audiogenic seizures. Journal of pharmaceutical sciences 1963; 52(2): 172-3.

Prasad AN, Stefanelli M, Nagarajan L. Seizure exacerbation and developmental regression with carbamazepine. Can J Neurol Sci 1998; 25(4): 287-94.

Proschak E, Stark H, Merk D. Polypharmacology by Design: A Medicinal Chemist's Perspective on Multitargeting Compounds. J Med Chem 2019; 62(2): 420-44.

Rayhan F, Ahmed S, Md Farid D, Dehzangi A, Shatabda S. CFSBoost: Cumulative feature subspace boosting for drug-target interaction prediction. J Theor Biol 2019; 464: 1-8.

Rayhan F, Ahmed S, Shatabda S, Farid DM, Mousavian Z, Dehzangi A*, et al.* iDTI-ESBoost: Identification of Drug Target Interaction Using Evolutionary and Structural Features with Boosting. Sci Rep 2017; 7(1): 17731.

Read MI, Andreianova AA, Harrison JC, Goulton CS, Sammut IA, Kerr DS. Cardiac electrographic and morphological changes following status epilepticus: effect of clonidine. Seizure 2014; 23(1): 55-61.

Read MI, Harrison JC, Kerr DS, Sammut IA. Atenolol offers better protection than clonidine against cardiac injury in kainic acid-induced status epilepticus. Br J Pharmacol 2015; 172(19): 4626-38.

Resar LM, Helfaer MA. Recurrent seizures in a neonate after lidocaine administration. J Perinatol 1998; 18(3): 193-5.

Rezvani M, Finkelstein Y, Verjee Z, Railton C, Koren G. Generalized seizures following topical lidocaine administration during circumcision: establishing causation. Paediatr Drugs 2007; 9(2): 125-7.

Riaz A, Matsuo F. Lamotrigine. The treatment of epilepsy 2015: 498-515.

Richardson TG, Hemani G, Gaunt TR, Relton CL, Davey Smith G. A transcriptome-wide Mendelian randomization study to uncover tissue-dependent regulatory mechanisms across the human phenome. Nat Commun 2020; 11(1): 185.

Sakakihara Y, Oka A, Kubota M, Ohashi Y. Reduction of seizure frequency with clomipramine in patients with complex partial seizures. Brain Dev 1995; 17(4): 291-3.

Sanseau P, Agarwal P, Barnes MR, Pastinen T, Richards JB, Cardon LR*, et al.* Use of genome-wide association studies for drug repositioning. Nat Biotechnol 2012; 30(4): 317-20.

Santos R, Ursu O, Gaulton A, Bento AP, Donadi RS, Bologa CG*, et al.* A comprehensive map of molecular drug targets. Nat Rev Drug Discov 2017; 16(1): 19-34.

Schmidt AF, Finan C, Gordillo-Maranon M, Asselbergs FW, Freitag DF, Patel RS, et al. Genetic drug target validation using Mendelian randomisation. Nat Commun 2020; 11(1): 3255.

Scotti de Carolis A, Passarelli F, Pezzola A. Study on the anticonvulsant activity of clonidine against pentylenetetrazol-induced seizures in rats: pharmacological evidence of alpha 2-adrenoceptors mediation. Arch Int Pharmacodyn Ther 1986; 282(2): 209-18.

Serafini A, Gerard E, Genton P, Crespel A, Gelisse P. Treatment of Juvenile Myoclonic Epilepsy in Patients of Child-Bearing Potential. CNS Drugs 2019; 33(3): 195-208.

Shafaroodi H, Moezi L, Bahremand A, Dehpour AR. The role of alpha(2)-adrenoceptors in the anti-convulsant effects of cannabinoids on pentylenetetrazole-induced seizure threshold in mice. Eur J Pharmacol 2013; 714(1-3): 1-6.

Shaked I, Oberhardt MA, Atias N, Sharan R, Ruppin E. Metabolic Network Prediction of Drug Side Effects. Cell Syst 2016; 2(3): 209-13.

Shany E, Benzaqen O, Watemberg N. Comparison of continuous drip of midazolam or lidocaine in the treatment of intractable neonatal seizures. J Child Neurol 2007; 22(3): 255-9.

Sheikhi M, Shirzadian A, Dehdashtian A, Amiri S, Ostadhadi S, Ghasemi M*, et al.* Involvement of ATP-sensitive potassium channels and the opioid system in the anticonvulsive effect of zolpidem in mice. Epilepsy Behav 2016; 62: 291-6.

Shields WD, Saslow E. Myoclonic, atonic, and absence seizures following institution of carbamazepine therapy in children. Neurology 1983; 33(11): 1487-9.

Shorvon SD. Drug treatment of epilepsy in the century of the ILAE: the first 50 years, 1909-1958. Epilepsia 2009a; 50 Suppl 3: 69-92.

Shorvon SD. Drug treatment of epilepsy in the century of the ILAE: the second 50 years, 1959-2009. Epilepsia 2009b; 50 Suppl 3: 93-130.

Shorvon SDe, Perucca Ee, Engel JJe. The treatment of epilepsy. Fourth edition. ed.

Shouse MN, Scordato JC, Farber PR, de Lanerolle N. The alpha2 adrenoreceptor agonist clonidine suppresses evoked and spontaneous seizures, whereas the alpha2 adrenoreceptor antagonist idazoxan promotes seizures in amygdala-kindled kittens. Brain Res 2007; 1137(1): 58-68.

Simillion C, Liechti R, Lischer HE, Ioannidis V, Bruggmann R. Avoiding the pitfalls of gene set enrichment analysis with SetRank. BMC Bioinformatics 2017; 18(1): 151.

Skardoutsou A, Voudris KA, Vagiakou EA. Non-convulsive status epilepticus associated with tiagabine therapy in children. Seizure 2003; 12(8): 599-601.

Smith M, Wolfram W, Rose R. Toxicity--seizures in an infant caused by (or related to) oral viscous lidocaine use. J Emerg Med 1992; 10(5): 587-90.

Snead OC, 3rd, Hosey LC. Exacerbation of seizures in children by carbamazepine. N Engl J Med 1985; 313(15): 916-21.

So EL, Ruggles KH, Cascino GD, Ahmann PA, Weatherford KW. Seizure exacerbation and status epilepticus related to carbamazepine-10,11-epoxide. Ann Neurol 1994; 35(6): 743-6.

So HC, Chau CK, Chiu WT, Ho KS, Lo CP, Yim SH*, et al.* Analysis of genome-wide association data highlights candidates for drug repositioning in psychiatry. Nat Neurosci 2017; 20(10): 1342-9.

So HC, Chau CK, Lau A, Wong SY, Zhao K. Translating GWAS findings into therapies for depression and anxiety disorders: gene-set analyses reveal enrichment of psychiatric drug classes and implications for drug repositioning. Psychol Med 2018: 1-17.

Somerville ER. Some treatments cause seizure aggravation in idiopathic epilepsies (especially absence epilepsy). Epilepsia 2009; 50 Suppl 8: 31-6.

Sperber EF, Wurpel JN, Moshe SL. Evidence for the involvement of nigral GABAB receptors in seizures of rat pups. Brain Res Dev Brain Res 1989; 47(1): 143-6.

Stone B, Evans L, Coleman J, Kuebler D. Genetic and pharmacological manipulations that alter metabolism suppress seizure-like activity in Drosophila. Brain Res 2013; 1496: 94-103.

Storm CS, Kia DA, Almramhi M, Bandres-Ciga S, Finan C, Hingorani AD, et al. Finding drug targeting mechanisms with genetic evidence for Parkinson’s disease. bioRxiv 2020: 2020.07.24.208975.

Subramanian A, Narayan R, Corsello SM, Peck DD, Natoli TE, Lu X*, et al.* A Next Generation Connectivity Map: L1000 Platform and the First 1,000,000 Profiles. Cell 2017; 171(6): 1437-52 e17.

Sun H, Shahane S, Xia M, Austin CP, Huang R. Structure based model for the prediction of phospholipidosis induction potential of small molecules. J Chem Inf Model 2012; 52(7): 1798-805.

Sun XY, Zhang L, Wei CX, Piao HR, Quan ZS. Characterization of the anticonvulsant activity of doxepin in various experimental seizure models in mice. Pharmacol Rep 2009; 61(2): 245-51.

Sundaram MB. Seizures after intraurethral instillation of lidocaine. CMAJ 1987; 137(3): 219-20.

Tacke U, Kolonen S. The effect of clonidine and yohimbine on audiogenic seizures (AGS) in rats. Pharmacol Res Commun 1984; 16(10): 1019-30.

Taha AY, Huot PS, Reza-Lopez S, Prayitno NR, Kang JX, Burnham WM*, et al.* Seizure resistance in fat-1 transgenic mice endogenously synthesizing high levels of omega-3 polyunsaturated fatty acids. J Neurochem 2008; 105(2): 380-8.

Takahashi Y, Imai K, Ikeda H, Kubota Y, Yamazaki E, Susa F. Open study of pranlukast add-on therapy in intractable partial epilepsy. Brain Dev 2013; 35(3): 236-44.

Talwar D, Arora MS, Sher PK. EEG changes and seizure exacerbation in young children treated with carbamazepine. Epilepsia 1994; 35(6): 1154-9.

Tanoli Z, Alam Z, Vaha-Koskela M, Ravikumar B, Malyutina A, Jaiswal A*, et al.* Drug Target Commons 2.0: a community platform for systematic analysis of drug-target interaction profiles. Database (Oxford) 2018; 2018: 1-13.

Tarca AL, Bhatti G, Romero R. A comparison of gene set analysis methods in terms of sensitivity, prioritization and specificity. PLoS One 2013; 8(11): e79217.

Teramukai S, Kitano T, Kishida Y, Kawahara M, Kubota K, Komuta K*, et al.* Pretreatment neutrophil count as an independent prognostic factor in advanced non-small-cell lung cancer: an analysis of Japan Multinational Trial Organisation LC00-03. European journal of cancer 2009; 45(11): 1950-8.

Thompson RG, Aldrete JA. Interaction between local anesthetics and analeptic drugs. South Med J 1975; 68(2): 189-92.

Tombini M, Pellegrino G, Assenza G, Di Lazzaro V. De novo multifocal myoclonus induced by lamotrigine in a temporal lobe epilepsy case. J Neurol Sci 2017; 373: 31-2.

Trinka E, Brigo F. Benzodiazepines used in the treatment of epilepsy. The Treatment of Epilepsy, S Shorvon, E Perucca, J Engel, Eds(John Wiley & Sons, Ltd, 2015) 2015: 398-417.

Trojnar MK, Wojtal K, Trojnar MP, Czuczwar SJ. Stiripentol. A novel antiepileptic drug. Pharmacol Rep 2005; 57(2): 154-60.

Troupin AS, Ojemann LM, Dodrill CB. Mephenytoin: a reappraisal. Epilepsia 1976; 17(4): 403-14.

Trudeau V, Myers S, LaMoreaux L, Anhut H, Garofalo E, Ebersole J. Gabapentin in naive childhood absence epilepsy: results from two double-blind, placebo-controlled, multicenter studies. J Child Neurol 1996; 11(6): 470-5.

Turski L, Niemann W, Stephens DN. Differential effects of antiepileptic drugs and beta-carbolines on seizures induced by excitatory amino acids. Neuroscience 1990; 39(3): 799-807.

Tyurenkov IN, Borodkina LE, Bagmetova VV, Berestovitskaya VM, Vasil'eva OS. Comparison of Nootropic and Neuroprotective Features of Aryl-Substituted Analogs of Gamma-Aminobutyric Acid. Bull Exp Biol Med 2016; 160(4): 465-9.

Uemura S, Kimura H. Amygdaloid kindling with bicuculline methiodide in rats. Exp Neurol 1988; 102(3): 346-53.

Usubiaga JE, Wikinski J, Ferrero R, Usubiaga LE, Wikinski R. Local anesthetic-induced convulsions in man--an electroencephalographic study. Anesth Analg 1966; 45(5): 611-20.

Vanniyasingam T, Rodseth RN, Lurati Buse GA, Bolliger D, Burkhart CS, Cuthbertson BH*, et al.* Predicting the occurrence of major adverse cardiac events within 30 days of a vascular surgery: an empirical comparison of the minimum p value method and ROC curve approach using individual patient data meta-analysis. Springerplus 2016; 5: 304.

Veliskova J, Velisek L, Moshe SL. Age-specific effects of baclofen on pentylenetetrazol-induced seizures in developing rats. Epilepsia 1996; 37(8): 718-22.

Vendrame M, Khurana DS, Cruz M, Melvin J, Valencia I, Legido A*, et al.* Aggravation of seizures and/or EEG features in children treated with oxcarbazepine monotherapy. Epilepsia 2007; 48(11): 2116-20.

Vlainic J, Pericic D. Effects of acute and repeated zolpidem treatment on pentylenetetrazole-induced seizure threshold and on locomotor activity: comparison with diazepam. Neuropharmacology 2009; 56(8): 1124-30.

Vlainic J, Pericic D. Zolpidem is a potent anticonvulsant in adult and aged mice. Brain Res 2010; 1310: 181-8.

Vollmar C, Noachtar S. Tiagabine-induced myoclonic status epilepticus in a nonepileptic patient. Neurology 2007; 68(4): 310.

Vossler DG. Exacerbation of seizures in Lennox-Gastaut syndrome by gabapentin. Neurology 1996; 46(3): 852-3.

Vrielynck P. Current and emerging treatments for absence seizures in young patients. Neuropsychiatr Dis Treat 2013; 9: 963-75.

Wain LV, Shrine N, Artigas MS, Erzurumluoglu AM, Noyvert B, Bossini-Castillo L*, et al.* Genome-wide association analyses for lung function and chronic obstructive pulmonary disease identify new loci and potential druggable targets. Nat Genet 2017; 49(3): 416-25.

Walker MC, Shorvon SD. Emergency treatment of seizures and status epilepticus. The treatment of epilepsy 2015; 2: 221-44.

Wallengren C, Li S, Morris MJ, Jupp B, O'Brien TJ. Aggravation of absence seizures by carbamazepine in a genetic rat model does not induce neuronal c-Fos activation. Clin Neuropharmacol 2005; 28(2): 60-5.

Wallenstein MC, Mauss EA. Effect of prostaglandin synthetase inhibitors on experimentally induced convulsions in rats. Pharmacology 1984; 29(2): 85-93.

Wang YF, Zhang Y, Zhu Z, Wang TY, Morris DL, Shen JJ*, et al.* Identification of ST3AGL4, MFHAS1, CSNK2A2 and CD226 as loci associated with systemic lupus erythematosus (SLE) and evaluation of SLE genetics in drug repositioning. Ann Rheum Dis 2018; 77(7): 1078-84.

Watanabe K, Taskesen E, van Bochoven A, Posthuma D. Functional mapping and annotation of genetic associations with FUMA. Nat Commun 2017; 8(1): 1826.

Watson CW, Bowker R, Calish C. Effect of Chlordiazepoxide on Epileptic Seizures. JAMA 1964; 188(3): 212-6.

Weeke LC, Toet MC, van Rooij LG, Groenendaal F, Boylan GB, Pressler RM*, et al.* Lidocaine response rate in aEEG-confirmed neonatal seizures: Retrospective study of 413 full-term and preterm infants. Epilepsia 2016; 57(2): 233-42.

Williams DM, Finan C, Schmidt AF, Burgess S, Hingorani AD. Lipid lowering and Alzheimer disease risk: A mendelian randomization study. Ann Neurol 2020; 87(1): 30-9.

Wong E, Nahar N, Hau E, Varikatt W, Gebski V, Ng T*, et al.* Cut-point for Ki-67 proliferation index as a prognostic marker for glioblastoma. Asia Pac J Clin Oncol 2019; 15(1): 5-9.

Wurpel JN. Baclofen prevents rapid amygdala kindling in adult rats. Experientia 1994; 50(5): 475-8.

Wurpel JN, Sperber EF, Moshe SL. Baclofen inhibits amygdala kindling in immature rats. Epilepsy Res 1990; 5(1): 1-7.

Xiao Y, Li X. Polyunsaturated fatty acids modify mouse hippocampal neuronal excitability during excitotoxic or convulsant stimulation. Brain Res 1999; 846(1): 112-21.

Yamamoto Y, Ikoma H, Morimura R, Konishi H, Murayama Y, Komatsu S*, et al.* Optimal duration of the early and late recurrence of hepatocellular carcinoma after hepatectomy. World J Gastroenterol 2015; 21(4): 1207-15.

Yan QS, Dailey JW, Steenbergen JL, Jobe PC. Anticonvulsant effect of enhancement of noradrenergic transmission in the superior colliculus in genetically epilepsy-prone rats (GEPRs): a microinjection study. Brain Res 1998; 780(2): 199-209.

Yanagihara K, Otani K, Goto M, Futagi Y. [The effect of lidocaine tapes on cluster of intractable partial seizures]. No To Hattatsu 1996; 28(4): 352-4.

Yoon JR, Lee EJ, Kim HD, Lee JH, Kang HC. Polyunsaturated fatty acid-enriched diet therapy for a child with epilepsy. Brain Dev 2014; 36(2): 163-6.

Yoshida M, Noguchi E, Tsuru N, Ohkoshi N. Effect of riluzole on the acquisition and expression of amygdala kindling. Epilepsy Res 2001; 46(2): 101-9.

Young D, Dragunow M. Status epilepticus may be caused by loss of adenosine anticonvulsant mechanisms. Neuroscience 1994; 58(2): 245-61.

Yozawitz EG, Moshé SL. Management of Epilepsy in Neonates and Infants. The Treatment of Epilepsy: Wiley Online Library; 2015. p. 156-73.

Yuen AW, Sander JW, Fluegel D, Patsalos PN, Bell GS, Johnson T*, et al.* Omega-3 fatty acid supplementation in patients with chronic epilepsy: a randomized trial. Epilepsy Behav 2005; 7(2): 253-8.

Zaitsev AV, Kim K, Vasilev DS, Lukomskaya NY, Lavrentyeva VV, Tumanova NL, et al. N-methyl-D-aspartate receptor channel blockers prevent pentylenetetrazole-induced convulsions and morphological changes in rat brain neurons. J Neurosci Res 2015; 93(3): 454-65.

Zaluska K, Kondrat-Wrobel MW, Luszczki JJ. Comparison of the anticonvulsant potency of various diuretic drugs in the maximal electroshock-induced seizure threshold test in mice. Adv Clin Exp Med 2018; 27(5): 609-13.

Zgrajka W, Nieoczym D, Czuczwar M, Kis J, Brzana W, Wlaz P*, et al.* Evidences for pharmacokinetic interaction of riluzole and topiramate with pilocarpine in pilocarpine-induced seizures in rats. Epilepsy Res 2010; 88(2-3): 269-74.

Zhai J, Tang Y, Yuan H, Wang L, Shang H, Ma C. A Meta-Analysis Based Method for Prioritizing Candidate Genes Involved in a Pre-specific Function. Front Plant Sci 2016; 7: 1914.

Zhang W, Bai Y, Wang Y, Xiao W. Polypharmacology in Drug Discovery: A Review from Systems Pharmacology Perspective. Curr Pharm Zhu H, Zhou X. Transcriptome-wide association studies: a view from Mendelian randomization. Quantitative Biology 2020: 1-15. Des 2016; 22(21): 3171-81.

Zhu Y, Vaughn BV. Non-convulsive status epilepticus induced by tiagabine in a patient with pseudoseizure. Seizure 2002; 11(1): 57-9.

Zimmerman FT. Milontin and other new drugs in the treatment of petit mal epilepsy. South Med J 1954; 47(10): 929-35.
